# 4-Furanylvinylquinoline derivative as a new scaffold for the design of oxidative stress initiator and glucose transporter inhibitor drugs

**DOI:** 10.1038/s41598-024-79698-0

**Published:** 2024-11-18

**Authors:** Michał Kuczak, Wioleta Cieślik, Robert Musioł, Anna Mrozek-Wilczkiewicz

**Affiliations:** 1 A. Chelkowski Institute of Physics, University of Silesia in Katowice, 75 Pulku Piechoty 1a, Chorzow, 41- 500 Poland; 2 Institute of Chemistry, University of Silesia in Katowice, Szkolna 9, Katowice, 40-006 Poland; 3 Present Address: Department of Systems Biology and Engineering, Silesian University of Technology, Akademicka 2A, Gliwice, 44-100 Poland

**Keywords:** Furanylvinylquinolines, Cell cycle inhibition, p53 mutation, Apoptosis, Oxidative stress, GLUT inhibitor, Warburg effect, Biomedical engineering, Medicinal chemistry, Drug development

## Abstract

In the present study, a detailed analysis of the effect of a substitution at the C4 position of the quinoline ring by styryl or furanylvinyl substituents on the structure-antitumour activity relationship was conducted. After analysing a library of derivatives from the styrylquinoline and furanylvinylquinoline groups, we selected the most active (IC_50_ below 100 nM) derivative **13**, which contained the strongly electron-withdrawing nitro group in the furan substituent. The mechanism of action of this compound was studied on cell lines that differed in their p53 protein status. For this derivative, both cell cycle arrest (in G2/M phase in both HCT 116 cell lines and S phase for U-251 cell line) and the induction of apoptosis (up to 66% for U-251 cell line) were revealed. These studies were then confirmed by other methods at the gene and protein levels. Interestingly, we observed differences in the mechanism of action depending on the presence and mutation of the p53 protein, thus confirming its key role in cellular processes. Incubation with derivative **13** resulted in the induction of oxidative stress and triggered a cascade of cellular defence proteins that failed in the face of such an active compound. In addition, the results showed an inhibition of the GLUT-1 glucose transporter, which is extremely important in the context of anti-cancer activity.

**Supplementary Information:**

The online version contains supplementary material available at 10.1038/s41598-024-79698-0.

## Introduction

**Fig. 1:**
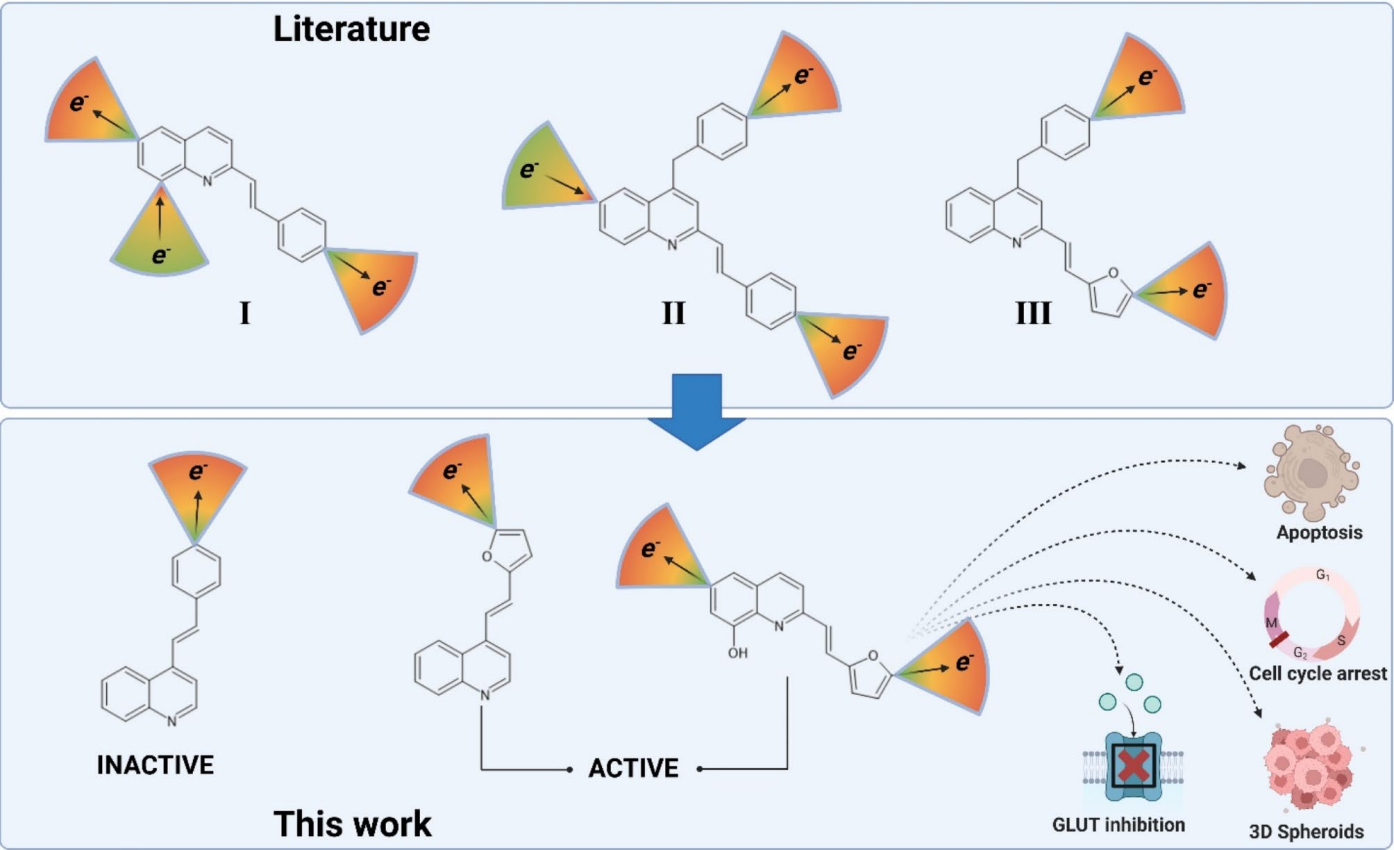
Schematic idea of a current work. Active styrylquinoline (**I**, **II**) and furanylvinylquinoline (**III**)structural features combined to design new derivatives – substituted at the C2 and C4 positions of quinoline. Color cones represent electro-accepting (nitro, cyano, carboxylic) and -donating (amine, hydroxyl) groups. Created with BioRender.com.

The styrylquinoline derivatives (**I**) have been of interest to our group for years in the context of their anticancer activity. In one of the previous papers, antiproliferative studies were conducted on leukaemia and neuroepithelioma cancer cell lines^1^. In this work active derivatives had a electroaccepting substituents in styryl part and electrodonating at C8 of quinoline moiety. Similar situation was described in work of El-Sayed et al. where electroaccepting groups were located at C6^2^. These compounds (**II**) designed to inhibit the epidermal growth factor receptor (EGFR) possessed also more developed aromatic fragments at C4 position of quinoline. The antitumour activity was confirmed on the HepG2 and HCT116 cell lines over an IC_50_ range from a few to several mg/mL, which was comparable to the reference compounds employed (5-fluorouracil and afatinib). It was also discussed substitution at position 2 of quinoline in-depth in another paper^3^, where we examined a wide library of compounds (**I**) and analysed the effect of substituents on the anticancer activity of the whole molecule. In this study, we showed that a hydroxyl or acetoxy group at the C-8 position of quinoline and strongly electron-withdrawing substituents in the phenyl ring of the styryl part are crucial for an elevated level of anticancer activity of the 2-styrylquinolines. Of particular interest was the NO_2_ group, for which the IC_50_ reached several µM. In addition to the aspects that are related to studies on pharmacophores, in a previous work^3^, we focused on the mutation of the p53 protein, which plays a key role in many cellular processes. This mutation is important in the extended investigations presented in the present study, in which we analysed new styrylquinoline and furanylvinylquinoline derivatives substituted at positions 2 and 4 including a previously unexplored furan substituent in position 4 of the quinoline (Fig. 1).

A study by Chang presented compounds with a substituted furan at the phenyl site (**III**), which showed a higher level of activity than those with a phenyl ring^4^. As was also mentioned, a significant effect of an electron-withdrawing substituent (NO_2_) was shown to improve the ability to inhibit cancer cell proliferation^5^. Interestingly, only a few reports on this topic were found when reviewing the recent literature on furanylvinylquinoline derivatives. In 1966 and 1974, there were reports on the activity of such derivatives substituted at position 2 of the quinoline that had been tested on a mouse model (**III**)^6,7^. A good reference to the results presented in this paper concerning the generation of oxidative stress is reference^8^, which describes the oxidative properties of derivatives with the general formula (**III**). Combination of these facts prompted us to design quinolines substituted with furanylvinyl fragments.

Moreover the biological testing schedule was also designed according to former literature reports. In previous investigations on styrylquinoline a p53 independent mechanism of action was suggested^9^. On the other hand strong reactive oxygen species (ROS) have been noticed^10^. Therefore we decided to investigate cell lines with different TP53 status and include the oxidative stress response in the schedule. Excessive levels of ROS stimulate the signals that regulate the expression of the glucose transporters (GLUT) proteins, thereby inducing glucose uptake^11^. This is a cellular response to the induced oxidative stress. Similar aspect has been studied in work of Pastuch-Gawolek et al. where quinoline glycoconjugates exert their activity through ROS and disrupting antioxidative cellular system^12^.

Among other things, it manifests itself in the reprogramming of glycolysis toward increased lactate production even in the presence of oxygen^13^. Together with an increased glucose uptake, which is caused by overexpressing glucose receptors^14^, this leads to the excessive production of the growth factors that are necessary for enhanced proliferation^15^. This anomaly, which was discovered more than 100 years ago, is called the Warburg effect in honour of the Nobel laureate, who made a significant contribution to understanding the mechanism of cancer cell respiration^16^. The disturbed homeostasis of tumour cells is due, among other things, to the more frequent occurrence of the oxidative processes^17^.

## Materials and methods

All of the reagents were purchased from Aldrich. TLC experiments were performed on alumina-backed silica gel 460 F_254_ plates (Merck, Darmstadt, Germany). The plates were illuminated under UV (254 nm) and evaluated in iodine vapor. The melting points were determined on an OptiMelt MPA100 apparatus (SRS, Sunnyvale, CA). All ^1^H and ^13^C NMR spectra were recorded on a Bruker Avance III 500 MHz FT-NMR spectrometer (500 MHz for ^1^H and 101/126 MHz for ^13^C, Bruker, Karlsruhe, Germany). Chemical shifts are reported in ppm (δ) using the signal of the solvent (DMSO-*d*_*6*_) as the reference against the Si(CH_3_)_4_ internal standard. Easily exchangeable signals were omitted when they were diffuse. Signals are designated as follows: s, singlet; d, doublet; dd, doublet of doublets; ddd, doublet of doublet of doublets; t, triplet; tt, triplet of triplets; td, triplet of doublets; and m, multiplet. Mass spectra were measured using an LTQ Orbitrap Hybrid Mass Spectrometer (Thermo Fisher Scientific, USA) with direct injection into an APCI source (400 °C) in the positive mode.

### Synthesis of compounds

The compounds were prepared in the following manner:

#### General procedure

***Method A***: The appropriate quinoline derivative (2.5 mmol) in acetic anhydride with 80% acetic acid (3:1) was thoroughly mixed with one equivalent of aldehyde (2.5 mmol) and heated in an inert gas atmosphere (N_2_) for 18–22 h at 130 °C. The mixture was then evaporated to dryness, and the resulting solid was crystallized from ethanol, methanol, or ethyl acetate.

***Method B***: The appropriate quinoline derivative (2.5 mmol) in acetic anhydride with 80% acetic acid (3:1) was thoroughly mixed with one equivalent of aldehyde (2.5 mmol) and heated in an inert gas atmosphere (N_2_) for 18–22 h at 130 °C. The liquid was evaporated *in vacuo*; pyridine and water (in a 3:1 ratio) were added, and the mixture was further heated for 3 h at 100 °C. The mixture was then evaporated to dryness, and the resulting solid was crystallized from ethanol, methanol, or ethyl acetate.

Physicochemical data with spectroscopic analyses for all the compounds are given in the Supplementary Information.

### Cell culture

The human colon carcinoma cell line HCT 116 with wild-type p53 (p53^+/+^) and the human glioblastoma cell line U-251 were obtained from ATCC, while the HCT 116 with a p53 deletion (p53^−/−^) was kindly provided by Prof. M. Rusin from the Maria Sklodowska-Curie Memorial Cancer Center and Institute of Oncology in Gliwice, Poland. The human chronic myelogenous leukaemia cell line K562 and the normal human cell dermal fibroblast cell line NHDF were purchased from Sigma-Aldrich and PromoCell, respectively. Both the HCT 116 and U-251 cell lines were cultured in Dulbecco’s modified Eagle’s medium (DMEM) supplemented with 10% inactivated foetal bovine serum (FBS). The DMEM for the NHDF contained 15% non-inactivated FBS. K562 cells were cultured in RPMI medium completed by 10% inactivated FBS. Additionally, the media were supplemented with antibiotics (streptomycin and penicillin – 1% v/v; Sigma-Aldrich). The cell culture was conducted in 75 cm^2^ flasks (Nunc) under standard conditions (37 °C, 5% CO_2_ humidified atmosphere). Furthermore, the cell lines were frequently tested for mycoplasma using the qRT-PCR technique with specific *Mycoplasma* primers to confirm the lack of contamination.

### Monolayer antiproliferative assay

The cells were seeded in 96-well plates (Nunc) with 5,000 or 4,000 cells per well for the cancer cells and NHDF, respectively, and incubated under standard conditions at 37 °C overnight. The culture media were then changed for freshly prepared solutions of the tested compounds in various concentrations. After 72-h incubation the antiproliferative assay was carried out as follows: the solutions of the compounds were changed in each well to 100 µL DMEM without phenol red and 20 µL of CellTiter 96 AQueous One Solution-MTS (Promega). The plates were incubated for 1 h at 37 °C, and the absorbance was then measured at 490 nm using a multi-plate reader (Synergy 4, BioTek). The experiment for each sample was repeated at least three times. The results are presented as the half-maximal inhibitory concentration (IC_50_) values, which were estimated in comparison to untreated cells (control) and calculated using GraphPad Prism v.8.0 (USA).

### 3D spheroid cell viability assay

The HCT 116 cell lines, AsPC-1, PANC-1 (5,000), and U-251 cells (8,000) in 100 µL of medium were seeded in 96-well ULA Corning spheroid microplates (Sigma-Aldrich) and incubated for 72 h at 37 °C to form spheroids. The medium from each well was then gently removed and freshly prepared solutions of **13** were added. The treated spheroids were incubated for the next 72-h. Afterward, cell viability assays were performed using CellTiter-Glo 3D Reagent (Promega) according to the manufacturer’s protocol. Briefly, CellTiter-Glo 3D Reagent was added in equal volume to the medium in each well. The plate was vigorously mixed for 5 min and then incubated for 25 min at room temperature. The luminescence was measured using a multiplate reader (Synergy 4, BioTek). The experiment for each sample was performed at least three times. The results from the assay are presented as IC_50_ values, which were calculated using GraphPad Prism v.8.0 (USA).

### Cell cycle assay

The cells were seeded in 35 mm Petri dishes, 200,000 cells per dish, and incubated overnight under standard conditions at 37 °C. The medium was changed, with freshly prepared solutions of **13** (twofold IC_50_ value) being added. After a 24-h incubation, the experiment was carried out using a Muse™ Cell Cycle Kit (Millipore) according to the available procedure. Briefly, the cells were detached and collected in Eppendorf tubes. The samples were then washed with cold phosphate-buffered saline (PBS), centrifuged at 300 g (5 min), and fixed in ice-cold 70% ethanol. The cells were stored at -20 °C overnight. The next day, the samples were sequentially washed with cold PBS, centrifuged resuspended in 200 µL of Muse™ Cell Cycle Reagent, and incubated in the dark for 30 min at room temperature. The percentage of cells in specified cell cycle phases was estimated using a Muse™ Cell Analyzer (Millipore). The experiments were carried out three times.

### Intercalation

Lyophilized calf-thymus DNA (CT-DNA; Sigma-Aldrich) was dissolved in 10 mM Tris-HCl (pH 7.9), mixed gently, and kept overnight at 4 °C. The purity of the CT-DNA solution was determined by measurement of the ratio of UV absorbance at 260 and 280 nm. A ratio value higher than 1.8, indicating that the DNA was sufficiently free from proteins. The tested compound was dissolved in DMSO to give a concentration of 8.35 mM. This stock solution was used for preparing new solutions (25, 12.5, 6, and 3 µM) in 1 mL of 10 mM Tris-HCl (pH 7.9). Next, 18 µM CT-DNA was added to the prepared solutions, and the samples were incubated for 1.5 h at 37 °C with occasional vortexing. The measurements of the absorption spectra were performed using a Hitachi U-2900 spectrophotometer in the range of 200–500 nm. The obtained data were analysed using GraphPad Prism v.8.0 (USA).

### Apoptosis induction assay

The cells were seeded in 35 mm Petri dishes (200,000 per dish) and incubated at 37 °C overnight. The following day, the medium was changed for a freshly prepared 0.5 µM solution of **13**. Analysis was carried out after 48 h using an FITC Annexin V Apoptosis Detection KIT with 7-AAD (Bio-Legend) according to the manufacturer’s protocol. In brief, both adherent and detached cells were collected, washed two times with cold PBS, and centrifuged at 300 g (5 min). Next, the cells were resuspended in 100 µL Annexin V Binding Buffer, 5 µL of FITC Annexin V, and 5 µL 7-AAD Viability Staining Solution. The samples were incubated in the dark for 15 min at room temperature. The percentage of live and apoptotic (sum of early and late apoptotic) cells was estimated using a Muse™ Cell Analyzer. The experiments were carried out three times.

### qRT-PCR

The cells were seeded in 35 mm Petri dishes at a density of 500,000 cells and incubated at 37 °C for 24 h. The total RNA was isolated after incubation with solutions of **13** (0.5 µM and 1.0 µM) for 24 h and 48 h, respectively. The isolation from the samples was carried out according to a TRIzol™ Reagent protocol (Thermo Fisher Scientific). Synthesis of cDNA was performed with 2 µg of total RNA using a GoScript™ Reverse Transcriptase kit (Promega) with Oligo(dT)_23_ Primers. Real-Time PCR was carried out using a CFX96 Touch™ Real-Time PCR Detection System (Bio-Rad) in 10 µL reaction volume. Each sample contained SsoAdvanced™ Universal SYBR Green Supermix (Bio Rad), a specific primer pair mix (0.5 µM each), and 1 µL of cDNA. The reaction was performed under the following conditions: initial denaturation at 95 °C for 30 s; 40 cycles of denaturation at 95 °C for 15 s; annealing (primer-specific temperature) for 30 s, and extension at 72 °C for 60 s. The obtained data were analysed based on a comparison of the expression of the target genes and normalized to a reference gene (GAPDH) using the Livak-Schmittgen (2^−ΔΔCT^) method. The experiments were performed at least three times. All of the primer pair sequences were purchased from Sigma-Aldrich and are listed in Table S1.

### Intracellular ROS level measurement

The cells (9,000 per well) were seeded in 96-well plates and treated with at freshly prepared 1 µM solution of **13** for 3, 6, 9, 12, and 24 h. The ROS levels were measured immediately according to the CellROX Green Reagent procedure. Additionally, the number of cells in each well during measurement was estimated using Hoechst 33,342 (Molecular Probes™). Briefly, after treatment the medium was removed and 100 mL mixture of CellROX Green Reagent (5 mM) and Hoechst 33,342 (6 mM) was added to each well. The plates were incubated for 30 min at 37 °C in the dark, and the fluorescence of samples was then measured using a multiplate reader (Synergy 4, Bio Tek). Readings were performed at 485 nm excitation and 520 nm emission, as well as at 345 nm excitation and 485 nm emission for the CellROX Green Reagent and Hoechst 33,342, respectively. ROS levels were calculated as the percentage relative to the untreated cell levels (control). The experiments were carried out three times.

### Western blot

The cells (500,000) were seeded in 35 mm Petri dishes (Nunc) and incubated in a solution of the tested compound (0.5 µM and 1 µM) for 24 h and 48 h. The cells were then dissociated using trypsin, collected in Eppendorf tubes, and centrifuged at 2,000 rpm. Next, cell pellets were resuspended in complete RIPA buffer containing a Halt Protease Inhibitor Cocktail and Halt Phosphatase Inhibitor Cocktail along with 0.5 M EDTA (all reagents from Thermo Fisher Scientific) and lysed on ice for 20 min. Afterward, the lysates were sonicated and centrifuged at 10,000 rpm for 10 min at 4 °C. The obtained supernatants were collected for further analysis. The protein concentration was determined using a Micro BCA™ Protein Assay Kit (Thermo Fisher Scientific) according to the manufacturer’s procedure. Equal amounts of proteins (16 µg) were electrophoresed on SDS-Page gels and transferred onto nitrocellulose membranes. Novex Sharp Pre-Stained Protein Standard (Thermo Fisher Scientific) was used as a colored molecular weight marker. The membranes were blocked in 5% non-fat milk prepared in TPBS (PBS supplemented by 0.1% Tween-20) for 1 h. After blocking, the membranes were cut, and then incubated with specific primary monoclonal antibodies (all purchased from Cell Signaling) overnight at 4 °C. The antibodies for the target proteins (CDK1 Mouse mAb #9116, p53 Mouse mAb #2524, FAS Mouse mAb #8023, GLUT-1 Rabbit mAb #12939, HO-1 Rabbit mAb #5853, p21^Waf1/Cip1^ Rabbit mAb #2947, PARP #9542) were diluted 1:1000, while the antibodies for the reference proteins (GAPDH Rabbit mAb #2118, β-actin Mouse mAb #3700, and vinculin Rabbit mAb #13901) were diluted 1:2000. The following day, the membranes were washed in TPBS and incubated with horseradish peroxidase (HRP)-conjugated secondary antibodies (Anti-rabbit IgG, HRP-linked Antibody #7074 or Anti-mouse IgG, HRP-linked Antibody #7076) for 1 h at room temperature. The membranes were then washed in TPBS and incubated with a SuperSignal™ West Pico Chemiluminescent Substrate (Thermo Fisher Scientific). Finally, the membranes were visualized using a ChemiDoc™ XRS + System (Bio-Rad). The experiments were carried out four to five times. The densitometric analysis was performed using ImageJ software (Wayne Rasband, National Institutes of Health, USA).

### Statistical analysis

The results are presented as the mean ± standard deviation (SD) from all the performed experiments. Depending on the sets of data, the statistical analysis was performed using an unpaired t-test with Welch’s correction or at one-way ANOVA with a Bonferroni post-hoc test. A p-value of 0.05 or less was considered to be statistically significant. All the statistical tests were performed using GraphPad Prism 8.0 software (GraphPad Software, USA).

## Results and discussion

### Chemistry

**Fig. 2:**
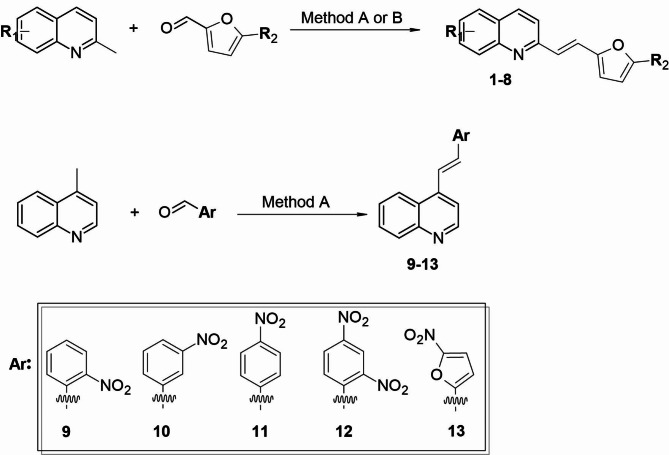
Synthesis of the studied compounds **1–13**. Method A: acetic anhydride : 80% acetic acid (3:1), 130 °C, 18–22 h; Method B: (i) acetic anhydride : 80% acetic acid (3:1), 130 °C, 18–22 h (ii) pyridine : water (3:1) 100 °C, 3 h.

The synthesis of the styrylquinolines and furanylvinylquinolines was performed according to known procedures, which we described previously^3,10,18^. All of the studied compounds were obtained by a condensation reaction of the corresponding quinoline derivative and aromatic aldehyde using conventional heating (Fig. 2). The precursors that were required to obtain the derivatives of styrylquinoline and furanylvinylquinoline were synthesised from commercially available reagents. The reactions were performed in a mixture of boiling acetic anhydride and 80% acetic acid in a ratio of 3:1 (method A). If the substrate in the reaction was quinaldine with a substituted hydroxyl group, the acylated form was obtained (compounds **4** and **8**), and then hydrolysis was performed in a pyridine/water mixture in a ratio of 3:1 (method B) to obtain the hydroxyl form (compounds **3** and **7**). All of the synthesised compounds were purified by crystallisation, and their structures were confirmed using ^1^H NMR, ^13^C NMR and MS (Supplementary Information (SI); Chaps. 1–2).

### Biological studies

#### Antiproliferative activity

The antiproliferative activity of the synthesised compounds was tested using an MTS assay against human colon carcinoma cell lines that had either a wild-type (HCT116 p53^+/+^) or p53 negative status (HCT116 p53^−/−^). Additionally, the compounds were also tested for their cytotoxicity against normal cells – human dermal fibroblasts (NHDF). The results of the antiproliferative activity assays are shown in Tables 1 and 2.

**Table 1:** Anticancer activity of the studied compounds **1–8** and reference Doxorubicin expressed as their IC_50_ values [µM]. ND – not determined, SI – selectivity index.

No.			IC_50_ [µM]		
	*R* ^1^	*R* ^2^	HCT 116 p53^+/+^	HCT 116 p53-/-	NHDF
1	8-OH	H	> 25	> 25	ND
2	8-OH	CH_3_	> 25	> 25	ND
3	8-OH	NO_2_	0.12 ± 0.02 (SI = 4.08)	0.18 ± 0.07 (SI = 2.72)	0.49 ± 0.07
4	8-OAc	NO_2_	0.20 ± 0.03 (SI = 3.35)	0.24 ± 0.05 (SI = 2.79)	0.67 ± 0.08
5	4-OH	NO_2_	0.30 ± 0.1 (SI = 1.50)	0.30 ± 0.07 (SI = 1.50)	0.45 ± 0.07
6	5,7-Cl-8-OH	H	4.01 ± 0.48 (SI > 6.23)	2.72 ± 1.09 (SI > 9.19)	> 25
7	5,7-Cl-8-OH	NO_2_	3.46 ± 0.34 (SI > 7.23)	1.33 ± 0.25 (SI > 18.80)	> 25
8	5,7-Cl-8-OAc	NO_2_	2.08 ± 0.40 (SI = 5.46)	1.12 ± 0.51 (SI = 10.13)	11.35 ± 2.53
**Doxorubicin**			0.34 ± 0.04 (SI = 0.41)	0.38 ± 0.03 (SI = 0.37)	0.14 ± 0.03

**Fig. 3:**
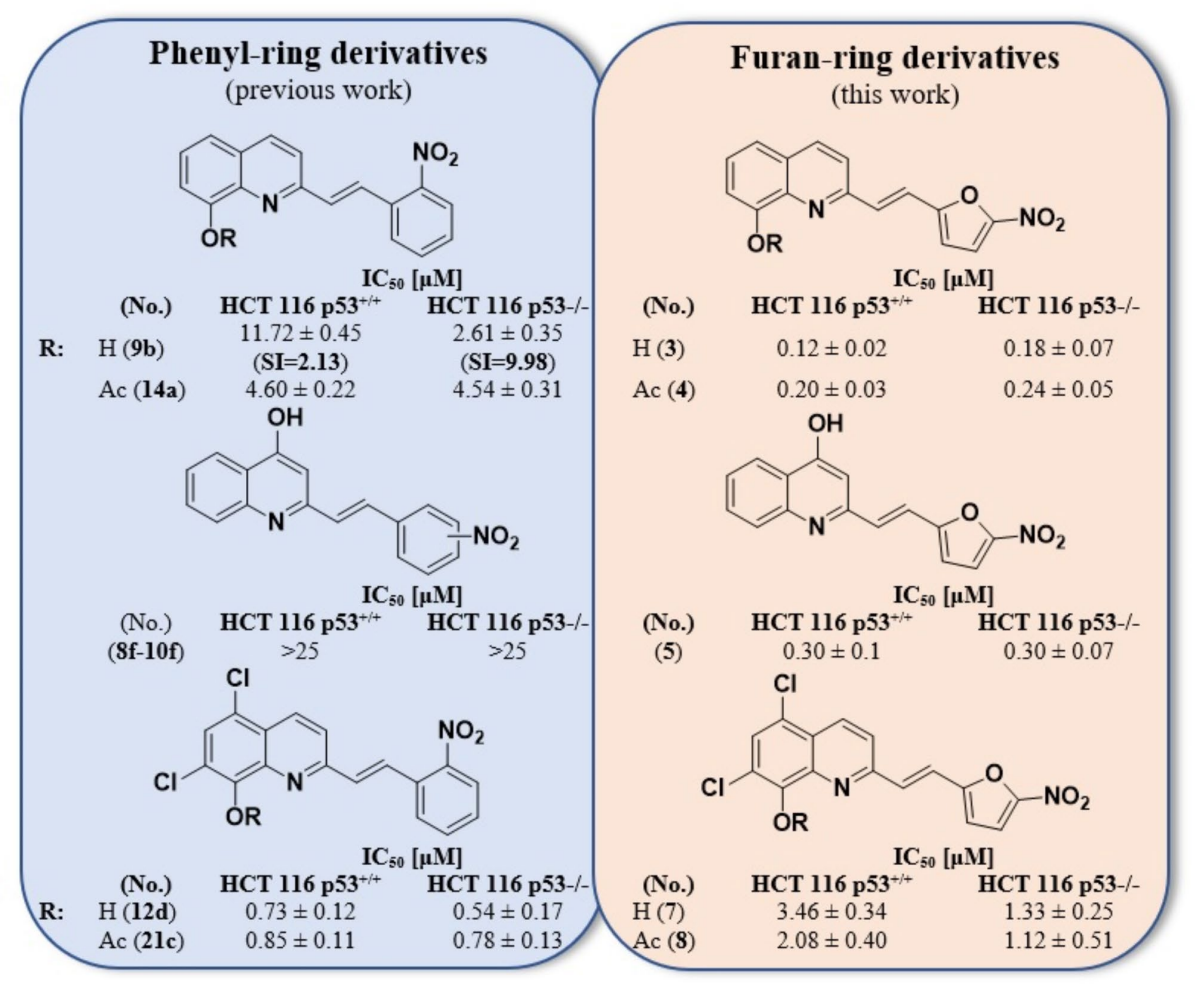
Comparison of the anticancer activity of the phenyl-ring derivatives (*from our previous study^3^) with the furan-ring derivatives.

Based on our previous studies^3^, and the cytotoxicity studies, it was concluded that both the replacement of phenyl with a furan ring in the 8-hydroxyquinoline derivative (**1**) as well as the further substitution of a methyl group onto the furan ring at position 5 (**2**) rendered these compounds inactive toward the tested cancer cells (IC_50_ > 25 µM). However, the substitution of the furan ring with a nitro group in the same position 5 (**3**) significantly affected the activity of the tested derivatives, the IC_50_ values against both HCT 116 cell lines being below 0.2 µM. Cytotoxicity studies for this compound had previously been performed, and our results were similar to those that had been obtained on human cancer cell lines of other origins^4,5^. Moreover, the activity of derivative **3** was significantly higher compared to its analog with a phenyl ring and NO_2_ group at position 2 (derivative **9b** [3]) for which the IC_50_ values for HCT 116 p53^+/+^ and HCT 116 p53^−/−^ were 11.72 µM and 2.61 µM, respectively (Fig. 3). Although the currently tested compound **3** was also cytotoxic against fibroblasts, it had a better selectivity index for HCT 116 p53^+/+^ than the previously tested compound with a phenyl ring. Acetylation of the hydroxyl group in the quinoline ring (**4**) did not improve the activity, while in the case of the styrylquinolines, acetylation of the 8-hydroxyl group (derivative **14a** [3]) improved the activity of the derivative against HCT 116 p53^+/+^ almost three-fold, although at the same time, it had a two-fold decreased activity against the HCT 116 p53^−/−^ cells compared to its hydroxyl analogue 9b. Such large differences in the activity for lines that differed in their p53 status prompted us to further study the mechanism of action of this group of compounds. Therefore, in this study, we extended the investigations to the lines in which p53 mutations or deletions occur in order to understand the role of this protein in the context of the action of these derivatives.

The previously tested and published 4-hydroxyquinoline derivatives (including those with a nitro-substituted phenyl ring) did not exhibit any anticancer properties. In contrast, the 4-hydroxyquinoline derivative with a 5-nitrofuran ring (**5**) that was tested here exhibited significant antiproliferative features against cancer cells. Therefore, in this case, the change to a heterocyclic ring with the analysed substituent also greatly improved the activity of that analogue. However, this compound was also the most toxic against fibroblasts (IC_50_ = 0.45 µM). On the other hand, adding the chlorine atoms to the 8-hydroxyquinoline ring at positions 5 and 7 resulted in a significant decrease in the IC_50_ values of **6** compared to **1** (from > 25 µM to 4 µM for HCT 116 p53^+/+^ and 2.7 µM for HCT 116 p53^−/−^), while still maintaining a selectivity against NHDF. Furthermore, the substitution of a nitro group onto the furan ring (**7**) increased the activity toward HCT 116 p53^−/−^ two-fold. Interestingly, the activity of the acetoxy analogue **8** of compound **7** did not change much against cells with a deletion of p53; in contrast, it increased against the cells with wild-type p53. In addition, differences in the IC_50_ values of the 5,7-dichloro derivatives were observed between the tested cancer cell lines. All three were twice as active toward HCT 116 p53^−/−^ than toward HCT 116 p53^+/+^. Preliminarily, these results suggested that the p53 protein was involved in the mechanism of action of these compounds. Furthermore, the derivatives of 5,7-dichloroquinoline **6–8** were considerably less active against cancer cells but had much better selectivity than their analogues without chlorine atoms (**3–5**). Moreover, it should be noted that in contrast to derivatives **3–5**, the replacement of the phenyl ring with a furan ring did not increase the activity of the 5,7-dichloro-8-hydroxyquinoline derivatives **7–8**.

After a series of encouraging results, we decided to substitute position 4 instead of 2 in the quinoline ring, thus obtaining 4-styrylquinolines. We did not find any reports in the literature on the anticancer activity for 4-styrylquinoline derivatives, so we decided to investigate this. In the styryl moiety, we placed a phenyl ring substituted with a nitro group at various positions (**9–12**). Additionally, we synthesised the 4-furanylvinylquinoline derivative with a furan substituent with a nitro group at position 5 (**13**). This compound was presented in paper^19^; however, it was tested for its antimicrobial properties, while its anticancer properties have not been previously described. After testing the cytotoxicity of these derivatives on the HCT 116 cells with the wild-type p53 protein and those with a deletion of this protein (Table 2), it was found that the IC_50_ values in the case of 4-styrylquinoline derivatives **9–12** were more than 25 µM.

**Table 2:** Anticancer activity of the studied compounds **9**–**12** and reference Doxorubicin expressed as IC_50_ values [µM]. ND – not determined.

No.		IC_50_ [µM]		
		HCT 116 p53^+/+^	HCT 116 p53-/-	NHDF
	*R*			
**9**	2-NO_2_	> 25	> 25	ND
**10**	3-NO_2_	> 25	> 25	ND
**11**	4-NO_2_	> 25	> 25	ND
**12**	2,4-NO_2_	> 25	> 25	ND
**Doxorubicin**		**0.34 ± 0.04**	**0.38 ± 0.03**	**0.14 ± 0.03**

However, the antiproliferative activity of derivative **13** with furan a ring was surprising. The IC_50_ values for the HCT 116 cells were below 0.1 µM; therefore, we extended the investigation to a broader panel of cancer cell lines that diverged in their p53 status (Table 3). For this purpose, we selected two pancreatic cancer cell lines: AsPC-1, which is characterised by a deletion of the p53 protein^20^ and PANC-1, which carries an R273H mutation^21^. This mutation also occurred in the glioblastoma U-251 cell line^22,23^. An additional non-adherent line that carries the *TP53* mutation that results in the absence of p53 is the K562 leukemic cell line^24^. Further studies confirmed the high activity of derivative **13**, for which the IC_50_ values were maintained at a level below 0.1 µM for glioblastoma (U-251) and leukaemia (K562) and did not exceed 0.2 µM for the pancreatic cell lines. Moreover, **13** was selective toward fibroblasts, as indicated by its SI values (3.35–9.41). Among all of the tested cell lines, the highest activity of the compound was found against the HCT 116 cells with the wild-type p53 protein (0.069 µM). However, in the case of both HCT 116 cell lines, **13** was more than four times more active than the commercially available drug, doxorubicin, while at the same time it was also more than four-fold less toxic against fibroblasts. The p53 protein also interacts with the metabolic pathways that are associated with glucose transport^25^ as well as small molecules^26^. In light of the above-mentioned facts, we also determined the cytotoxicity of the GLUT inhibitor BAY-876, which is based on a quinoline scaffold. BAY-876 was the most active compound in the PANC-1 cells with an R273H mutation of p53. Interestingly, for the cell line with the same mutation (U-251), it was not active at 25 µM. In HCT 116 p53^+/+^, the IC_50_ value was 1.71 µM, while for the same line with a deletion of the *TP53* gene, the activity of this inhibitor decreased more than six-fold. Furthermore, BAY-876 was also toxic to normal cells.

**Table 3:** Anticancer activity of derivative **13** and references (Doxorubicin and BAY-876) expressed as the IC_50_ values [µM]. ND – not determined, SI – selectivity index. The last row shows the results that were obtained for derivative **13** on a 3D model.

No.	IC_50_ [µM]						
	HCT 116 p53^+/+^	HCT 116 p53-/-	U-251	K562	AsPC-1	PANC-1	NHDF
**13 (2D model)**	0.069 ± 0.009 (SI = 9.41)	0.094 ± 0.010 (SI = 6.90)	0.087 ± 0.007 (SI = 7.46)	0.093 ± 0.009 (SI = 6.98)	0.123 ± 0.007 (SI = 5.28)	0.193 ± 0.008 (SI = 3.36)	0.649 ± 0.060
**13 (3D model)**	1.16 ± 0.19	1.00 ± 0.13	0.34 ± 0.07	ND	4.18 ± 1.00	2.16 ± 0.37	ND
**Doxorubicin (2D model)**	0.34 ± 0.04 (SI = 0.41)	0.38 ± 0.03 (SI = 0.37)	0.05 ± 0.02 (SI = 2.80)	0.02 ± 0.01 (SI = 7.00)	0.86 ± 0.13 (SI = 0.16)	0.73 ± 0.09 (SI = 0.19)	0.14 ± 0.03
**BAY-876 (2D model)**	1.71 ± 0.27 (SI = 2.53)	10.79 ± 0.81 (SI = 0.40)	> 25 (SI < 0.17)	ND	0.16 ± 0.01 (SI = 27.06)	0.035 ± 0.004 (SI = 123.71)	4.33 ± 0.48

To confirm the cytotoxicity ranking, the viability of the cells was also estimated on a 3D spheroid model, which better represented a solid tumour. In contrast to the results that were obtained with the monolayer model, the tested derivative was the most active against the U-251 spheroids (IC_50_ = 0.34 µM). However, derivative **13** was also characterised by a high activity against the colon cancers spheroids for which the IC_50_ values were approximately 1 µM. The cell lines that represented pancreatic cancer were less responsive to derivative **13**, which was similar to the 2D model. It is well known that pancreatic cancer exhibits a high drug resistance^27,28^. However, the activity of derivative **13** was confirmed on the 3D model that reflected a real tumour, which is undoubtedly a positive result and a reason for further, more in-depth studies to clarify the mechanism of action of the 4-furanylvinylquinoline derivative **13**.

### Cell cycle inhibition

**Fig. 4:**
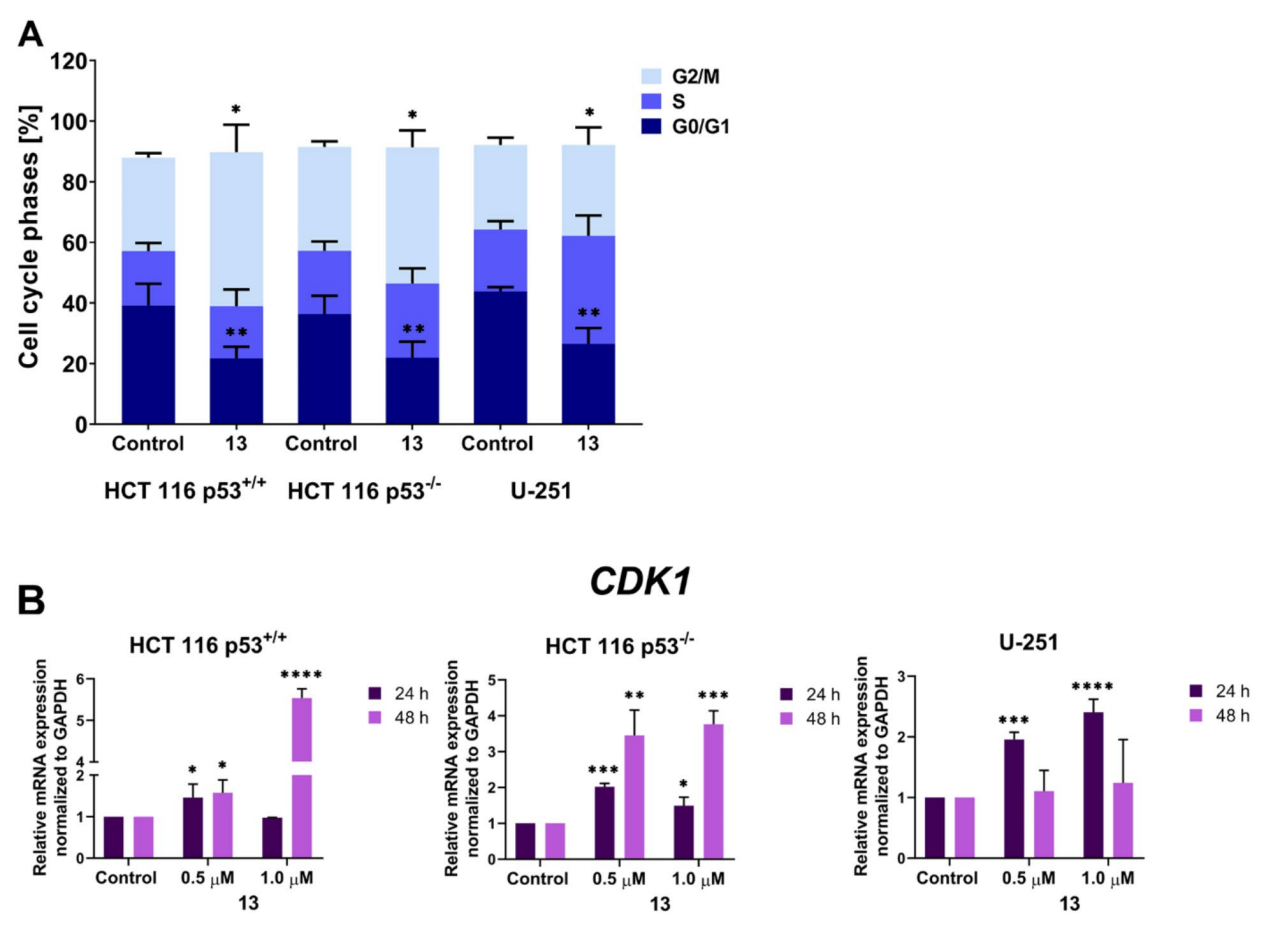
**(A)** The influence of derivative **13** (concentration 2xIC_50_) on the cell cycle progression measured by flow cytometry in the HCT 116 p53^+/+^, HCT 116 p53^−/−^, and U-251 cell lines after 24-h incubation. The results are presented as the mean ± SD and summarised in the chart. The data were analysed using an unpaired t-test with Welch’s correction: *p < 0.05, **p < 0.01, ***p < 0.001, ****p < 0.0001 compared to the untreated cells (control). **(B)** The charts present the relative mRNA expression of *CDK1* in the tested cell lines after 24- and 48-h treatment with derivative **13** at various concentrations. The statistical analysis was performed using a one-way ANOVA with Bonferroni’s post-hoc test: *p < 0.05, **p < 0.01, ***p < 0.001, ****p < 0.0001 compared to the untreated cells (control).

When analysing the antiproliferative activity of the tested compounds, we decided to determine the molecular mechanism of action of the most promising derivative **13** in three cell lines with different statuses of p53, for which the derivative has the highest activity. In order to achieve this goal, we started by studying the changes in the cell cycle in order to determine why proliferation stops. To evaluate the effect of derivative **13** on the cell cycle progression, we used flow cytometry. After a 24-h treatment, we observed a significant inhibition in the G2/M phase in both HCT 116 cell lines (Fig. 4A). The population of cells in this phase increased from 30.81 to 49.78% and from 34.35 to 44.95% in HCT 116 p53^+/+^ and HCT 116 p53^−/−^ cells compared to control cells, respectively. On the other hand, in the case of the U-251 cells, the compound inhibited the cell cycle in the S phase. There was a pronounced increase in the percentage of cells from 20.44 to 35.59%. The representative histograms from the flow cytometry are shown in Fig. S1 in SI.

In the next step, we decided to investigate the impact of derivative **13** on the mRNA level of *CDK1* (the gene encoding one of the cell cycle regulation proteins) at various concentrations and time points (Fig. 4B). In the HCT 116 p53^+/+^ cells after the application 0.5 µM of derivative **13**, we noticed a slight increase in the expression of *CDK1* after both 24 and 48-h. Interestingly, after the 24-h treatment with 1 µM, we did not observe any significant changes relative to the untreated cell, but after 48-h of incubation, the mRNA level rapidly increased. In contrast, after 24 h, the level of *CDK1* was two-fold higher in the HCT 116 cells with a deletion of *TP53* than in the control cells at a concentration of 0.5 µM and around one and the half times in the 1 µM samples, while being considerably increased after 48 h. We also observed a greater than two-fold upregulation of the *CDK1* expression in the U-251 cells after 24 h. However, after a further incubation time, the mRNA level of this gene was similar to that in the untreated cells.

To determine whether and to what extent the compound interacts with DNA, we also performed a spectrophotometric study on CT-DNA. We observed a moderate hypochromism of derivative **13** (10.97%). (Fig. S2). Nevertheless, under the effect of stress-induced DNA damage in cells, CDC25C, as well as p53, were activated, which inhibited the CDK1 and cyclin B1 complex. This had the effect of arresting the cell cycle in the G2/M phase in an attempt to repair the DNA damages or induce apoptosis^29,30^. Abnormal CDK1 activation is often thought to be connected with tumourigenesis. However, in recent years, there have been reports that CDK1 is also involved in the induction of cell death *via* apoptosis^31,32^.

### Apoptosis induction

**Fig. 5:**
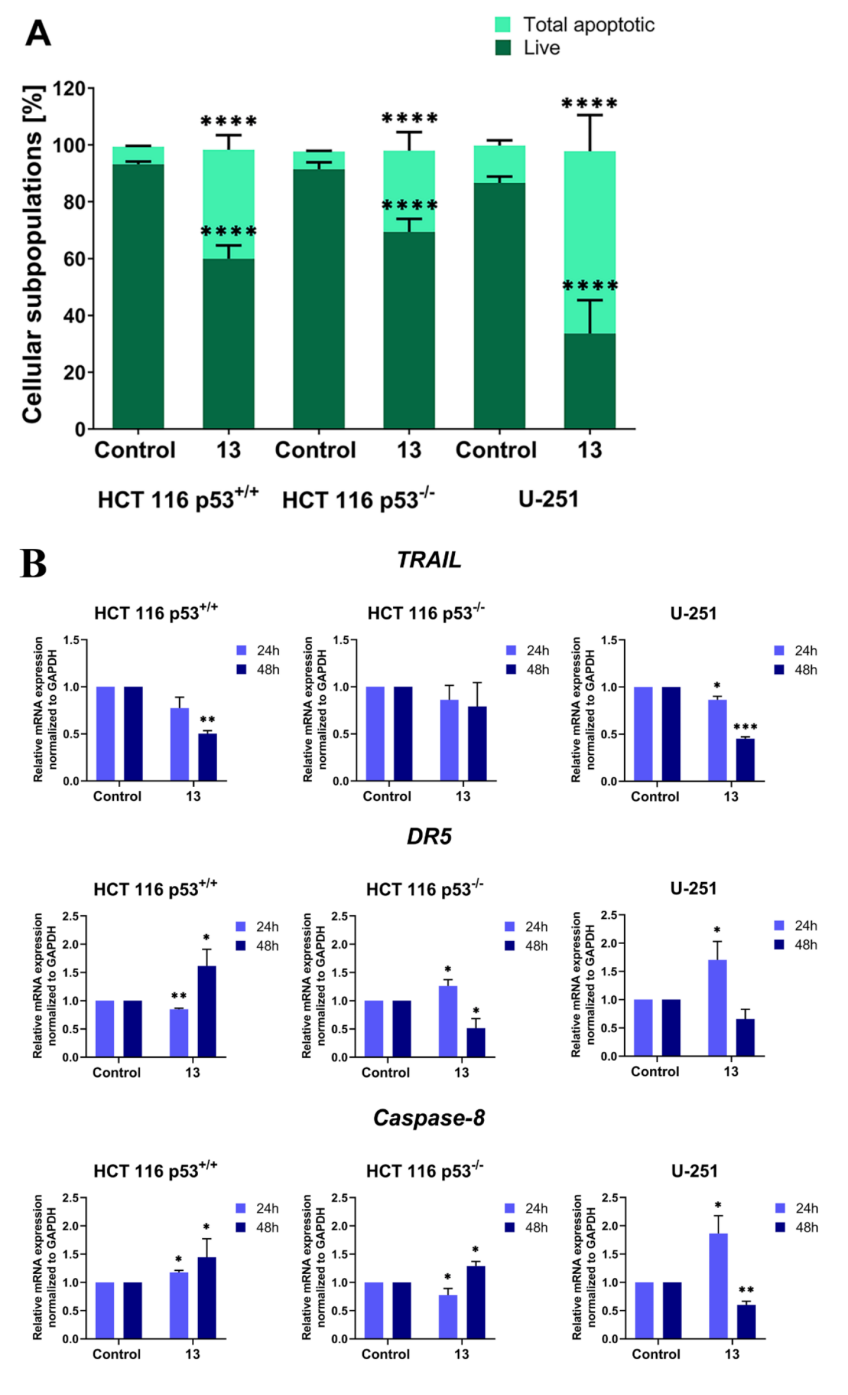
**(A)** The effect of derivative **13** (concentration 0.5 µM) on apoptosis induction in the HCT 116 p53^+/+^, HCT 116 p53^−/−^, and U-251 cell lines after a 48-h incubation. The percentage of live, apoptotic (early and late), and dead cells from all of the performed experiments for each of the cell lines are shown in the chart as the mean ± SD. A statistical analysis of data was performed using an unpaired t-test with Welch’s correction: *p < 0.05, **p < 0.01, ***p < 0.001, ****p < 0.0001 compared to the untreated cells (control). **(B)** The charts present the relative mRNA expression of *TRAIL*,* DR5*, and *caspase-8* in the three tested cell lines after treatment with derivative **13.** Statistical analysis was performed using a one-way ANOVA with Bonferroni’s post-hoc test: *p < 0.05, **p < 0.01, ***p < 0.001, ****p < 0.0001 compared to the untreated cells (control).

Because derivative **13** affected the cell cycle progression, we decided to determine the type of induced cell death by focusing on the apoptosis pathway. The analysis was performed by measuring the green fluorescence of the annexin V – FITC dye conjugate using capillary flow cytometry. Positive staining is observed when the annexin V binds to the disintegrated membrane of apoptotic cells. Generally, after treatment with derivative **13**, there was a strong increase in the percentage of apoptotic cells in all of the cell lines that were tested, namely from nearly 6–40% and 30% for HCT 116 p53^+/+^ and HCT 116 p53^−/−^ cells, respectively (Fig. 5A). However, the highest percentage of apoptotic cells was observed in the U-251 cells, where their fraction changed from approximately 13% in the control to 66% in the treated cells. The representative histograms from the flow cytometry are shown in **Figure **S3 in SI.

After analysing the obtained results, we hypothesised that the compound affects p53, and therefore we decided to investigate some of the targets that are associated with extrinsic apoptosis. Because certain chemotherapeutic agents can induce the tumour necrosis factor (TNF), which is related to apoptosis-inducing ligand (TRAIL)-mediated apoptosis^33^, we decided to investigate the effect of derivative **13** on the expression of *TRAIL*, *TRAIL receptor 2* (*TRAILR2/DR5*), and the extrinsic pathway related to *caspase 8* (Fig. 5B). In all of the cases, there was a decrease in *TRAIL* mRNA level; however, it was not significant in the HCT 116 p53^−/−^ cells. Certain cytostatic compounds, such as etoposide, which induce apoptosis by activating caspase 8, did not induce an mRNA expression of the death receptor ligands^34^. The compound that was tested here clearly increased the expression of *DR5* after just a 24-h treatment in the U-251 cells, and an increase was also observed in HCT 116 p53^−/−^ after that time, but to a lesser extent. In contrast, in the colorectal cancer cells with the wild-type p53, there was an increase in the expression of the gene encoding this receptor after 48 h.

According to the results that were obtained by Wachter et al., both the wild-type and mutated p53 in cancer cells play important roles in the induction of the extrinsic apoptosis pathway^35^. Our research also confirmed this correlation. However, these authors emphasised that the p53 status had no significant effect on the changes in the expression of apoptotic targets that are regulated by p53, including *DR5.* In the results presented here, after incubation with derivative **13**, we observed different levels of increase in the *DR5* expression depending on the cell line. P53-mediated apoptosis *via* an enhancement of the expression of *DR5* was observed in HCT 116 with the wild-type p53 after treatment with 5-fluorouracil^36^ as well as with prostaglandin A_2_ (PGA_2_)^37^. Moreover, we observed a greater sensitivity to the induction of apoptosis in HCT 116 p53^+/+^ than in HCT 116 p53^−/−^. Similar observations were recently made by Willms and colleagues after treatment with clinically tested agonistic antibodies against the TRAIL receptors, which indicates the important role of p53^38^. Nevertheless, our results showed the highest sensitivity to the induction of apoptosis in the p53-mutant cells, which was also confirmed by the high expression level of *DR5* after 24 h. After this time, we also observed a significant up-regulation of *caspase-8*, which is one of the major molecular targets in executing the extrinsic apoptotic pathway in U-251 cells^39^. A less pronounced change in the mRNA level of this gene was observed in both HCT 116 cell lines. This might be related to a recent report that showed that cancer cells can use caspase 8 to overcome the checkpoint between the G2/M phases of the cell cycle. The results presented above indicated that cell cycle arrest occurred in this phase in HCT 116 cells. Interestingly, p53 might also act as a key regulator of caspase-8 expression^40^. Liu and colleagues reported that an increase in the mRNA level expression of caspase-8 was not observed after treatment with a cytostatic compound in cancer cells that lacked p53. We made a similar observation in the p53-deficient HCT 116 cells. Our results also confirmed that the tested derivative induced the expression in the selected targets of extrinsic apoptosis based on the p53 status in cells. Furthermore, it has also been reported that a higher level of *DR5* is not only associated with p53 activation, but also with the DNA damage that is caused by ROS^41^. Therefore, in the next step, we decided to determine the impact of derivative **13** on oxidative stress.

### Oxidative stress induction

**Fig. 6:**
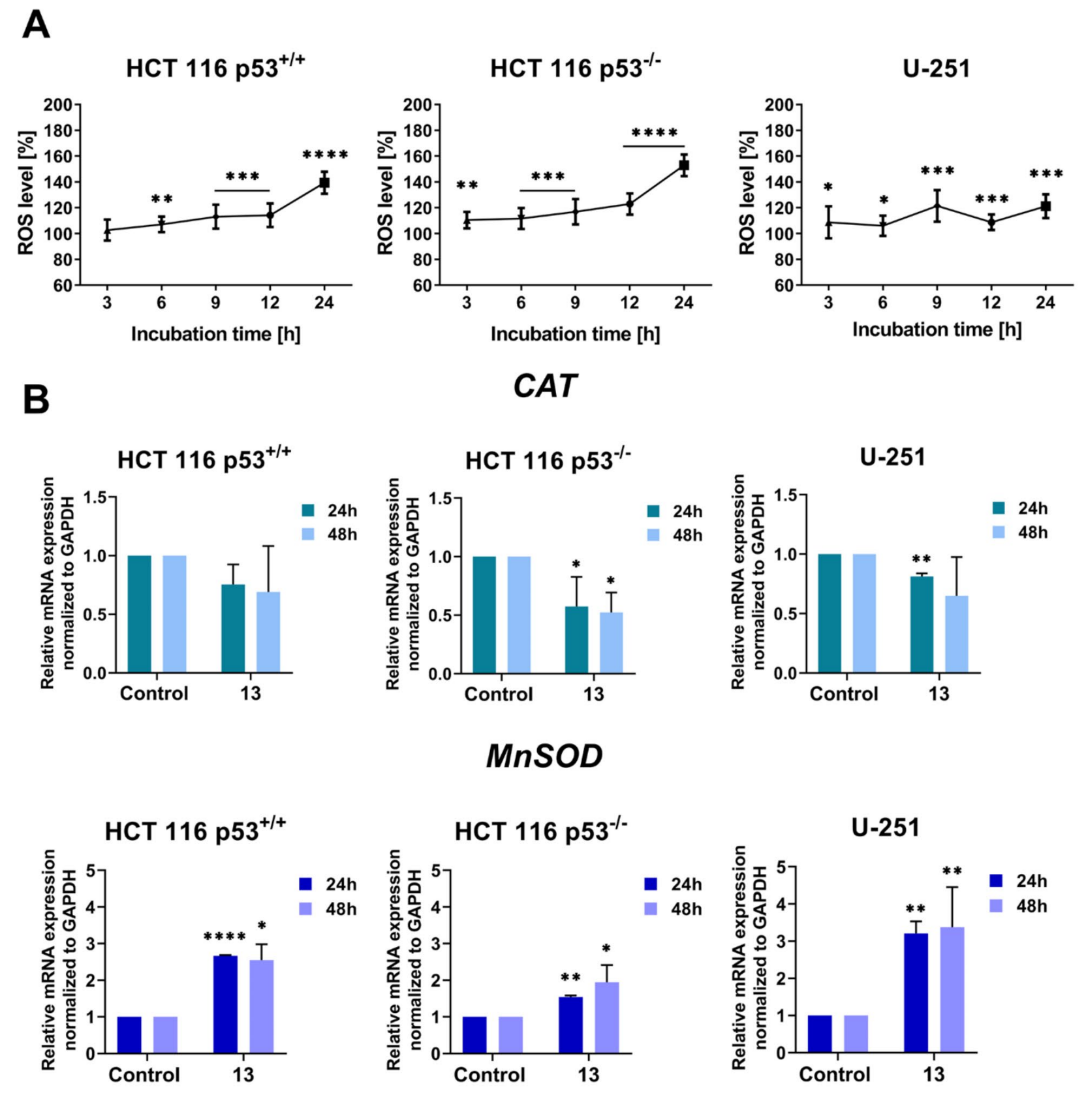
The impact of derivative **13** on the induction of oxidative stress in the HCT 116 p53^+/+^, HCT 116 p53^−/−^, and U-251 cell lines. **(A)** The line charts present the effect of the tested compound on the generation of ROS. The results are presented as the mean ± SD of three independent experiments. The data were normalised to the untreated cells (control) and analysed using the unpaired t-test with Welch’s correction: *p < 0.05, **p < 0.01, ***p < 0.001, ****p < 0.0001. **(B)** The column charts show the changes in the relative mRNA expression (presented as the mean ± SD) of *CAT* and *MnSOD* after 24- and 48-h treatment with derivative **13**. The results from three independent experiments are presented. The statistical analysis was performed using a one-way ANOVA with Bonferroni’s post-hoc test: *p < 0.05, **p < 0.01, ***p < 0.001, ****p < 0.0001 compared to the control.

To determine the ROS level, the fluorescent CellROX Green Reagent was used, being measured by a spectroscopic multi-plate reader. A kinetic increase in the ROS level was observed in each of the tested lines within 24 h (Fig. 6A). The most noticeable increase was observed in HCT 116 with a p53 deletion. The ROS level was several percent higher compared to the control after only 3 h. After 24 h the amount of ROS increased by as much as 50% relative to the untreated cells. Similar observations were made in the case of the HCT 116 p53^+/+^ cells. However, the generation of ROS was less pronounced, especially at the first time points. Nevertheless, after 24 h, the level of ROS was about 40% higher. The least visible increase in ROS was observed in the U-251 cells. The highest (20% growth) occurred after 9 h and 24 h. Interestingly, after a 12-h incubation, there was a slight decrease. The intracellular ROS levels are rapidly regulated by activated p53, which initially decreases and then subsequently increases them^42^. The weaker response toward ROS generation in these cells might have been caused by the fact that U-251 cells are resistant to exposure to oxidative stress. Liu et al. demonstrated that a high concentration (300 µM) of hydrogen peroxide (H_2_O_2_) inhibited the proliferation of U-251 cells after 24 h^43^. Additionally, cancer cells with a p53 mutation have a higher level of ROS than those with the wild type. Some p53 mutants can gain oncogenic features that are described as “gain-of-function” (GOF), among them being U-251 cells^22^. By controlling the signalling pathways that are associated with the redox balance, the GOF p53 mutants promote an elevated basal level of ROS in cancer cells^44,45^. Therefore, in the case of the tested glioblastoma cells, derivative **13** had a smaller effect on ROS generation than it did in the colon carcinoma cells.

The overproduction of ROS might involve the induction of apoptosis^3^ and might also have an impact on the regulation of the expression of some of the enzymes that are involved in maintaining the appropriate redox balance. Among the most important ones are manganese superoxide dismutase (MnSOD) and catalase (CAT), which were examined by qRT-PCR. MnSOD is one of the antioxidant regulators that are located in the mitochondrial matrix, which maintain the appropriate level of ROS by converting superoxide anion (O_2_.^−^) into hydrogen peroxide (H_2_O_2_), which is then dismutated into water and oxygen by CAT in the cytoplasm^46,47^. After treatment with derivative **13**, we observed an increase in *MnSOD* expression at both time points and in all of the tested cell lines (Fig. 6B). These results confirmed that the antioxidant system was activated in the cells due to disturbances in ROS homeostasis. However, the mRNA level of *MnSOD* was particularly marked in the cells with p53 (HCT 116 p53^+/+^ and U-251). As was reported by Zhao et al., a compound that activates p53 might also induce the translocation of this protein into the mitochondrial matrix where it could then interact with MnSOD^48^. The regulation of the MnSOD expression by p53 can proceed in two ways. Some studies have shown that p53 inhibits the expression of this antioxidant enzyme^49^. On the other hand, other results have shown that p53 might enhance the expression of MnSOD through an interaction with the other transcription factors that are associated with cell proliferation, such as NF-*κ*B^50^. The overexpression of MnSOD by the activation of p53 was observed in human lymphoblasts^51^. It caused an increase in oxidative stress, which caused apoptosis. In contrast to *MnSOD*, the *CAT* levels were lower than in controls. Given that the levels of these two genes were recorded at the same time points, the results demonstrate that the antioxidant system of the cells was activated. Because the stronger radicals (such as HO. or O_2_.^−^) are inactivated first, followed by the weaker ones, such as H_2_O_2_, the expression of the ROS scavengers may vary over time. The results obtained in our study confirm the hypothesis that the activation of p53 might upregulate the gene expression of *MnSOD* because in HCT 116 p53^−/−^, the *MnSOD* expression was much less noticeable than in the case of the other lines with p53. In our research, we also observed an increase in *MnSOD* and apoptotic cell death. Derivative **13** induced oxidative stress *via* the increased production of intracellular ROS and affected the activation of the antioxidant system.

### Protein level analysis

**Fig. 7:**
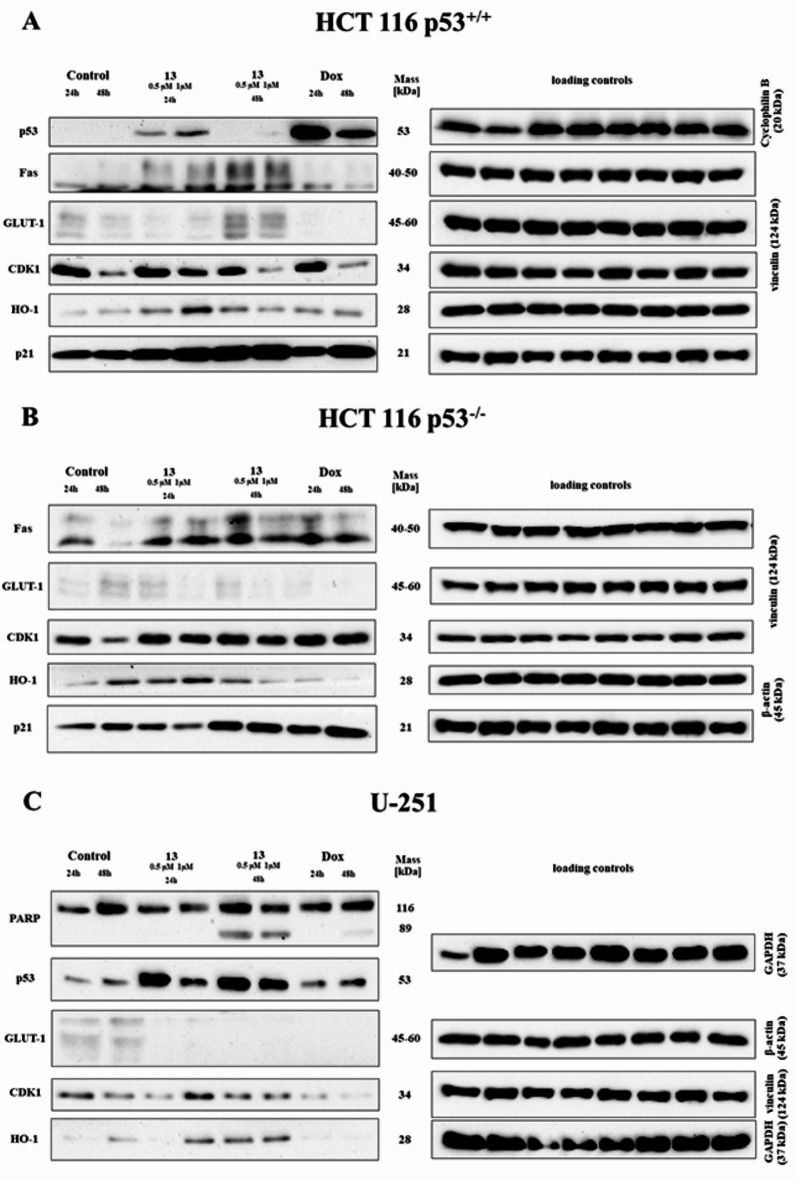
The effect of a 24- and 48-h treatment with derivative **13** (0.5 and 1 µM) and DOX (5 µM) on the expression of the selected cell cycle, apoptosis, and oxidative stress-related proteins in the HCT 116 p53^+/+^ (**A**), HCT 116 p53^−/−^ (**B**), and U-251 (**C**) cell lines. The Figure contains cropped blots, while the original blots/gels are presented in Supplementary Information (Chap. 3.5. Original uncropped membranes).

To determine the effect of derivative **13** on the cellular pathways and protein expression, Western Blot analysis was performed. The activation of the p53 protein was observed after a 24-h incubation in cells with the wild type (HCT 116 p53^+/+^) (Fig. 7A) and mutation (U-251) at both time points (Fig. 7C). In the glioma cells, higher relative levels of p53 were observed after 48-h. As might be expected, in the HCT 116 p53^−/−^ cell line, we did not observe its occurrence due to the deletion of the gene encoding this protein. The first important conclusion of the analysis is undoubtedly the involvement of the p53 protein in the mechanism of action of derivative **13**. The HIF-1α protein was also analysed, but its activation was not found in any of the tested cell lines (data not shown). Therefore, the involvement of hypoxia in the mechanism that affected the antiproliferative activity of derivative **13** was excluded.

The results obtained from the analysis of the generation of reactive oxygen species led us to analyse the level of the HO-1 protein – an enzyme that is responsible for maintaining normal cellular homeostasis, and that also plays an important role in the response to oxidative stress. A concentration-dependent increase of HO-1 was observed in all of the cell lines (Fig. 7). The highest level was observed at 1 µM after 24 h (more than twelve-fold in HCT 116 p53^+/+^, six-fold in HCT 116 p53^−/−^, and four-fold in U-251) (Fig. S4). Interestingly, an increase in HO-1 after treatment with doxorubicin was only observed in the HCT 116 p53^+/+^ cells, whereas in the other two lines, there was a decrease in this enzyme. Furthermore, a decrease in HO-1 relative to the control was observed in the HCT116 cells with p53 deletion after 48 h in contrast to the wild-type cells, where the levels were still higher than in the control. Derivative **13** induced oxidative stress *via* the increased production of intracellular ROS, which affected the activation of the antioxidant system. However, the redox disturbances were so strong that the activation of the scavengers could not cope with the excessive stress, and the cells switched to the cell death pathway, more specifically apoptosis. Recent studies on human osteosarcoma U2OS cells have also shown that HO-1 induction was associated with cell cycle arrest in the G2/M phase^52^. The results presented in this paper also showed significant cell cycle inhibition in this phase in the cells of both of the studied colorectal cancer lines. Therefore, the increased activation of HO-1 in the HCT 116 p53^+/+^ and p53^−/−^ cells might have been due to its involvement in the inhibition of cell cycle progression. The U-251 line exhibited the lowest relative increase in the HO-1 levels compared to the control cells.

In the present study, at both concentrations, derivative **13** caused a more than two-fold increase in the p21 protein expression in the HCT 116 p53^+/+^ cell line, which persisted after 24 and 48-h. In the case of the HCT 116 p53^−/−^ cells, a concentration of 0.5 µM stimulated the activation of this protein after a 24-h incubation, while a concentration of 1 µM had an inhibitory effect on the p21 expression. These results indicate that the p21 protein activation was independent of the p53 protein, as described in^53^. The overexpression of p21 also causes cell cycle inhibition *via* its binding to the CDK1/cyclin E complex^54^. In addition, it has been proven that the inhibition of the cell cycle in the G2/M phase that is caused by CP-31,398 (a styrylquinazoline) was associated with p21 activation^55^. In the tested colorectal cancer cell lines, derivative **13** clearly induced cell cycle arrest precisely at this phase, and therefore the higher levels of p21 were most likely due to its involvement in cell cycle regulation rather than apoptosis induction. The p21 protein does not accumulate when the cycle is inhibited in the S-phase, even when cells are treated with DNA-damaging agents that cause it to accumulate to a degree that is easily detectable by the Western Blot method^56^. The cell cycle in the U-251 cell line was inhibited precisely in the S-phase, and it is, therefore, likely that the p21 protein expression was not detected in the glioma cells tested (data not shown).

The results that were obtained for the CDK1 protein overlapped with those for the cell cycle analysis using flow cytometry (Fig. 4A) and expression analysis of the gene encoding this kinase (Fig. 4B). In the HCT 116 p53^+/+^ line cells, a slight increase in the expression of this protein was generally observed at both 24-h and 48-h after the treatment with derivative **13** (Fig. 7A). The level of the transcribed protein was quite similar to the *CDK1* gene expression. However, there was a lack of this correlation after 48-h with 1µM of the tested compound. The *CDK1* gene expression at this concentration increased sharply, while the protein levels decreased significantly relative to the untreated cells. The most likely explanation for this could be the activation of various metabolic processes in order to protect the cells from the oxidative stress that was induced by the administration of the compound. Therefore, there was such a sharp increase in the expression of the gene encoding this kinase that as many cells as possible managed to undergo mitotic division. However, a full translation did not occur through the damage to the mechanism of protein synthesis, for example at the level of ribosomes, which in turn is the result of excessive levels of ROS^57^. This resulted in a decrease in the CDK1 levels and cell cycle arrest in the G2/M phase. On the other hand, after a 24-h incubation with derivative **13** at both tested concentrations, the level of the CDK1 protein in the HCT 116 p53^−/−^ cell line was quite similar to that in the control cells. After 48-h, an intense activation of CDK1 was detected, which in turn coincided with the results that were obtained at the mRNA level. The observed phenomenon was probably related to the downward trend in the p21 protein at this time, which had an inhibitory effect on CDK1 kinase. Interestingly, the sharp increase in CDK1 levels in this cell line after 48-h was also observed after the doxorubicin treatment. In the U-251 glioma cells, there were no significant differences in the protein levels between the untreated cells and those that were incubated with derivative **13**. The only significant accumulation of this protein occurred after 48-h at the higher tested concentration. These results suggest that the inhibition of the cycle occurred in the S phase.

The results of the analysis of induced cell death prompted us to determine the levels of the proteins that are associated with apoptosis induction. The tested compound affected the activation of Fas protein in the colorectal cancer cells. In the HCT 116 p53^+/+^ cell line, a more than three-fold increase in the level of this marker of the extrinsic apoptosis pathway was observed after 24-h (Fig. S4A). The intensity of the Fas expression in this cell line was also maintained after 48-h, but to a lesser extent. In contrast, in the HCT 116 p53^−/−^ cells, the increase in the Fas protein expression occurred after 48-h and was four times higher than in the untreated cells (Fig. S4B). In the case of both of the tested colon cancer lines, there was no significant increase in the level of this protein after the doxorubicin treatment. The obtained results corroborated those from the mRNA expression studies on the other selected markers of the extrinsic apoptosis pathway, which indicated the existence of this cell death pathway in both of the studied HCT 116 lines. The difference in the activation time could have been related to the presence of the p53 protein in the cells. There are reports on the regulation of the apoptosis pathway in which Fas participates depending on its p53 status, which only involves Fas-specific binding to the wild-type p53 protein^58^. It is also known that the transcription of the gene encoding Fas can be regulated by the nuclear factor NF-κB^59^, Wig-1^60^, or p73^61^, which could be related to the later activation of this protein in the HCT 116 p53^−/−^ cells. On the other hand, it has been proven that Fas is not always expressed in cells that express the wild-type p53, instead it was detectable in cells with a homozygous *TP53* deletion^62^. Given the above information, it was not possible to indicate to what extent the p53 protein regulated the Fas expression. Nevertheless, derivative **13** undoubtedly affected the upregulation of this membrane protein. Interestingly, the Fas protein was not detected in the U-251 cells by the Western Blot method (not shown). In this case, it cannot be excluded that mutated p53 played an important role in its repression. Notably, Zalcenstein et al. showed that the mutant p53 protein inhibited the expression of the gene encoding the Fas receptor protein, and the degree of this inhibition varied depending on the type of the p53 mutation^63^.

In contrast to both of the HCT 116 lines, proteolysis of the PARP-1 protein was observed in the glioma cells after 48-h. The level of the 89 kDa product, which confirmed apoptosis, after treatment with the tested derivative **13**, was higher than after the doxorubicin treatment (Fig. 7C). By analysing the previous results that prove the induction of apoptosis, PARP-1 could also be seen as marker of the DNA damage that affected the topoisomerases, which are enzymes that are essential for DNA replication, as described for glioma cell lines including U-251^64^. It is, therefore, possible that the molecular substrate in the S-phase cell cycle inhibition after treatment with derivative **13** was precisely the interaction of the compound with the topoisomerases.

Considering the obtained results (the influence of the studied derivative on p53 activation) and the fact that wild-type p53 positively regulates oxidative phosphorylation and inhibits glucose metabolism through the inhibition of glucose transporters, including GLUT-1 and GLUT-4, among others, we decided to perform further analysis. In turn, a p53 mutation increased the expression of these glucose transporters, which favours aerobic glycolysis over oxidative phosphorylation, i.e., p53 mutations contribute to the Warburg effect.

We, therefore, decided to test the effect of the tested compound on the level of the main glucose transporter, GLUT-1. In the HCT 116 p53^+/+^ cells, a 24-h incubation with derivative **13** resulted in a decrease in the level of this glucose transporter, whereas a rapid activation of this protein was observed after 48-h. The results obtained for this cell line showed the abovementioned mechanism of the regulation of the glucose transporter activity by the wild-type p53 protein. Indeed, after 24-h the activation of this protein was observed in these cells, which coincided with the inhibition of GLUT-1. In contrast, after 48-h, no activation of p53 was observed, which resulted in a sharp increase in the expression of the glucose transporter for cell defence. The validity of the described conclusions was confirmed by the results that were obtained for doxorubicin. in which p53 activation occurred at both tested time points with a concomitant decrease in the GLUT-1 levels. On the other hand, despite the slight increase in the GLUT-1 level after a day of incubation with 0.5 µM of derivative **13**, a decrease in the level of this transporter was observed in the HCT 116 p53^−/−^ cells, which would indicate a p53-independent GLUT-1 inhibition. Notably, in the U-251 glioma cells, the compound caused such a significant decrease in the level of this protein compared to that untreated cells that it was not detectable by the Western Blot method. The results that were obtained from the measurements of the expression of this protein were so interesting, and at the same time, inconclusive that we additionally decided to determine the effect of the tested derivative **13** on the expression of the genes encoding selected glucose transporters.

### Expression of glucose transporter genes

**Fig. 8:**
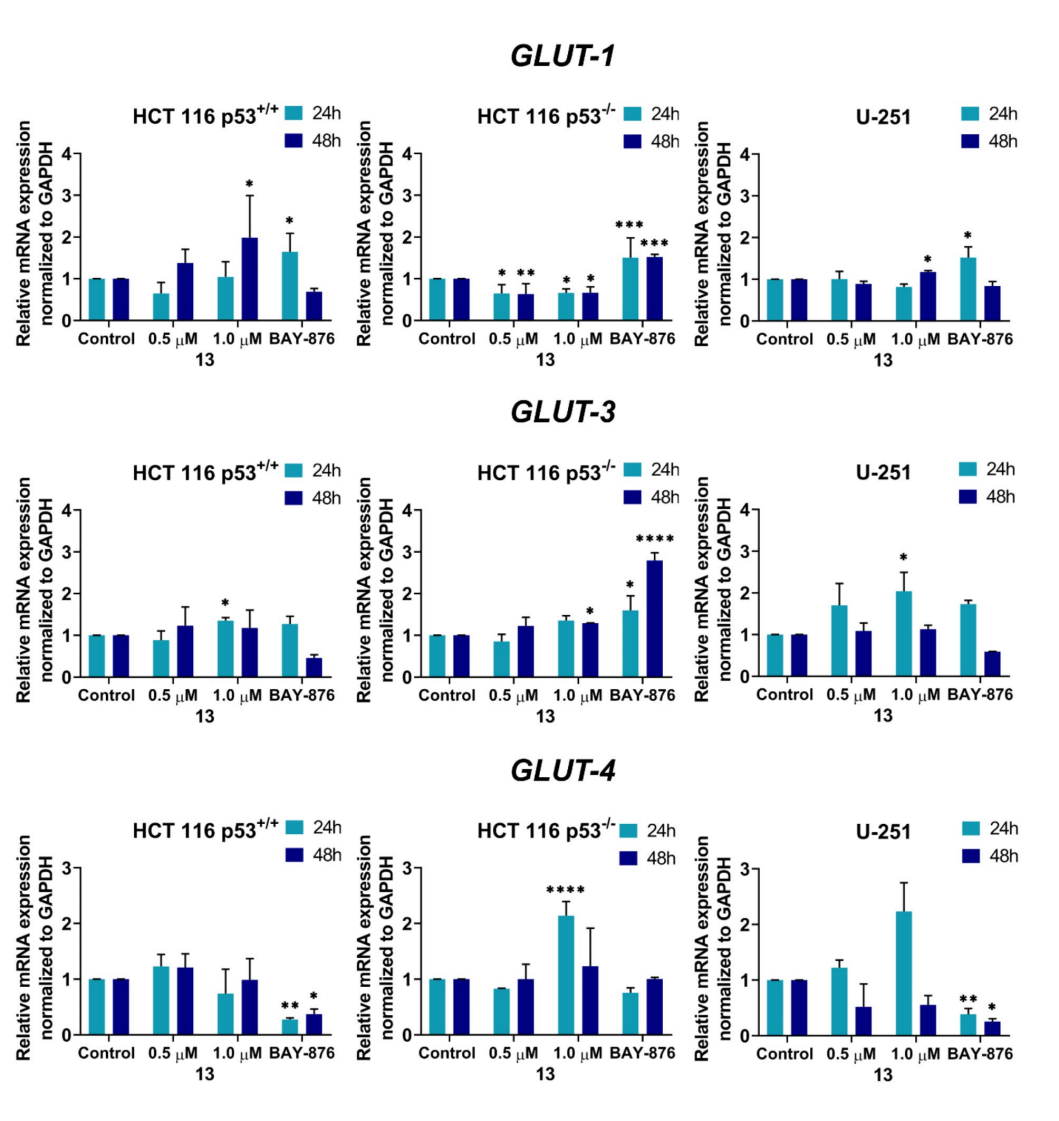
The changes in the mRNA levels of *GLUT-1*, *GLUT-3*, and *GLUT-4* in the HCT 116 p53^+/+^, HCT 116 p53^−/−^ and U-251 cell lines after a 24- and 48-h treatment with derivative **13** and BAY-876. The results are presented as the mean ± SD of three independent experiments. The statistical analysis was performed using a one-way ANOVA with Bonferroni’s post-hoc test: *p < 0.05, **p < 0.01, ***p < 0.001, ****p < 0.0001 compared to the untreated cells (control).

To verify the effect of derivative **13** on glucose transporters at the mRNA level, the expression of the genes encoding the best-characterised glucose transporters in cells was quantified in real-time. In addition to *GLUT-1*, the effect of the tested derivative on changes in the expression of *GLUT-3* and *GLUT-4* was also evaluated (Fig. 8). Furthermore, the inhibitor BAY-876, whose structure is based on a quinoline backbone, was used as a positive control. BAY-876 inhibits all glucose transporter proteins, GLUT1-4, with a preference for GLUT-1 (selectivity over 100 times)^65^. Inhibition of the GLUT proteins may induce hypoxia through metabolic changes, which in turn is the key factor in increasing expression and translocation of the proteins^66^. The results obtained from the analysis of *GLUT-1* expression in the HCT 116 p53^+/+^ cell line correlated with those that were obtained at the protein level. Interestingly, after 24-h, the tested compound caused a greater inhibition of the *GLUT-1* expression than BAY-876, in which an increase in the mRNA level of this gene was observed after this time. The opposite situation was observed after 48 h, when the mRNA level of *GLUT-1* increased after incubation with derivative **13**, whereas it decreased after the application of BAY-876. In the HCT 116 p53^−/−^ line cells, a significant decrease in *GLUT-1* levels was observed after treatment with the furanylvinylquinoline derivative at both time points that were analysed, whereas the BAY-876 treatment increased its expression.

In contrast, in the U-251 glioma line, in which there was a strong inhibition of the *GLUT-1* protein, the test compound itself did not significantly affect the expression of the gene encoding this transporter, nor did BAY-876.

Derivative **13** also did not significantly lead to changes in *GLUT-3* expression in either of the tested HCT 116 lines. In both the wild-type p53 and *TP53* knockout cells, a 24-h incubation with the tested derivative at a concentration of 0.5 µM resulted in a slight decrease in the *GLUT-3* mRNA levels, while the higher concentration caused a slight increase in the expression of the gene encoding this transporter. The observed effect persisted after 48-h as well. The application of BAY-876 caused an increase in *GLUT-3* expression after 24-h. However, after 48-h, a significant decrease in the mRNA levels was observed in the HCT 116 p53^+/+^ cells, whereas in the HCT 116 p53^−/−^ cells, there was a strong increase in the expression of this gene. In the case of the U-251 line, after 24-h, both derivative **13** and the reference BAY-876 induced the upregulation of the *GLUT-3* mRNA levels, whereas after 48-h, a decreasing trend was observed.

After analysing the results for *GLUT-4*, it was concluded that when the derivative **13** was tested in the HCT 116 p53^+/+^ cells, it did not play a significant role in regulating the expression of this gene. No significant changes were observed, in contrast to the samples that were treated with BAY-876, in which there was a significant decrease in the *GLUT-4* mRNA levels. More varied results were obtained in the HCT 116 p53^−/−^ line, in which, after treatment with derivative **13** at a concentration of 0.5 µM, the effect was the same as after incubation with BAY-876. In contrast, the derivative, when tested at a concentration of 1µM, stimulated upregulation of *GLUT-4* mRNA. In the U-251 glioma cells, derivative **13** at a higher concentration caused an increase in the mRNA level of this glucose transporter after 24-h, while after 48-h there was a clear inhibition of *GLUT-4* expression. However, the expression of this gene was more stongly inhibited by BAY-876.

To summarise the results, it was concluded that an inhibitory effect on glycolysis that was dependent on the presence of the wild-type p53 protein was observed in the HCT 116 p53^+/+^ cells. According to the literature, p53 can inhibit glucose transport directly by reducing the expression of the genes encoding *GLUT-1* and *GLUT-4*^67^ and also indirectly by inhibiting *GLUT-3* transcription as a result of the inhibition of the nuclear factor NF-κB^68^. In contrast, the observed effect in the HCT 116 p53^−/−^ cells contradicted the involvement of the p53 protein in the inhibition of the expression of the glucose transporters, in particular *GLUT-1*. Similar conclusions were drawn after analysing the expression profiles of the genes that were studied in glioma cells with mutp53. In this case, one explanation is that p53 plays conflicting roles in glucose metabolism. On the one hand, it inhibits glycolysis, which prevents ATP production, while on the other hand, it promotes oxidative phosphorylation to maximise the production of the 32–34 molecules of ATP by utilising a small amount of pyruvate in the mitochondria. The p53 protein may balance the maintenance of glucose homeostasis with the generation of ROS, which is mediated by oxidative phosphorylation^25^. In contrast, tumour-associated mutp53 does not affect *GLUT-1* expression but stimulates the Warburg effect by promoting the translocation of this glucose transporter to the plasma membrane. However, mutp53 has been shown to largely abrogate the stimulatory effect of the mutations on the Warburg effect in cells^69^. This fact underscores the fact that the results obtained in the U-251 line after the derivative **13** treatment are very promising for the future.

In addition, Zhao et al. observed that a dihydroxy derivative of flavone reduced glycolysis and the proliferation of cancer cells that expressed the wild-type p53 protein but not mutp53^70^. In contrast to this compound, derivative **13** downregulated *GLUT-1* expression and inhibited the growth of the U-251 line cells that were tested with mutp53. It is worth noting that the authors of the above studies ruled out the induction of apoptosis *via* the generation of ROS. On the other hand, in the analysis of the current results, the pronounced overproduction of intracellular ROS in all three cell lines should also be considered. According to the literature, a rapid stimulation of glucose uptake can occur at the level of the regulation of the intrinsic activity of the glucose transporters as well as their translocation, which is induced by ROS-induced signals. This is part of the adaptive mechanism that is activated by oxidative stress^11^. Increased ROS level by stabilization of HIF-1 may enforce the expression of GLUTs, as well as further metabolic changes in cancer cells^71^. The inclusion of such a metabolic pathway could have contributed to the effects that were observed at the mRNA levels of the glucose transporters, which were studied in a manner that was independent of the p53 protein status. Most likely, however, the inhibition of GLUTs, particularly GLUT-1, only complemented the molecular mechanism of the action of derivative **13** based on the generation of intracellular ROS, which resulted in the induction of the extrinsic apoptosis pathway. The observed effect is always a result of various factors, as evidenced in our studies comparing ROS secretion, differential MnSOD response, and the impact on GLUT. Therefore, additional studies correlating GLUT receptor expression with other metabolic pathways altered by compound 13 are necessary.

## Conclusions

In light of the reports of the antiproliferative activity of 2-furanylvinylquinolines against cancer cells, we decided to synthesise new quinoline derivatives with furanyl group (**1–8**). We showed that the anticancer activity increased when the phenyl ring was substituted with a strongly electron-withdrawing group, e.g., nitro or cyano. Therefore, new analogues of 2-furanylvinylquinoline with various substituents in the furanyl part (**1–8**) were designed. The new derivatives were based on the structure of 8-hydroxy (**1–3**, **6–7**), 8-acetoxy (**4**, **8**) and 4-hydroxyquinoline (**5**). The paper presents an in-depth analysis of the structure-activity relationship of derivatives from the styrylquinoline and furanylvinylquinoline groups and focuses on the substitution site of the quinoline ring and the effect of substituents on this activity. It was concluded that the presence of strongly electron-withdrawing substituents, especially the nitro group, favourably affects the anticancer activity of this group of compounds. We further selected the most active derivative **13**, in which the 5-nitrofuranylvinyl substituent was attached to the C4 position of the quinoline ring, and verified its mechanism of action by extending the panel of tested cells to lines that differed in their p53 protein status. Against most of the tested cancer lines, the IC_50_ of this compound gave values below 100 nM.

In a further stage of the research, a series of experiments were performed to determine the molecular mechanism of action of this derivative, including a cell cycle analysis and the induction of cell death. Experiments using microcapillary flow cytometry showed cell cycle inhibition in the S (in the U251 cell line) or G2/M phases (in both the HCT 116 cell lines), which confirmed the hypothesis of the antiproliferative action of the tested compounds. In addition, the mRNA level of the *CDK1* gene encoding one of the cell cycle regulation proteins was examined, indicating an upregulating trend, which was confirmed at the protein level by Westen Blot experiments. CDK1 is a factor that is involved in two signalling pathways that are associated with the regulation of the G2/M phase transition in the cell cycle. One of these is related to checkpoint kinase 1 (CHK-1) and cell division cycle 25 C (CDC25C). In the second, the signalling pathway involves p53 and p21^WAF1^.

After performing Western Blot analysis, the following correlation was noted, namely, p21 protein activation occurred in a p53-independent manner. This was evidenced by the observed upregulation of p21 expression in the HCT116 line cells of both types and the lack of detection in the U251 line. In addition, subsequent experiments proved the induction of cell death through the apoptotic pathway. According to the general observations, arrest in the G2/M phase often leads to apoptosis. Subsequently, the fluorescence tests that were performed showed changes in the level of the generated ROS and exhibited a significant disruption in the oxidoreductive system of the cancer cells. The results clearly showed the induction of oxidative stress after treatment with derivative **13**. A marked increase in the level of ROS was already observed after 3–6 h, while after 24-h, this level was 20 to 50% higher than in the control sample of the untreated cells. In addition, the effect of derivative **13** on the expression of the genes encoding two enzymes involved in the antioxidant pathway, MnSOD, and CAT, was examined at the mRNA level. The mRNA level of *MnSOD* was especially increased in the cells with p53 (HCT 116 p53^+/+^ and U-251) in comparison to that with p53 deletion. The results confirmed the significant interaction of the compound with these molecular targets, which ultimately led to cell apoptosis. The above results were also confirmed by evaluating changes in the expression of the individual proteins that are specific to the cell cycle and apoptosis, as well as those that are involved in the pathways that are associated with oxidative stress. Studies that focused on determining the mechanism of the action of derivative **13** revealed a major role for the p53 protein, which affected the time-dependent activation of the molecular targets associated with the metabolic processes that were analysed. The tested derivative activated one of the oxidative stress molecules, namely HO-1, thereby highlighting its influence on the cellular redox imbalance. Analysis of the effect of derivative **13** on the expression of the genes encoding the markers of the extrinsic apoptosis pathway, such as the TRAIL ligand, DR5 receptor, and caspase-8, suggested the compound acted *via* the cell death pathway. In addition, in the case of the colorectal cancer lines, this was confirmed by the Fas protein. Another interesting result is that derivative **13** exhibited inhibitory properties on the expression of the glucose transporter GLUT-1, thus revealing new insights into the action of the compounds in this class and opening up new possibilities in the development of anticancer therapies. The mechanism of action of derivative **13** is undoubtedly related to the induction of oxidative stress in cells *via* the generation of ROS. The result is a breakdown of the antioxidant system, which consequently leads to apoptosis.

## Electronic supplementary material

Below is the link to the electronic supplementary material.

Supplementary Material 1

Supplementary Material 2

Supplementary Material 3

Supplementary Material 4

Supplementary Material 5

Supplementary Material 6

Supplementary Material 7

Supplementary Material 8

## Data Availability

The datasets used and analysed during the current study available from the corresponding author (anna.mrozek-wilczkiewicz@us.edu.pl) on reasonable request.
