# Supplementary material for: 4-Furanylvinylquinoline derivative as a new scaffold for the design of oxidative stress initiator and glucose transporter inhibitor drugs
Source: Sci Rep. 2024 Nov 18;14:28454. doi: 10.1038/s41598-024-79698-0 (PMC11574108; doi:10.1038/s41598-024-79698-0)
Supplement: Supplementary file 1 — Supplementary Material 1 [file 41598_2024_79698_MOESM1_ESM.pptx]

## Slide 1
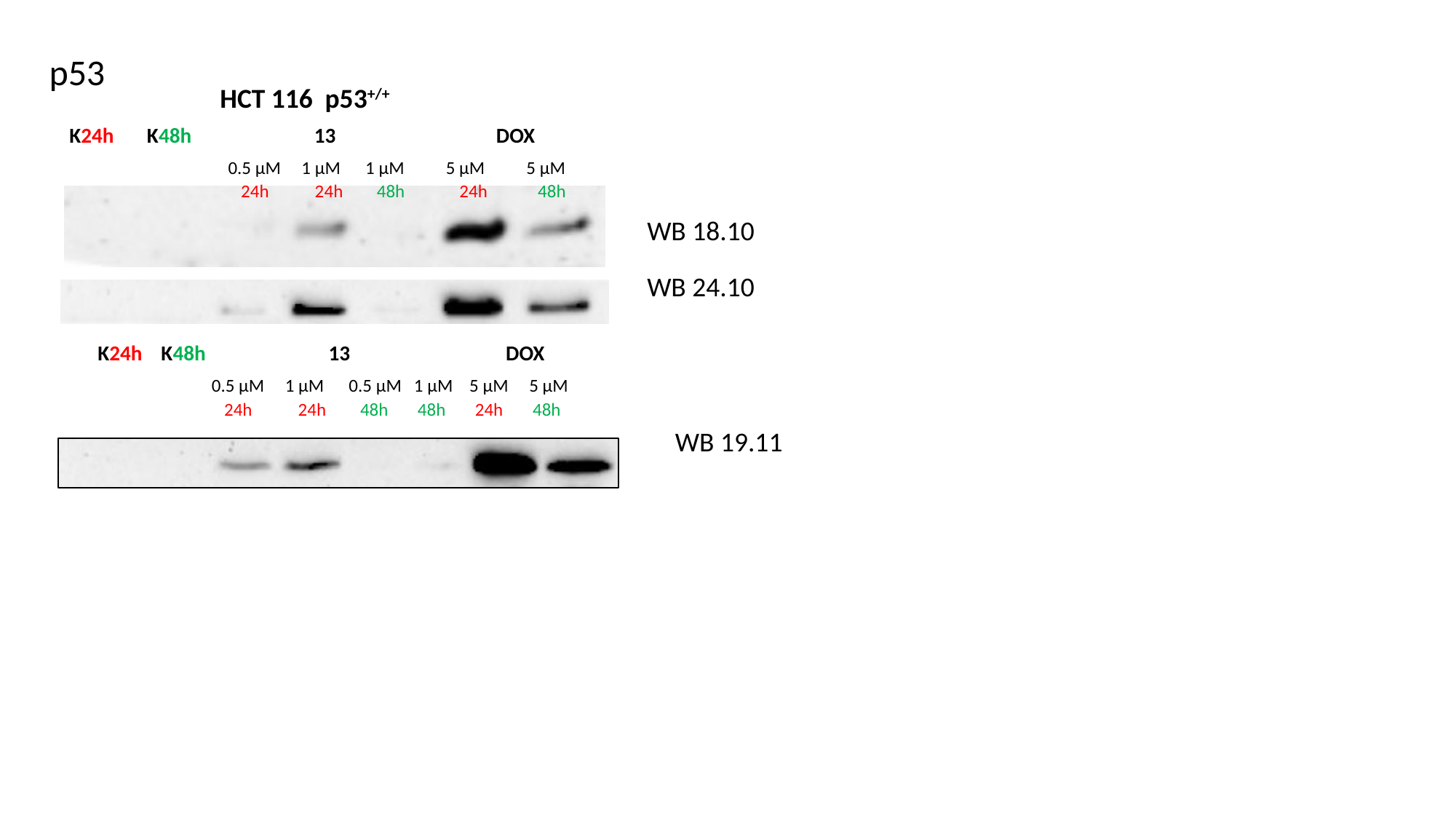

p53
HCT 116 p53+/+
K24h K48h 13 DOX
 0.5 µM 1 µM 1 µM 5 µM 5 µM
 24h 24h 48h 24h 48h
WB 18.10
WB 24.10
 K24h K48h 13 DOX
 0.5 µM 1 µM 0.5 µM 1 µM 5 µM 5 µM
 24h 24h 48h 48h 24h 48h
WB 19.11

## Slide 2
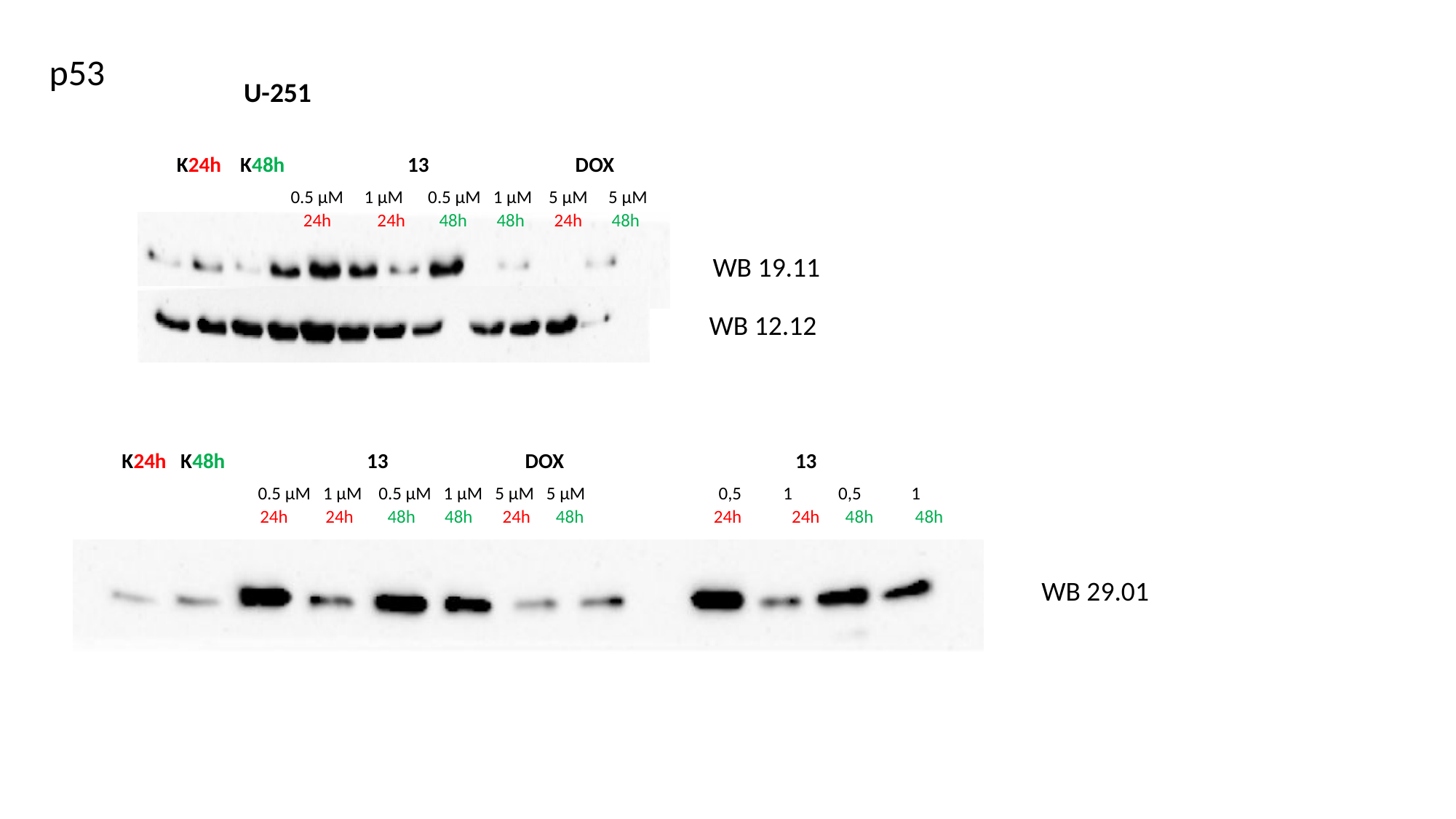

p53
U-251
 K24h K48h 13 DOX
 0.5 µM 1 µM 0.5 µM 1 µM 5 µM 5 µM
 24h 24h 48h 48h 24h 48h
WB 19.11
WB 12.12
K24h K48h 13 DOX 13
 0.5 µM 1 µM 0.5 µM 1 µM 5 µM 5 µM 0,5 1 0,5 1
 24h 24h 48h 48h 24h 48h 24h 24h 48h 48h
WB 29.01

## Slide 3
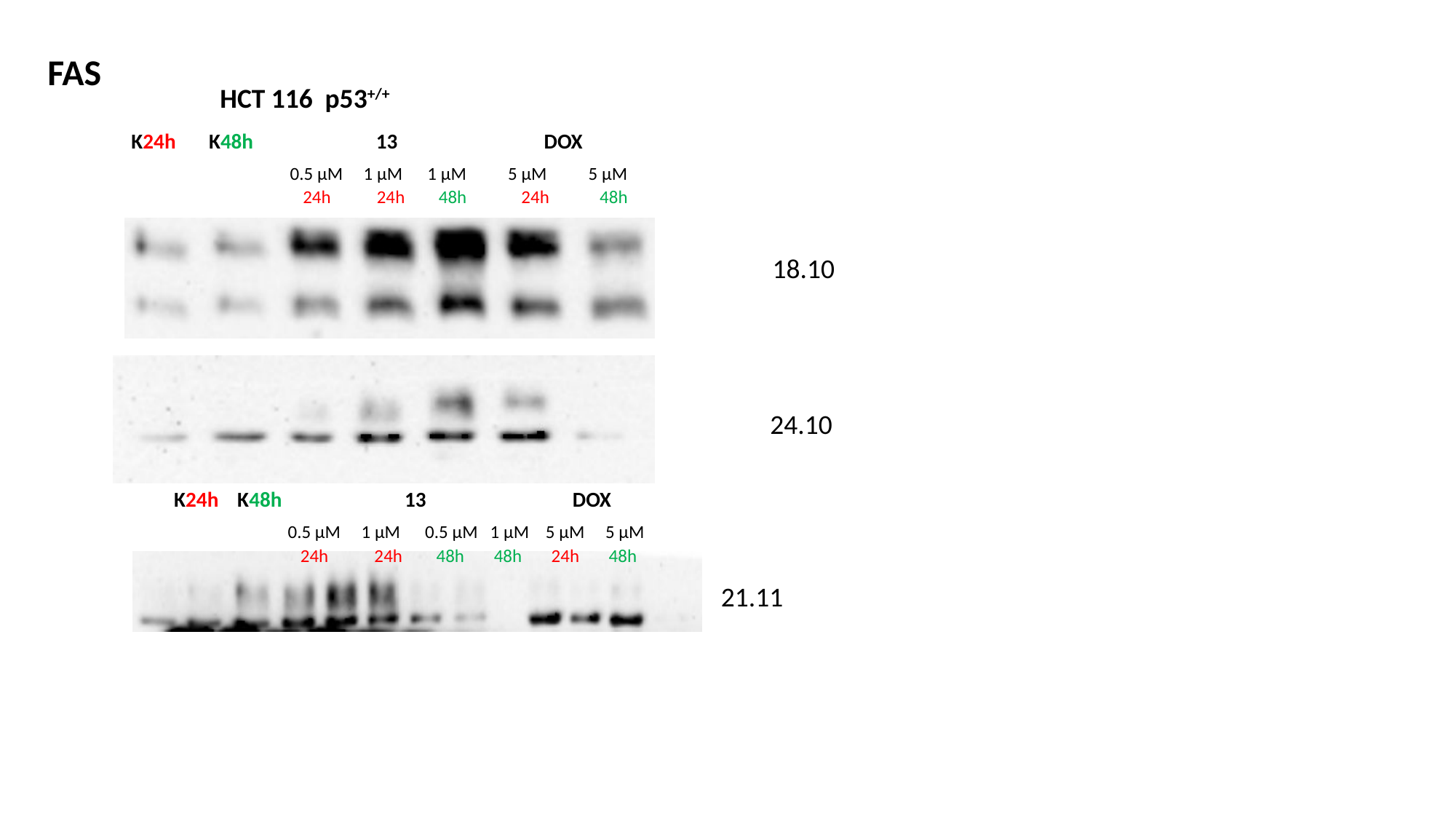

FAS
HCT 116 p53+/+
K24h K48h 13 DOX
 0.5 µM 1 µM 1 µM 5 µM 5 µM
 24h 24h 48h 24h 48h
18.10
24.10
 K24h K48h 13 DOX
 0.5 µM 1 µM 0.5 µM 1 µM 5 µM 5 µM
 24h 24h 48h 48h 24h 48h
21.11

## Slide 4
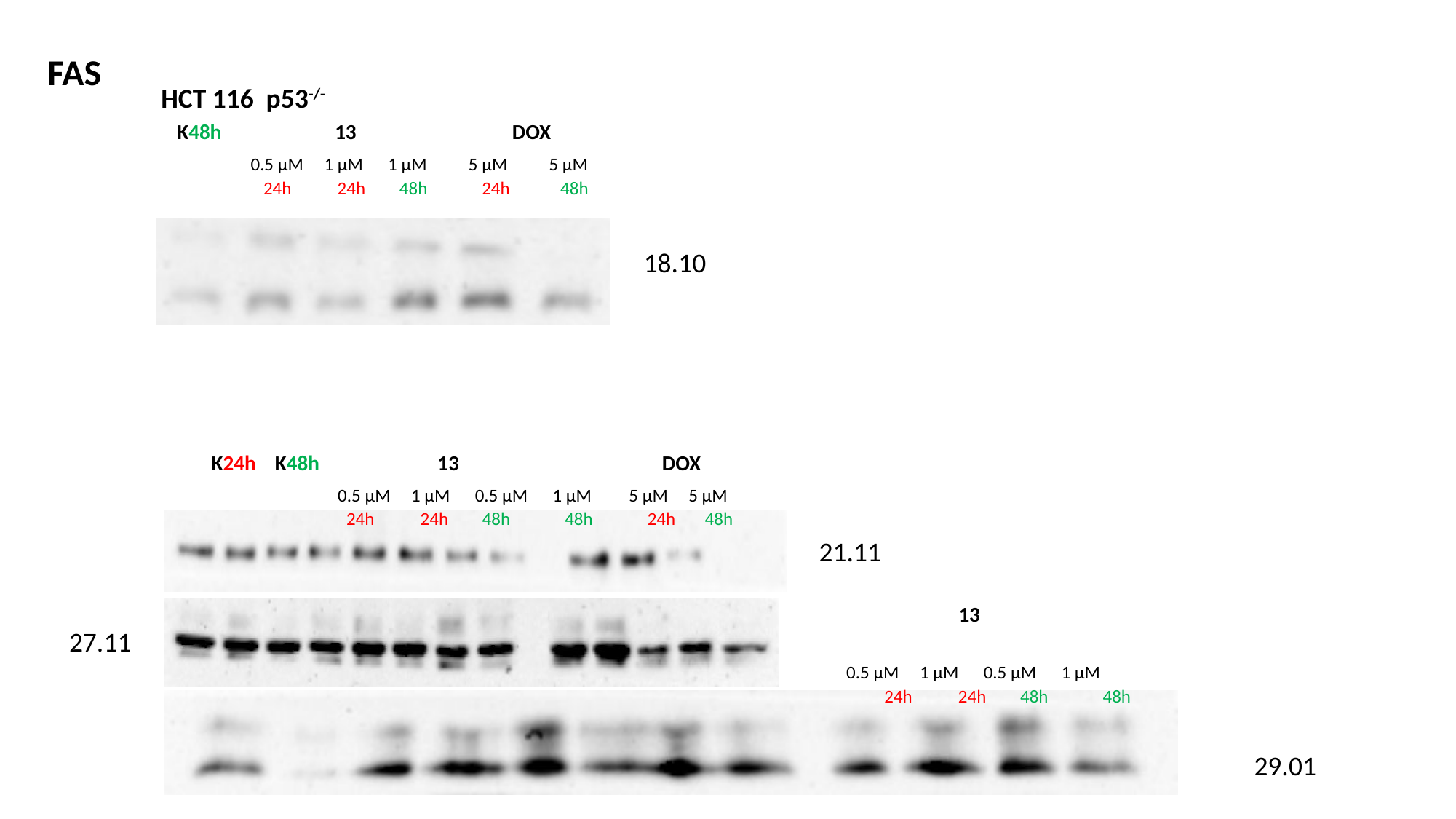

FAS
HCT 116 p53-/-
 K48h 13 DOX
 0.5 µM 1 µM 1 µM 5 µM 5 µM
 24h 24h 48h 24h 48h
18.10
 K24h K48h 13 DOX
 0.5 µM 1 µM 0.5 µM 1 µM 5 µM 5 µM
 24h 24h 48h 48h 24h 48h
21.11
 13
 0.5 µM 1 µM 0.5 µM 1 µM
 24h 24h 48h 48h
27.11
29.01

## Slide 5
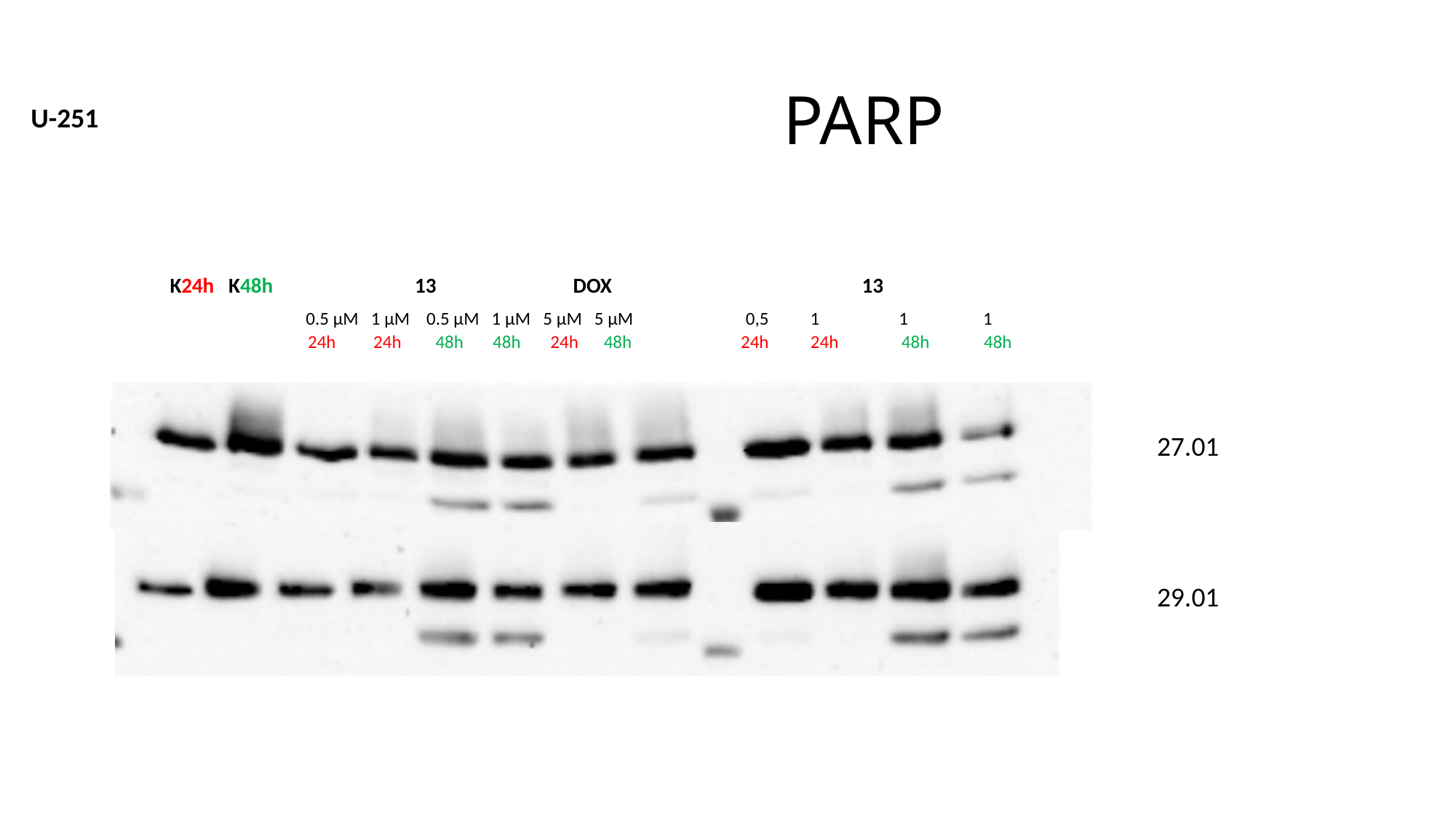

# PARP
U-251
K24h K48h 13 DOX 13
 0.5 µM 1 µM 0.5 µM 1 µM 5 µM 5 µM 0,5 1 1 1
 24h 24h 48h 48h 24h 48h 24h 24h 48h 48h
27.01
29.01

## Slide 6
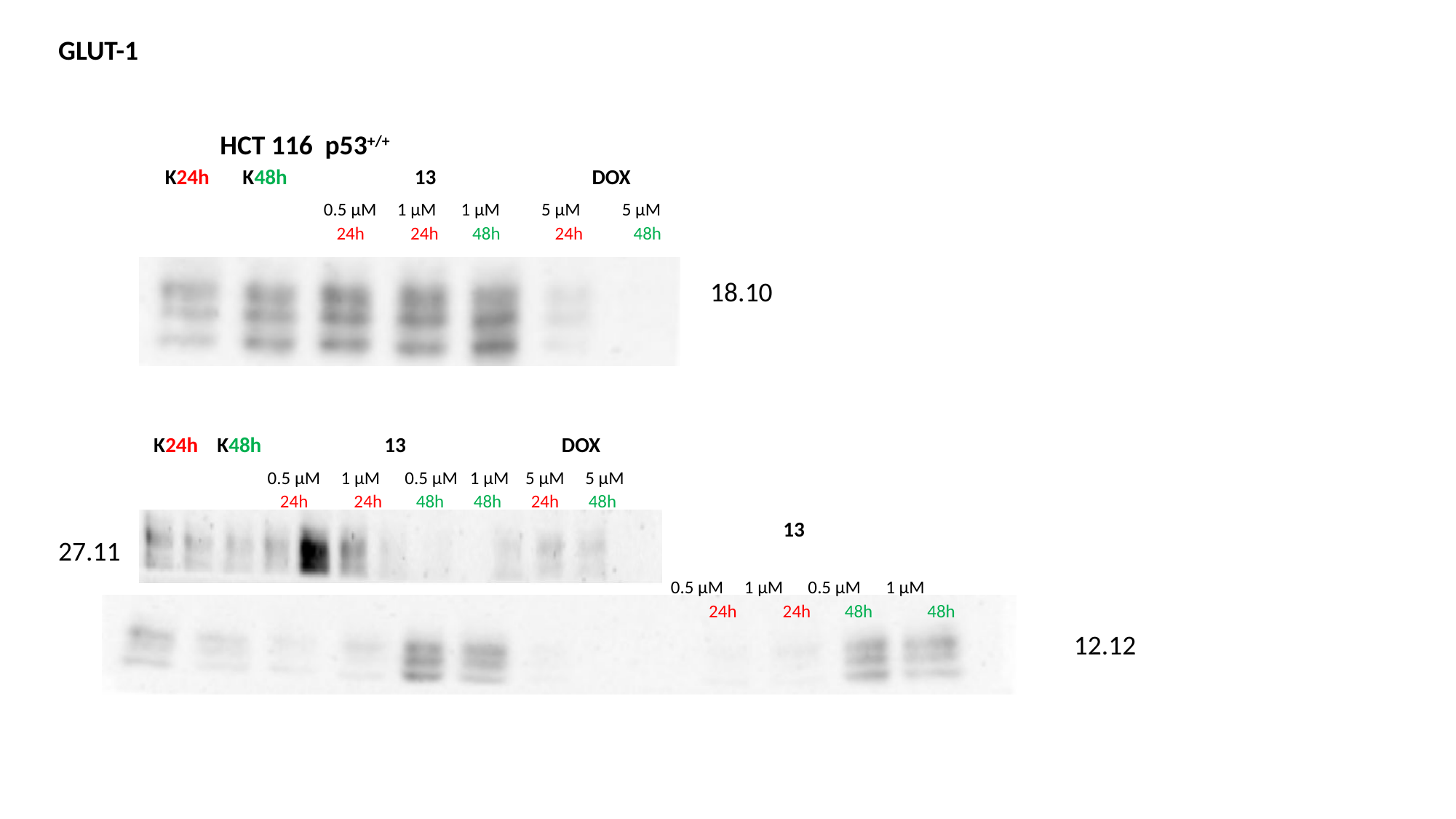

GLUT-1
HCT 116 p53+/+
K24h K48h 13 DOX
 0.5 µM 1 µM 1 µM 5 µM 5 µM
 24h 24h 48h 24h 48h
18.10
 K24h K48h 13 DOX
 0.5 µM 1 µM 0.5 µM 1 µM 5 µM 5 µM
 24h 24h 48h 48h 24h 48h
 13
 0.5 µM 1 µM 0.5 µM 1 µM
 24h 24h 48h 48h
27.11
12.12

## Slide 7
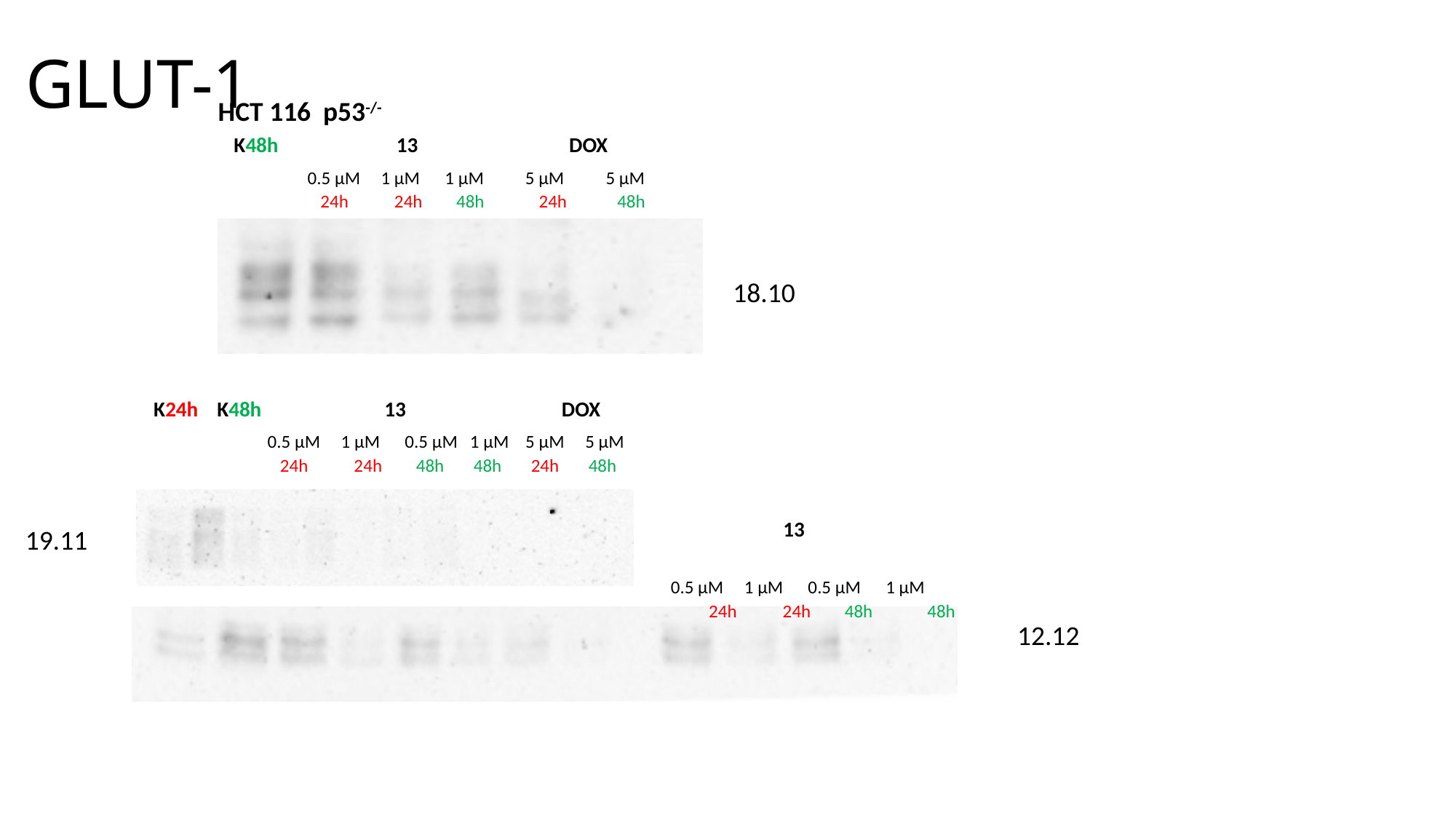

# GLUT-1
HCT 116 p53-/-
 K48h 13 DOX
 0.5 µM 1 µM 1 µM 5 µM 5 µM
 24h 24h 48h 24h 48h
18.10
 K24h K48h 13 DOX
 0.5 µM 1 µM 0.5 µM 1 µM 5 µM 5 µM
 24h 24h 48h 48h 24h 48h
 13
 0.5 µM 1 µM 0.5 µM 1 µM
 24h 24h 48h 48h
19.11
12.12

## Slide 8
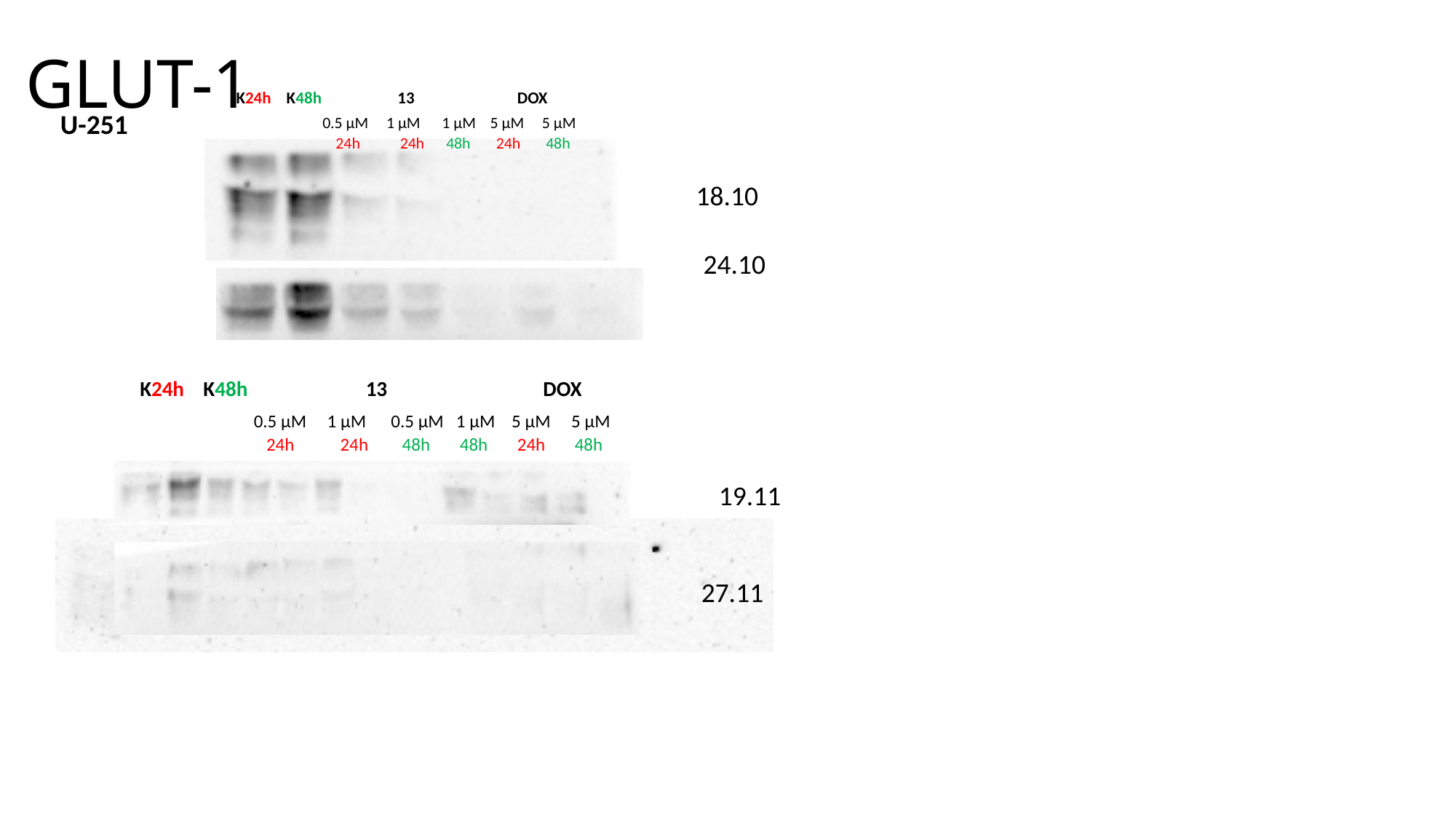

GLUT-1
 K24h K48h 13 DOX
 0.5 µM 1 µM 1 µM 5 µM 5 µM
 24h 24h 48h 24h 48h
U-251
18.10
24.10
 K24h K48h 13 DOX
 0.5 µM 1 µM 0.5 µM 1 µM 5 µM 5 µM
 24h 24h 48h 48h 24h 48h
19.11
27.11

## Slide 9
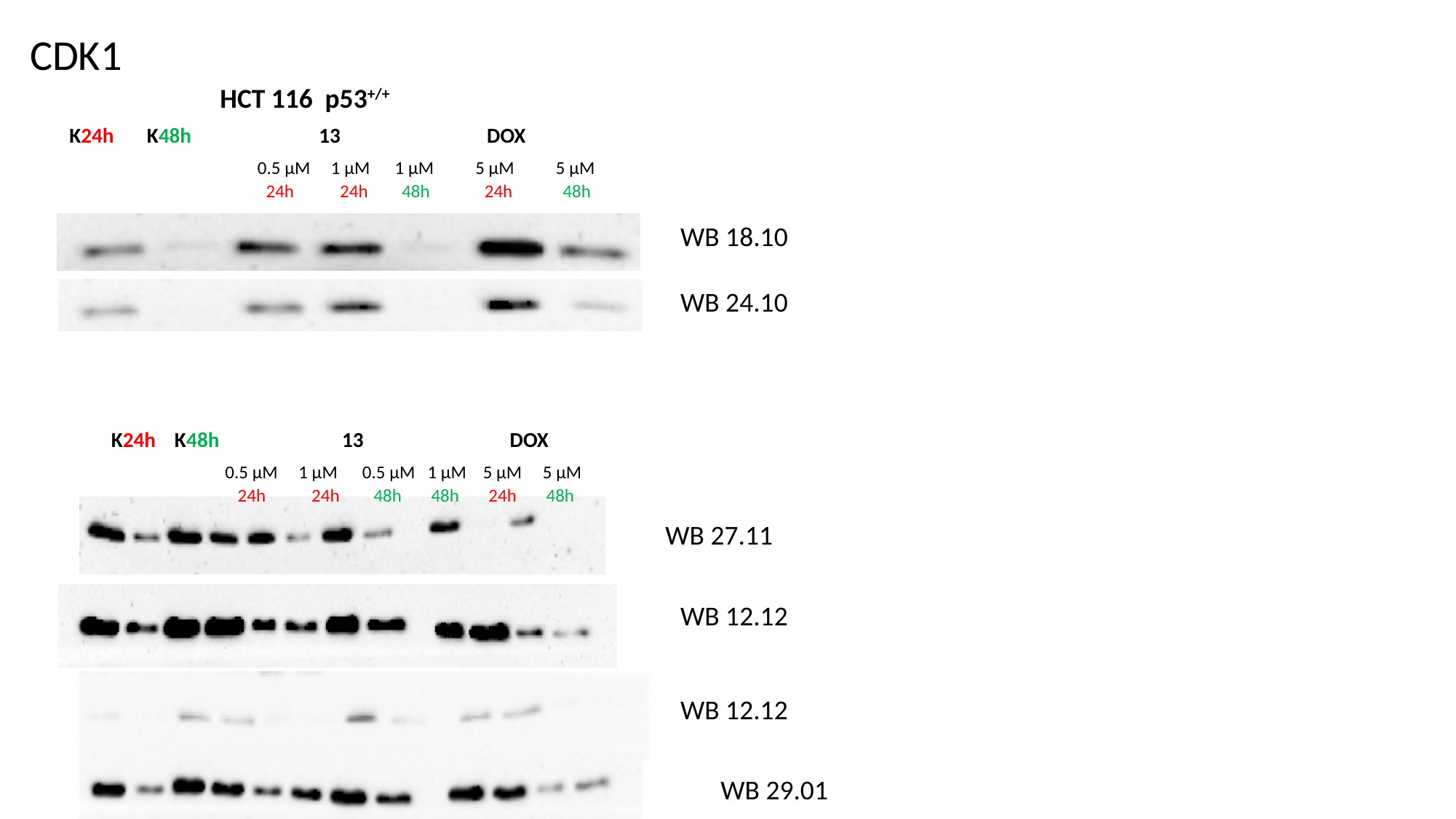

CDK1
HCT 116 p53+/+
K24h K48h 13 DOX
 0.5 µM 1 µM 1 µM 5 µM 5 µM
 24h 24h 48h 24h 48h
WB 18.10
WB 24.10
 K24h K48h 13 DOX
 0.5 µM 1 µM 0.5 µM 1 µM 5 µM 5 µM
 24h 24h 48h 48h 24h 48h
WB 27.11
WB 12.12
WB 12.12
WB 29.01

## Slide 10
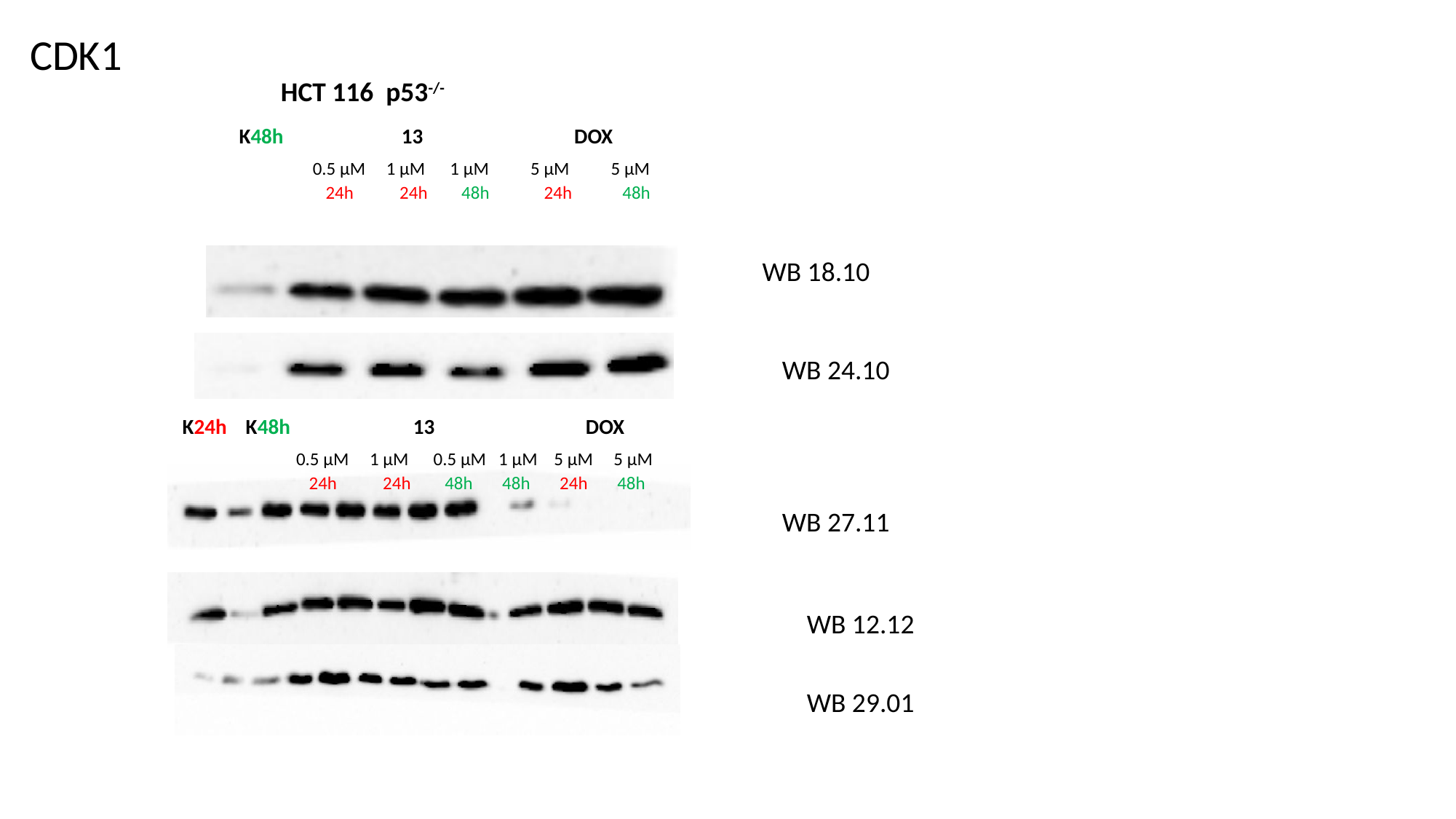

CDK1
HCT 116 p53-/-
 K48h 13 DOX
 0.5 µM 1 µM 1 µM 5 µM 5 µM
 24h 24h 48h 24h 48h
WB 18.10
WB 24.10
 K24h K48h 13 DOX
 0.5 µM 1 µM 0.5 µM 1 µM 5 µM 5 µM
 24h 24h 48h 48h 24h 48h
WB 27.11
WB 12.12
WB 29.01

## Slide 11
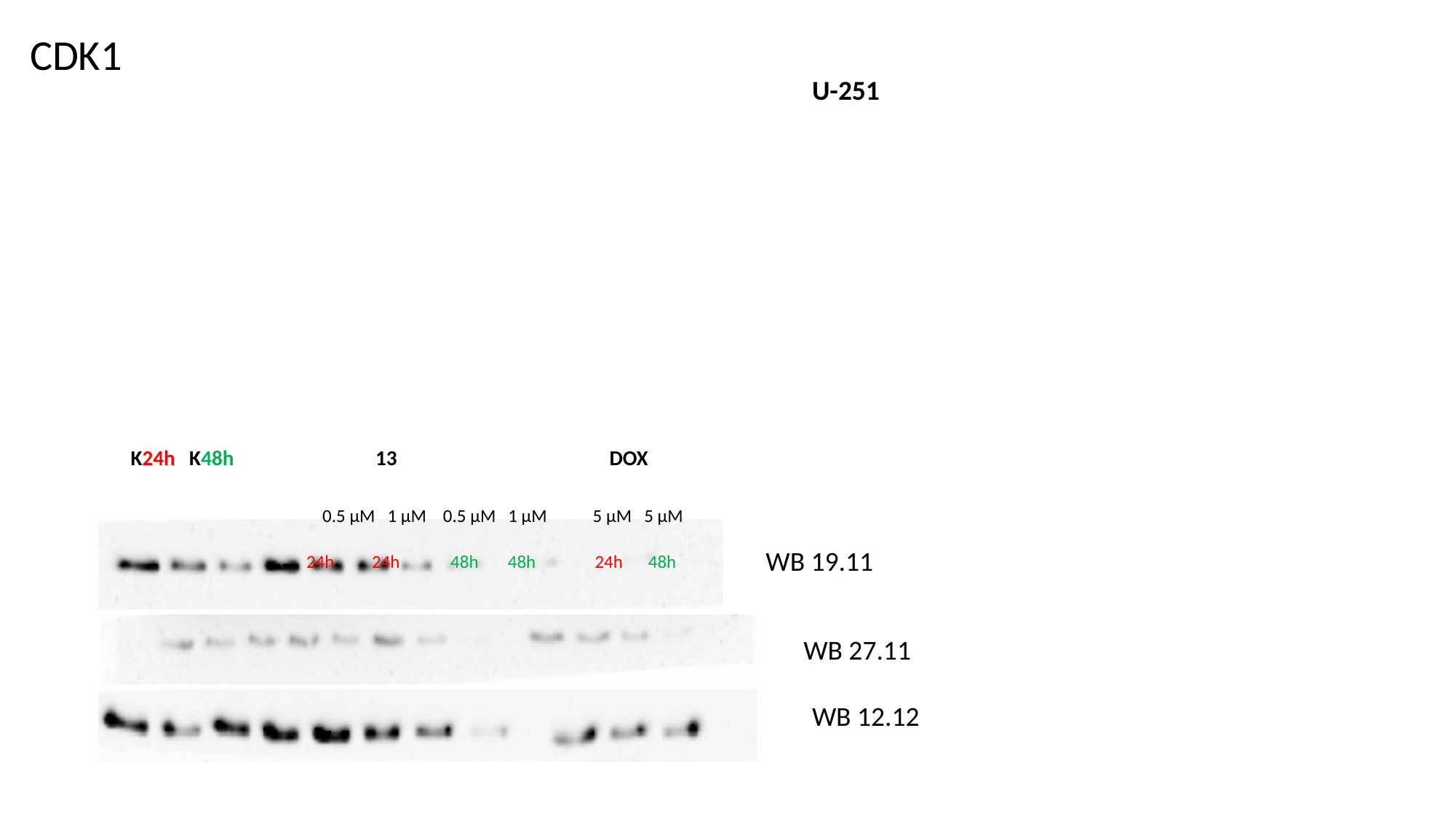

CDK1
U-251
K24h K48h 13 DOX
 0.5 µM 1 µM 0.5 µM 1 µM 5 µM 5 µM
 24h 24h 48h 48h 24h 48h
WB 19.11
WB 27.11
WB 12.12

## Slide 12
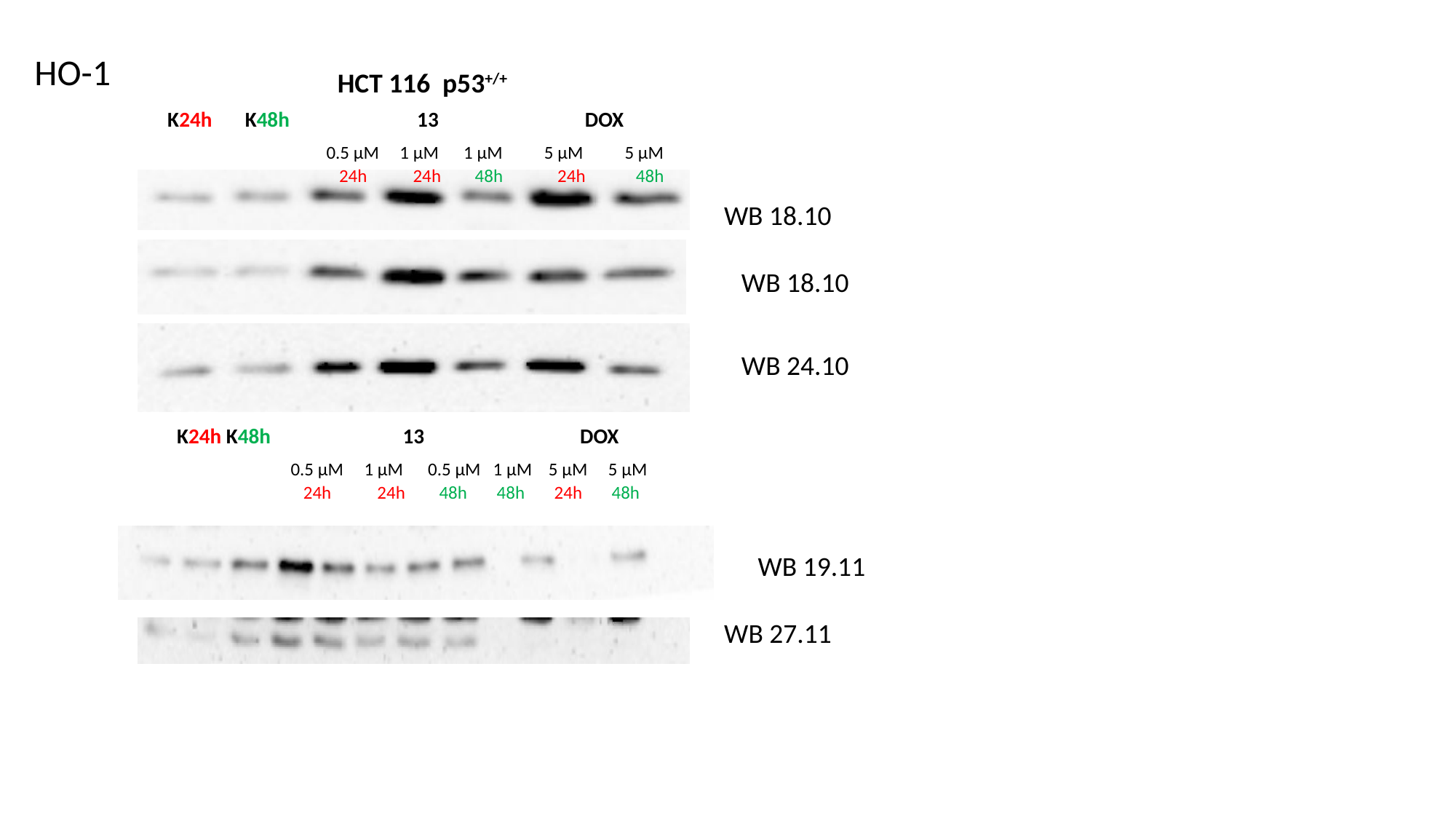

HO-1
HCT 116 p53+/+
K24h K48h 13 DOX
 0.5 µM 1 µM 1 µM 5 µM 5 µM
 24h 24h 48h 24h 48h
WB 18.10
WB 18.10
WB 24.10
 K24h K48h 13 DOX
 0.5 µM 1 µM 0.5 µM 1 µM 5 µM 5 µM
 24h 24h 48h 48h 24h 48h
WB 19.11
WB 27.11

## Slide 13
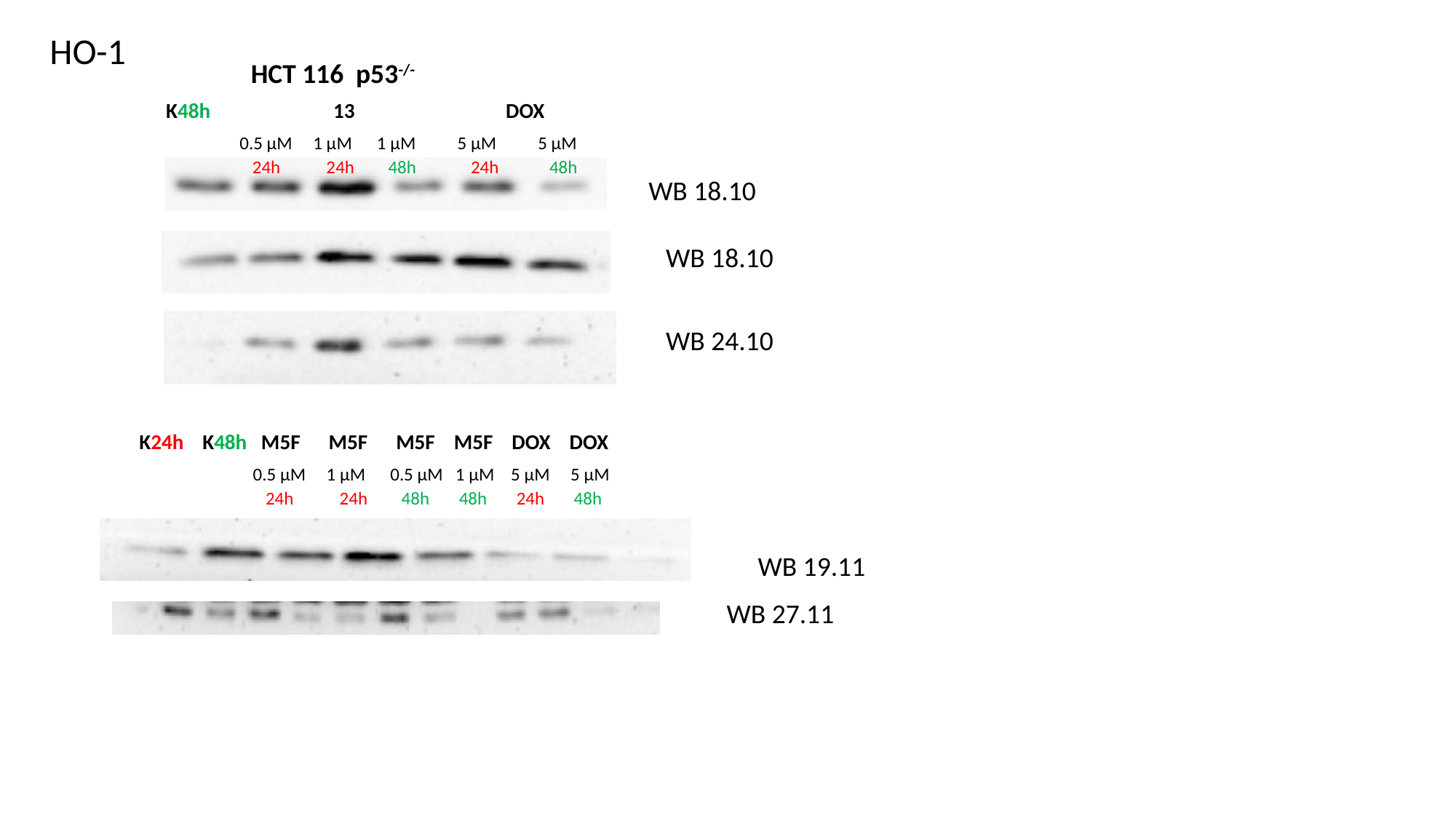

HO-1
HCT 116 p53-/-
 K48h 13 DOX
 0.5 µM 1 µM 1 µM 5 µM 5 µM
 24h 24h 48h 24h 48h
WB 18.10
WB 18.10
WB 24.10
 K24h K48h M5F M5F M5F M5F DOX DOX
 0.5 µM 1 µM 0.5 µM 1 µM 5 µM 5 µM
 24h 24h 48h 48h 24h 48h
WB 19.11
WB 27.11

## Slide 14
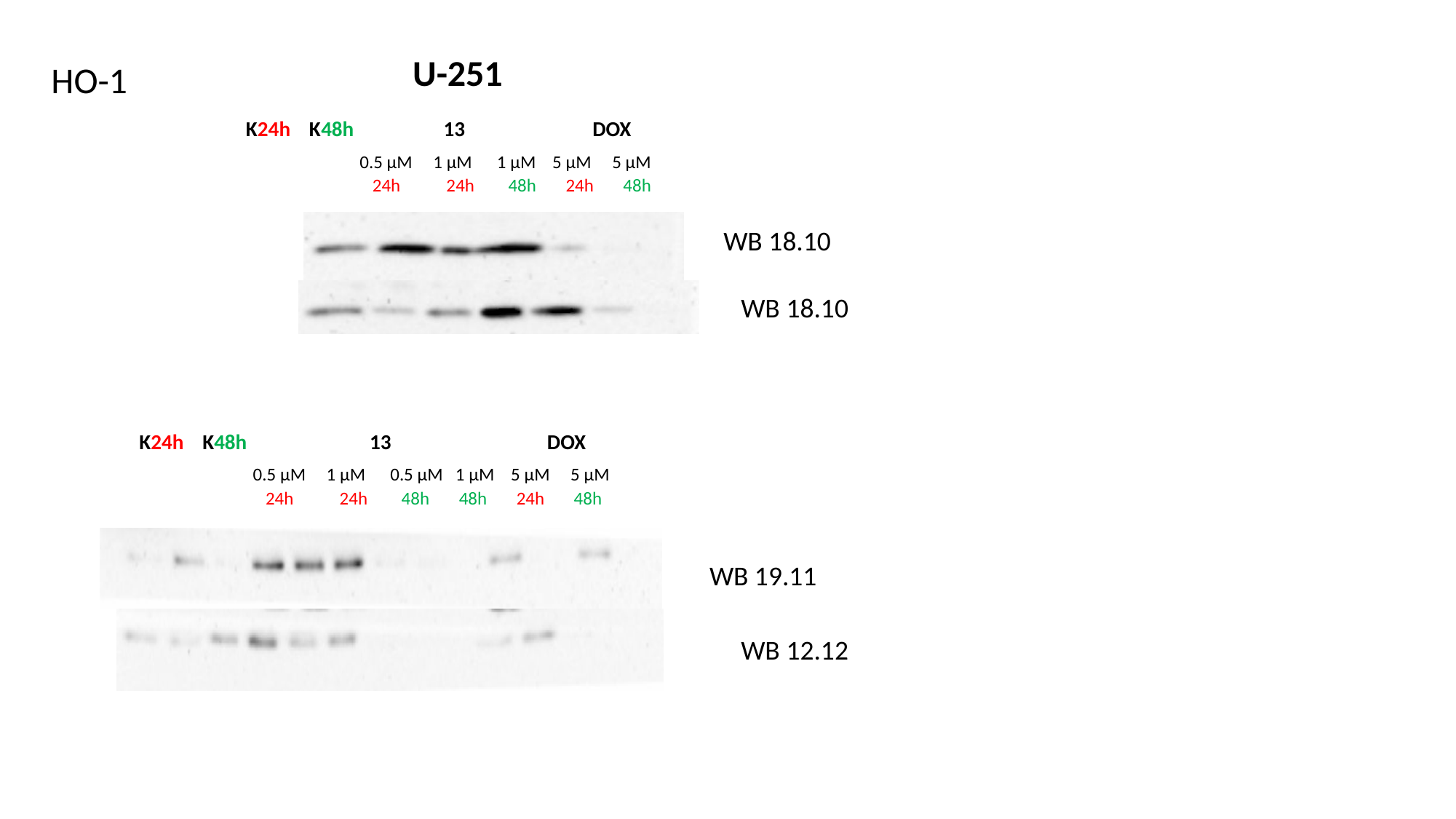

U-251
HO-1
 K24h K48h 13 DOX
 0.5 µM 1 µM 1 µM 5 µM 5 µM
 24h 24h 48h 24h 48h
WB 18.10
WB 18.10
 K24h K48h 13 DOX
 0.5 µM 1 µM 0.5 µM 1 µM 5 µM 5 µM
 24h 24h 48h 48h 24h 48h
WB 19.11
WB 12.12

## Slide 15
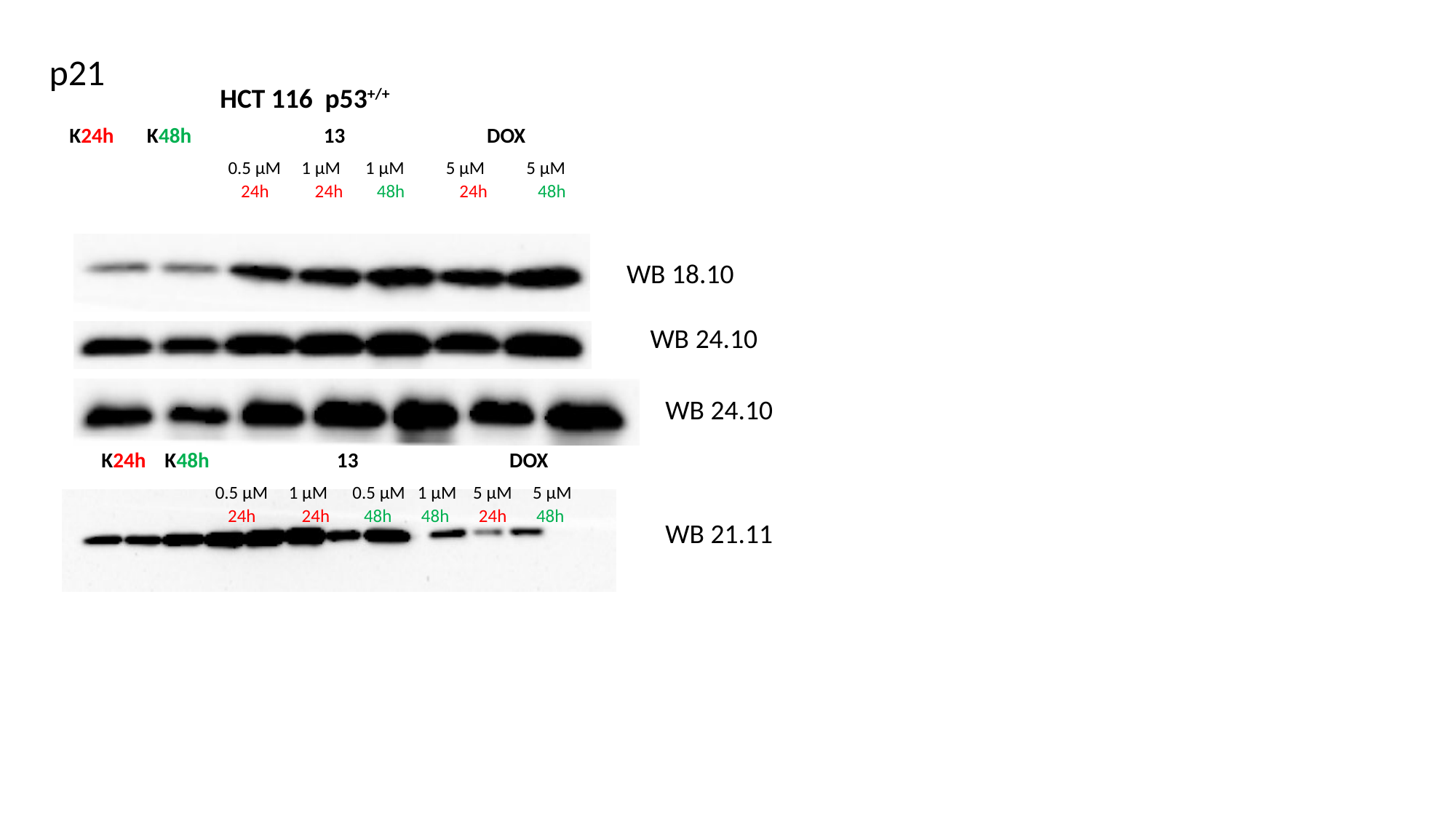

p21
HCT 116 p53+/+
K24h K48h 13 DOX
 0.5 µM 1 µM 1 µM 5 µM 5 µM
 24h 24h 48h 24h 48h
WB 18.10
WB 24.10
WB 24.10
 K24h K48h 13 DOX
 0.5 µM 1 µM 0.5 µM 1 µM 5 µM 5 µM
 24h 24h 48h 48h 24h 48h
WB 21.11

## Slide 16
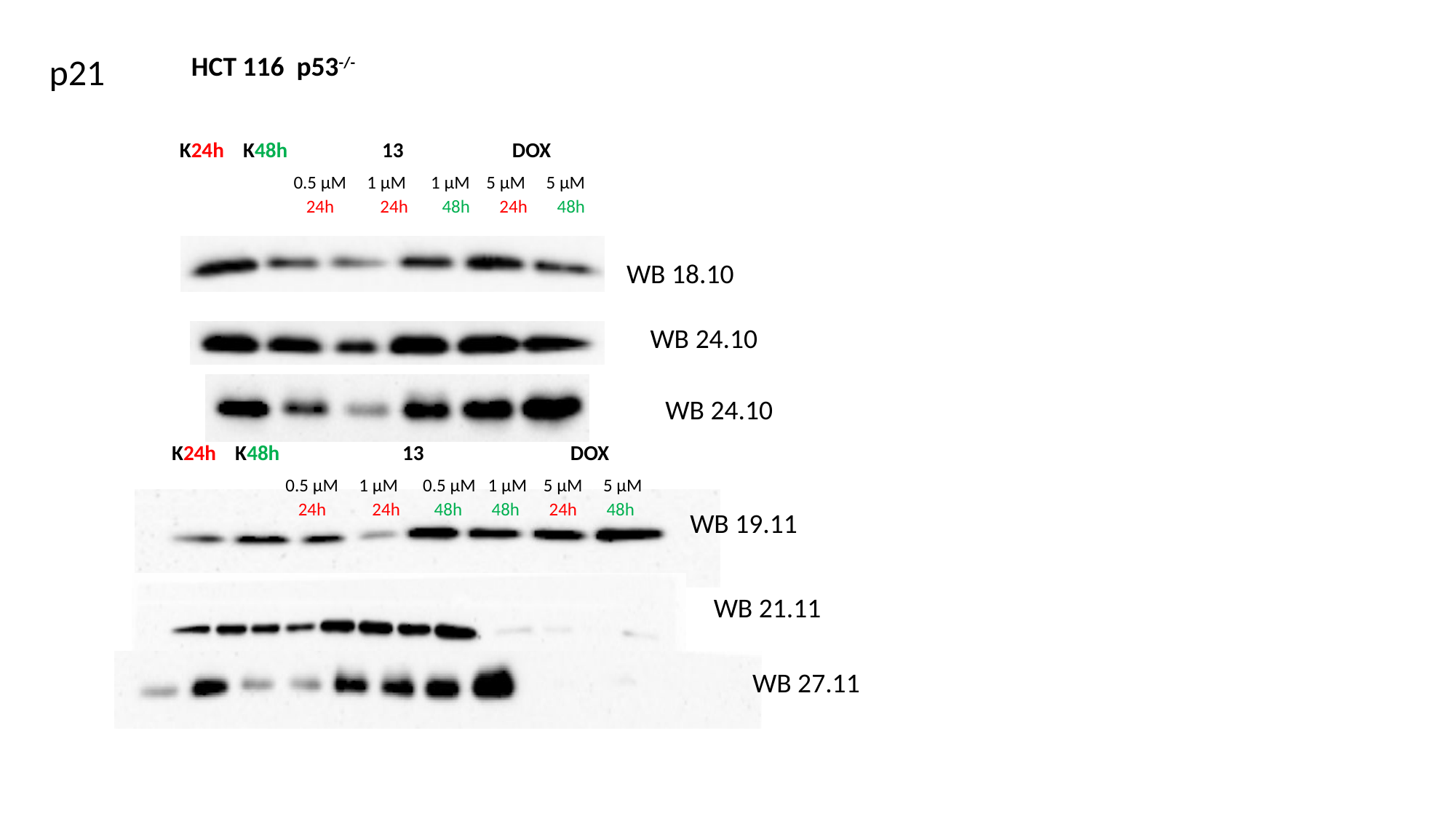

p21
HCT 116 p53-/-
 K24h K48h 13 DOX
 0.5 µM 1 µM 1 µM 5 µM 5 µM
 24h 24h 48h 24h 48h
WB 18.10
WB 24.10
WB 24.10
 K24h K48h 13 DOX
 0.5 µM 1 µM 0.5 µM 1 µM 5 µM 5 µM
 24h 24h 48h 48h 24h 48h
WB 19.11
WB 21.11
WB 27.11
