# Supplementary figures and images for: 4-Furanylvinylquinoline derivative as a new scaffold for the design of oxidative stress initiator and glucose transporter inhibitor drugs
Source: Sci Rep. 2024 Nov 18;14:28454. doi: 10.1038/s41598-024-79698-0 (PMC11574108; doi:10.1038/s41598-024-79698-0)

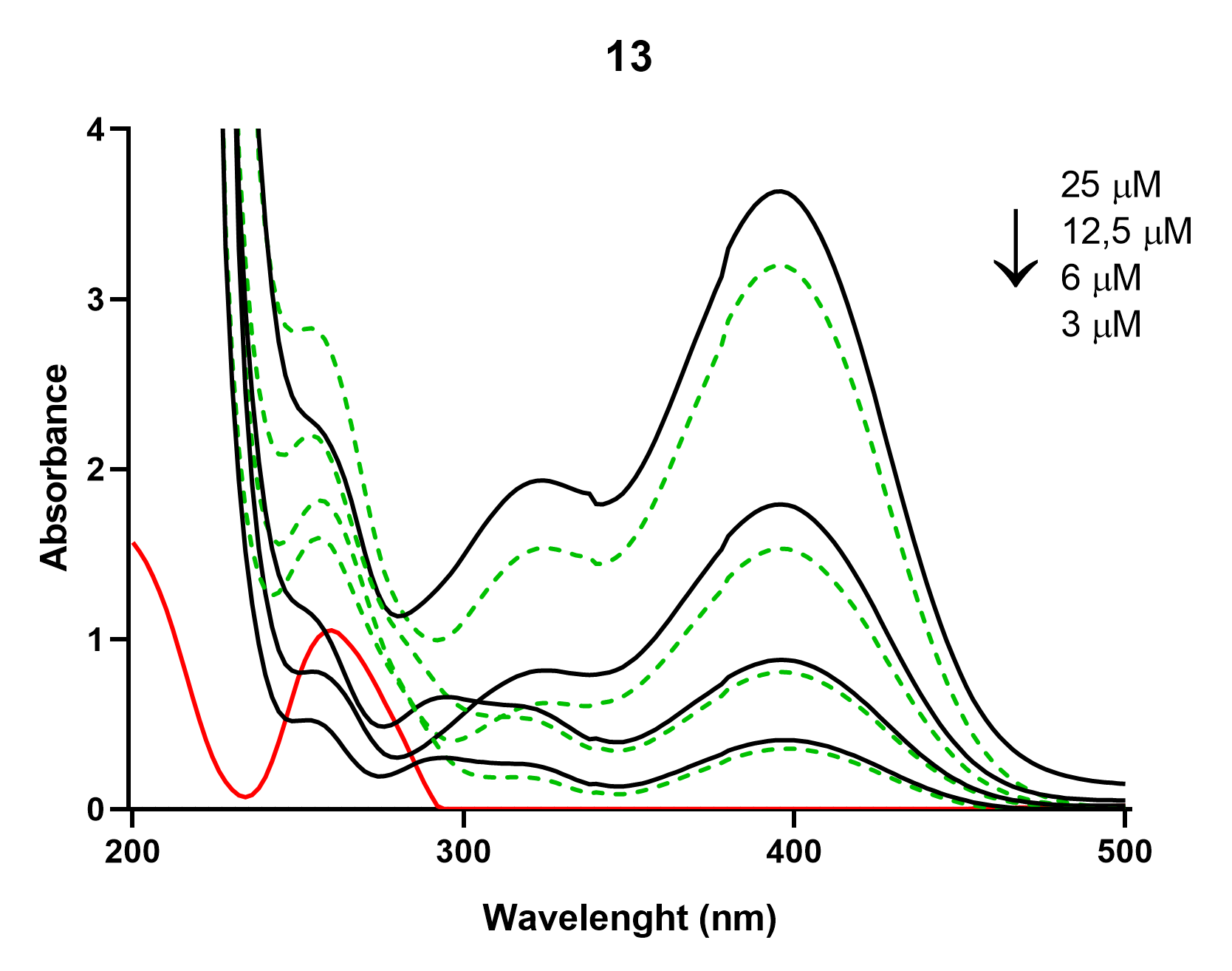

Supplement: Supplementary file 2 — Supplementary Material 2 [file 41598_2024_79698_MOESM2_ESM.tif]

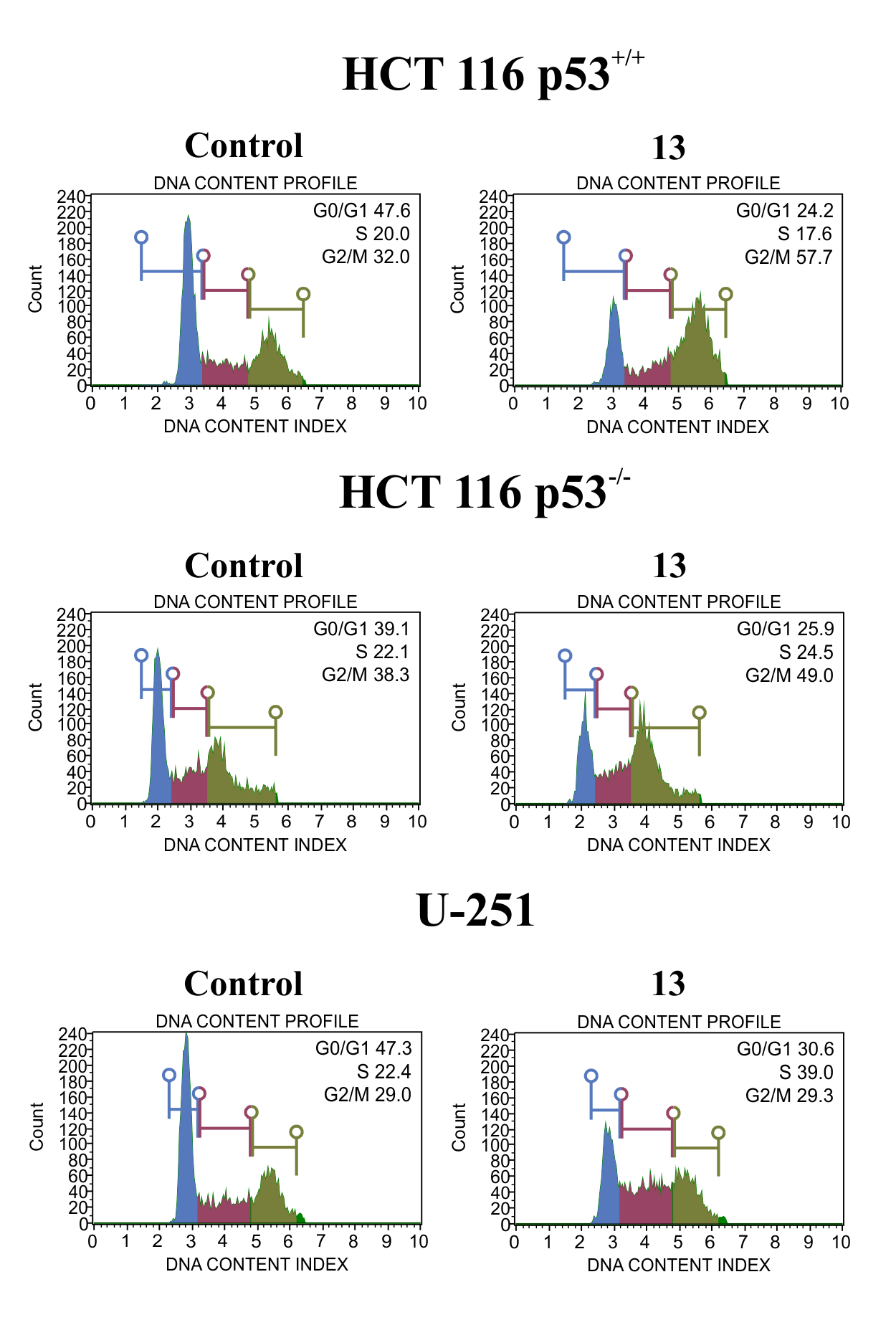

Supplement: Supplementary file 3 — Supplementary Material 3 [file 41598_2024_79698_MOESM3_ESM.tif]

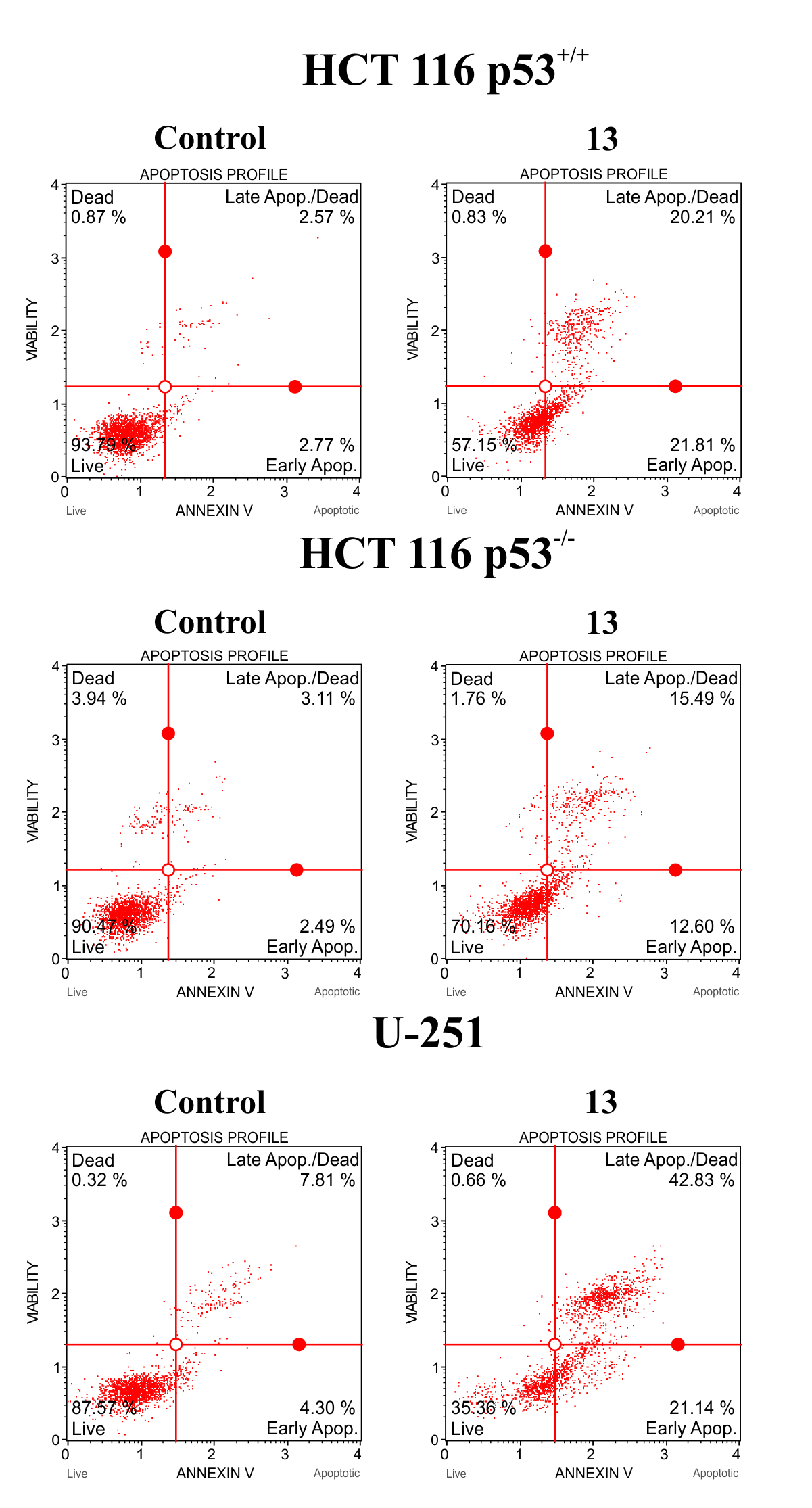

Supplement: Supplementary file 4 — Supplementary Material 4 [file 41598_2024_79698_MOESM4_ESM.tif]

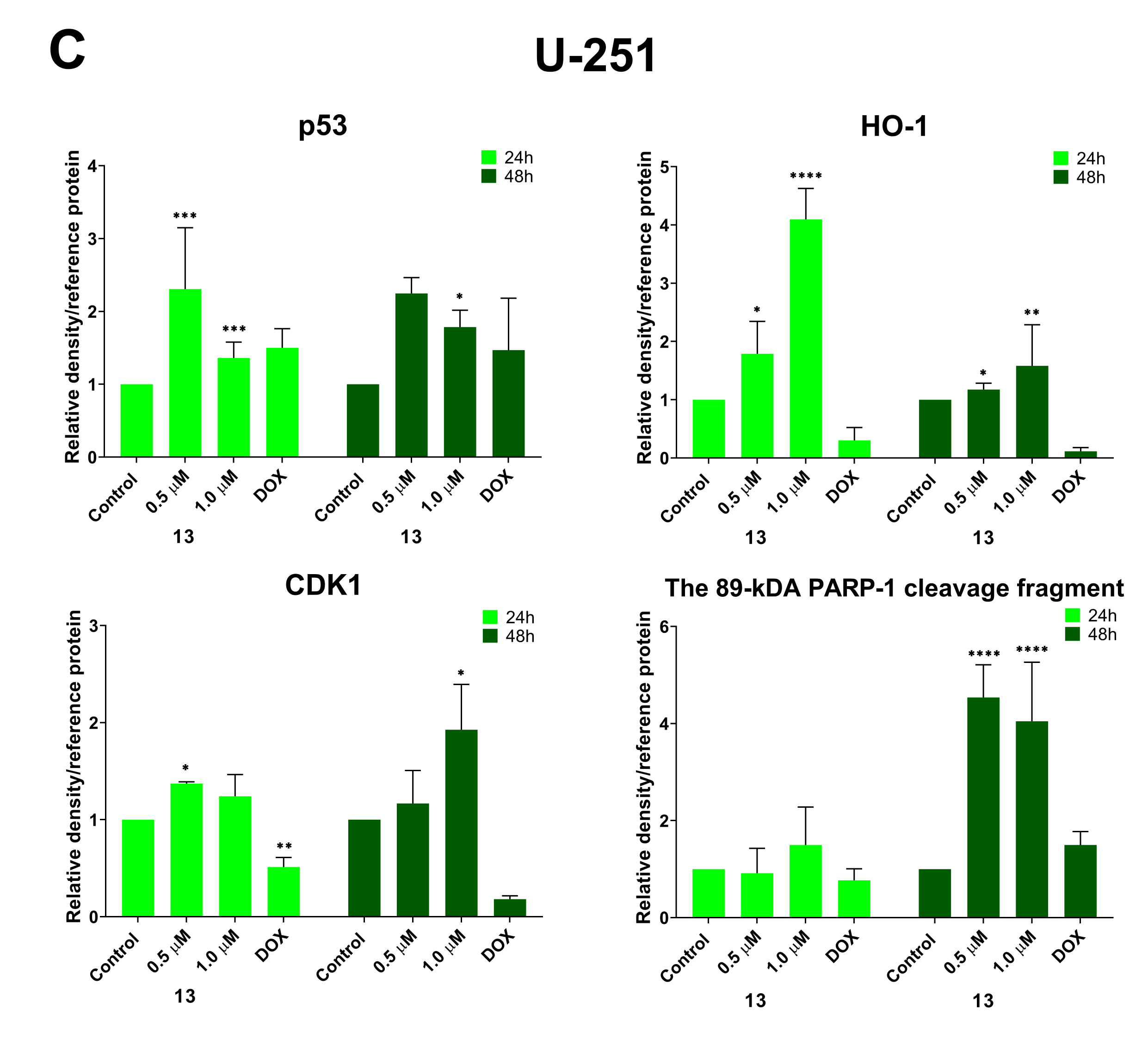

Supplement: Supplementary file 5 — Supplementary Material 5 [file 41598_2024_79698_MOESM5_ESM.tif]

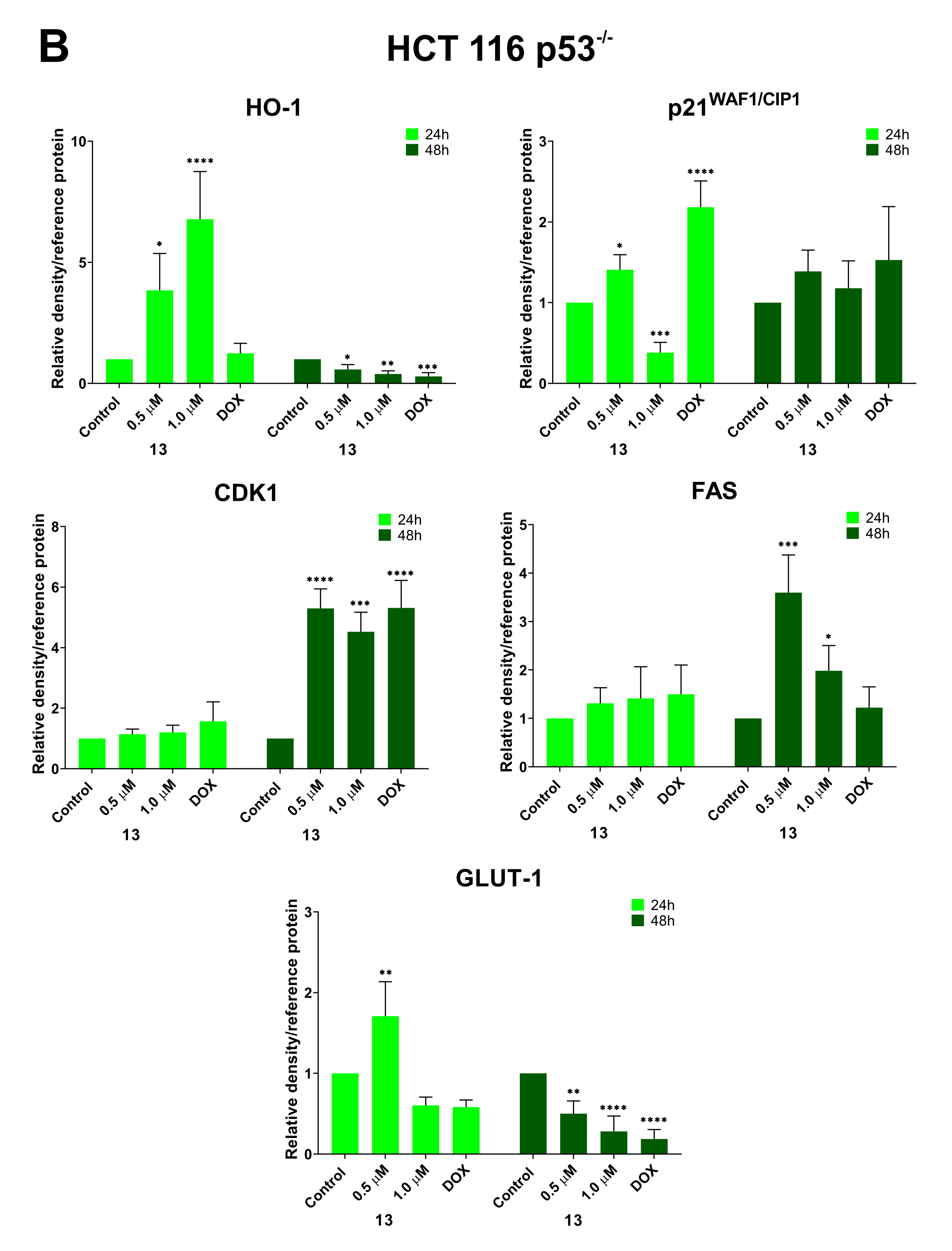

Supplement: Supplementary file 6 — Supplementary Material 6 [file 41598_2024_79698_MOESM6_ESM.tif]

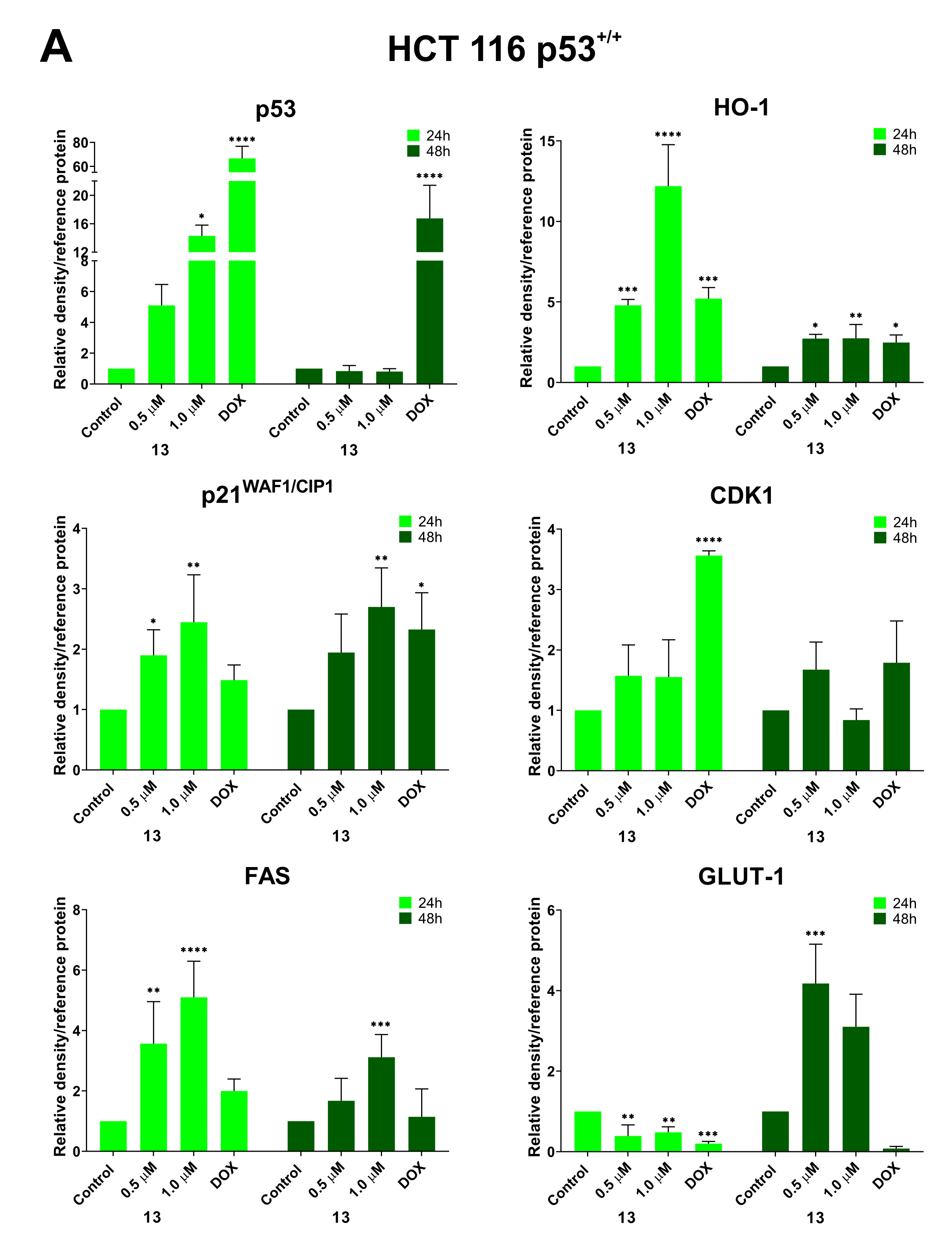

Supplement: Supplementary file 7 — Supplementary Material 7 [file 41598_2024_79698_MOESM7_ESM.tif]
