# Supplementary material for: 4-Furanylvinylquinoline derivative as a new scaffold for the design of oxidative stress initiator and glucose transporter inhibitor drugs
Source: Sci Rep. 2024 Nov 18;14:28454. doi: 10.1038/s41598-024-79698-0 (PMC11574108; doi:10.1038/s41598-024-79698-0)

**4-Furanylvinylquinoline derivative as a new scaffold for the design of oxidative stress initiator and glucose transporter inhibitor drugs.**

Michał Kuczak^1,2^, Wioleta Cieślik^2^, Robert Musioł^2^, Anna Mrozek-Wilczkiewicz^1,3*^

^1^A. Chelkowski Institute of Physics, University of Silesia in Katowice, 75 Pulku Piechoty 1a, 41-500 Chorzow, Poland

^2^Institute of Chemistry, University of Silesia in Katowice, Szkolna 9, 40-006 Katowice, Poland

^3^Department of Systems Biology and Engineering, Silesian University of Technology, Akademicka 2A, 44-100 Gliwice, Poland

corresponding author:

[*anna.mrozek-wilczkiewicz@us.edu.pl](mailto:*anna.mrozek-wilczkiewicz@us.edu.pl), anna.mrozek-wilczkiewicz@polsl.pl

**Keywords:** furanylvinylquinolines, cell cycle inhibition, p53 mutation, apoptosis, oxidative stress, GLUT receptor inhibitor, Warburg effect

**Table of Contents**

[**1.** **Chemistry** 2](#_Toc167132081)

[**2.** **Representative spectra** 6](#_Toc167132082)

[**2.1.** **^1^H NMR and ^13^C NMR spectra** 6](#_Toc167132083)

[**2.2.** **MS spectra** 13](#_Toc167132084)

[**3.** **Biological studies** 18](#_Toc167132085)

[**3.1.** **Cell cycle inhibition** 18](#_Toc167132086)

[**3.2.** **Apoptosis induction** 19](#_Toc167132087)

[**3.3.** **qRT-PCR** 20](#_Toc167132088)

[**3.4.** **Densitometric analysis of protein level** 21](#_Toc167132089)

[**3.5.** **Original uncropped membranes** 23](#_Toc167132090)

# **Chemistry**

**2-[(*E*)-2-(furan-2-yl)vinyl]quinoline-8-ol (1)** The compound was obtained using Method B and then crystallised from EtOH, resulting in a beige powder with a yield of 9% (53 mg); mp 83-85 ^o^C; **^1^H NMR** (500 MHz, DMSO-*d_6_*) δ 9.57 (bs, 1H, OH), 8.31 – 8.21 (m, 1H), 8.00 (d, *J* = 16.0 Hz, 1H), 7.83 – 7.69 (m, 2H), 7.43 – 7.30 (m, 2H), 7.22 (d, *J* = 16.0 Hz, 1H), 7.12 – 7.02 (m, 1H), 6.78 – 6.58 (m, 2H); **^13^C NMR** (126 MHz, DMSO-*d_6_*) δ 153.48, 153.35, 152.92, 144.54, 138.61, 136.95, 128.08, 127.48, 126.19, 122.48, 121.54, 118.01, 112.90, 112.15, 111.67; **LR-MS**: calculated for C_15_H_11_NO_2_ [M+H]^+^: 238.08625 *m/z*, found 238.0 *m/z*.

**2-[(*E*)-2-(5-methylfuran-2-yl)vinyl]quinolin-8-ol (2)** The compound was obtained using Method B and then crystallised from EtOH, resulting in a beige powder with a yield of 23% (144 mg); mp 96-98 ^o^C; **^1^H NMR** (500 MHz, DMSO-*d*_6_) δ 9.52 (bs, 1H, OH), 8.24 (d, *J* = 8.6 Hz, 1H), 7.93 (d, *J* = 16.1 Hz, 1H), 7.71 (d, *J* = 8.6 Hz, 1H), 7.36 (dt, *J* = 6.0, 2.3 Hz, 1H), 7.33 (dd, *J* = 8.2, 1.6 Hz, 1H), 7.12 (d, *J* = 16.1 Hz, 1H), 7.07 (dd, *J* = 7.2, 1.6 Hz, 1H), 6.60 (d, *J* = 3.1 Hz, 1H), 6.27 – 6.24 (m, 1H), 2.37 (s, 3H); **^13^C NMR** (126 MHz, DMSO-*d_6_*) δ 153.78, 153.27, 151.55, 138.60, 136.85, 127.95, 127.49, 127.29, 124.68, 122.51, 121.49, 117.98, 113.65, 111.57, 109.28, 14.06; **LR-MS**: calculated for C_16_H_13_NO_2_ [M+H]^+^: 252.10190 *m/z*, found 252.0 *m/z*.

**2-[(*E*)-2-(5-nitrofuran-2-yl)vinyl]quinolin-8-ol (3)** The compound was obtained using Method B and then crystallised from EtOH, resulting in a yellow powder with a yield of 48% (338 mg); mp 189-191 ^o^C; **^1^H NMR** (500 MHz, DMSO-*d*_6_) δ 9.77 (bs, 1H, OH), 8.34 (d, *J* = 8.5 Hz, 1H), 8.17 (d, *J* = 16.0 Hz, 1H), 7.84 (d, *J* = 8.5 Hz, 1H), 7.81 (d, *J* = 3.9 Hz, 1H), 7.63 (d, *J* = 16.0 Hz, 1H), 7.48 – 7.40 (m, 1H), 7.39 (dd, *J* = 8.2, 1.2 Hz, 1H), 7.14 (d, *J* = 3.9 Hz, 1H), 7.11 (dd, *J* = 7.5, 1.3 Hz, 1H); **^13^C NMR** (126 MHz, DMSO-*d_6_*) δ 156.00, 153.71, 152.02, 151.79, 138.74, 137.31, 133.27, 128.63, 128.43, 122.18, 120.55, 118.04, 115.96, 114.08, 111.93; **LR-MS**: calculated for C_15_H_10_N_2_O_2_ [M+H]^+^: 283.07133 *m/z*, found 283.0 *m/z*.

**2-[(*E*)-2-(5-nitrofuran-2-yl)vinyl]quinolin-8-yl acetate (4)** The compound was obtained using Method A and then crystallised from EtOH, resulting in an orange powder with a yield of 81% (656 mg); mp 173-175 ^o^C; **^1^H NMR** (500 MHz, Chloroform-*d*) δ 8.21 (d, *J* = 8.5 Hz, 1H), 7.73 (dd, *J* = 8.1, 1.1 Hz, 1H), 7.59 (d, *J* = 8.5 Hz, 1H), 7.57 – 7.52 (m, 3H), 7.48 (dd, *J* = 7.5, 1.2 Hz, 1H), 7.42 (t, *J* = 6.4 Hz, 1H), 6.70 (t, *J* = 10.0 Hz, 1H), 2.58 (s, 3H); **^13^C NMR** (126 MHz, Chloroform-*d*) δ 169.75, 155.10, 153.50, 151.78, 147.54, 141.14, 136.90, 133.46, 129.06, 126.60, 125.67, 122.03, 121.41, 119.42, 113.83, 112.56, 21.02; **LR-MS**: calculated for C_17_H_12_N_2_O_5_ [M+H]^+^: 325.08190 *m/z*, found 325.0 *m/z*.

**2-[(*E*)-2-(5-nitrofuran-2-yl)vinyl]quinolin-4-ol (5)** The compound was obtained using Method B and then crystallised from MeOH, resulting in a yellow powder with a yield of 55% (388 mg); mp 306-308 ^o^C; **^1^H NMR** (500 MHz, DMSO-*d*_6_) δ 11.69 (s, 1H), 8.06 (dd, *J* = 8.1, 1.2 Hz, 2H), 7.81 (d, *J* = 3.9 Hz, 1H), 7.73 – 7.57 (m, 3H), 7.36 – 7.29 (m, 1H), 7.25 (d, *J* = 16.5 Hz, 1H), 7.15 (d, *J* = 3.9 Hz, 1H), 6.48 (d, *J* = 0.9 Hz, 1H); **^13^C NMR** (126 MHz, DMSO-*d_6_*) δ 177.93, 154.33, 152.09, 146.27, 141.11, 132.68, 126.92, 126.01, 125.21, 123.89, 121.27, 118.81, 115.66, 115.21, 108.29; **LR-MS**: calculated for C_15_H_10_N_2_O_4_ [M+H]^+^: 283.07133 *m/z*, found 283.0 *m/z*.

**5,7-dichloro-2-[2-(furan-2yl)vinyl]-quinolin-8-ol (6)** The compound was obtained using Method B and then crystallised from EtOH, resulting in a brown powder with a yield of 10% (77 mg); mp 253-255 ^o^C; **^1^H NMR** (500 MHz, DMSO-*d_6_*) δ 10.48 (bs, 1H, OH), 8.43 (d, *J* = 8.7 Hz, 1H), 8.18 (d, *J* = 15.9 Hz, 1H), 7.90 (d, *J* = 8.7 Hz, 1H), 7.84 (d, *J* = 1.3 Hz, 1H), 7.73 (s, 1H), 7.26 (d, *J* = 15.9 Hz, 1H), 6.79 (d, *J* = 3.3 Hz, 1H), 6.66 (dd, *J* = 3.3, 1.8 Hz, 1H); **^13^C NMR** (126 MHz, DMSO-*d_6_*) δ 155.35, 152.72, 149.10, 145.07, 139.26, 133.79, 127.39, 125.07, 124.28, 124.01, 123.02, 119.59, 115.87, 113.10; **LR-MS**: calculated for C_15_H_9_Cl_2_NO_2_ [M+H]^+^: 306.00831 *m/z*, found 306.0 *m/z*.

**5,7-dichloro-2-[2-(5-nitrofuran-2yl)vinyl]-quinolin-8-ol (7)** The compound was obtained using Method B and then crystallised from EtOH, resulting in a beige powder with a yield of 84% (737 mg); mp 270-271 ^o^C; **^1^H NMR** (500 MHz, DMSO-*d*_6_) δ 10.71 (bs, 1H, OH), 8.51 (d, *J* = 8.7 Hz, 1H), 8.33 (d, *J* = 16.0 Hz, 1H), 7.99 (d, *J* = 8.8 Hz, 1H), 7.82 (d, *J* = 3.9 Hz, 1H), 7.80 (s, 1H), 7.65 (d, *J* = 16.0 Hz, 1H), 7.17 (d, *J* = 3.9 Hz, 1H); **^13^C NMR** (126 MHz, DMSO-*d_6_*) δ 155.57, 153.79, 151.91, 149.37, 139.19, 134.17, 132.05, 128.21, 124.50, 123.50, 122.24, 119.56, 116.06, 115.80, 114.61; **LR-MS**: calculated for C_15_H_8_Cl_2_N_2_O_4_ [M+H]^+^: 350.99339 *m/z*, found 351.0 *m/z*.

**5,7-dichloro-2-[2-(5-nitrofuran-2yl)vinyl]-quinolin-8-yl acetate (8)** The compound was obtained using Method A and then crystallised from EtOH, resulting in a beige powder with a yield of 77% (757 mg); mp 220-222 ^o^C; **^1^H NMR** (500 MHz, DMSO-*d*_6_) δ 8.61 (d, *J* = 8.8 Hz, 1H), 8.13 (d, *J* = 8.8 Hz, 1H), 8.07 (s, 1H), 7.83 (d, *J* = 3.9 Hz, 1H), 7.80 (d, *J* = 16.2 Hz, 1H), 7.58 (d, *J* = 16.2 Hz, 1H), 7.29 (d, *J* = 3.9 Hz, 1H), 2.58 (s, 3H); **^13^C NMR** (126 MHz, DMSO-*d_6_*) δ 168.74, 156.02, 154.92, 152.09, 143.12, 141.89, 134.52, 132.09, 128.73, 127.46, 127.16, 125.29, 123.46, 122.44, 122.20, 115.75, 20.76; **LR-MS**: calculated for C_17_H_10_Cl_2_N_2_O_5_ [M+H]^+^: 393.00395 *m/z*, found 393.0 *m/z*.

**4-[(*E*)-2-(2-nitrophenyl)vinyl]quinoline (9)** The compound was obtained using Method A and then crystallised from EtOAc, resulting in a beige powder with a yield of 32 % (221 mg); mp 161-163 ^o^C; **^1^H NMR** (500 MHz, DMSO-*d*_6_) δ 8.94 (d, *J* = 4.6 Hz, 1H), 8.52 (d, *J* = 8.5 Hz, 1H), 8.25 (d, *J* = 8.5 Hz, 1H), 8.12 (d, *J* = 16.0 Hz, 1H), 8.07 (d, *J* = 8.4 Hz, 2H), 7.87 – 7.83 (m, 1H), 7.83 – 7.76 (m, 3H), 7.69 (ddd, *J* = 8.4, 6.8, 1.4 Hz, 1H), 7.65 (ddd, *J* = 8.4, 6.8, 1.4 Hz, 1H). **^13^C NMR** (126 MHz, DMSO) δ 150.86, 148.71, 148.60, 142.06, 134.16, 131.77, 130.34, 130.04, 129.67, 127.79, 127.30, 126.14, 125.07, 124.65, 117.95. **HR-MS**: calculated for C_17_H_12_N_2_O_2_ [M+H]^+^: 277.097154 *m/z*, found 277.09653 *m/z*.

**4-[(*E*)-2-(3-nitrophenyl)vinyl]quinoline (10)** The compound was obtained using Method A and then crystallised from EtOAc, resulting in a beige powder with a yield of 33% (228 mg); mp 111-112 ^o^C; **^1^H NMR** (500 MHz, DMSO) δ 8.92 (d, *J* = 4.6 Hz, 1H), 8.68 (s, 1H), 8.60 (d, *J* = 8.2 Hz, 1H), 8.34-8.31 (m, 2H), 8.19 (dd, *J* = 8.2, 1.6 Hz, 1H), 8.06 (d, *J* = 8.2 Hz, 1H), 7.88 (d, *J* = 4.6 Hz, 1H), 7.81 (dt, *J* = 8.2, 2.9 Hz, 1H), 7.78 – 7.71 (m, 2H), 7.71 – 7.65 (m, 1H). **^13^C NMR** (126 MHz, DMSO) δ 150.72, 148.89, 148.77, 142.11, 138.81, 134.03, 133.34, 130.69, 130.02, 129.96, 127.15, 126.24, 125.91, 124.81, 123.49, 122.42, 117.50. **HR-MS**: calculated for C_17_H_12_N_2_O_2_ [M+H]^+^: 277.097154 *m/z*, found 277.09692 *m/z*.

**4-[(*E*)-2-(4-nitrophenyl)vinyl]quinoline (11)** The compound was obtained using Method A and then crystallised from EtOAc, resulting in a yellow powder with a yield of 41% (283 mg); mp 228-229 ^o^C; **^1^H NMR** (500 MHz, DMSO-*d*_6_) δ 8.94 (d, *J* = 8.9 Hz, 1H), 8.58 (dd, *J* = 9.5, 1.9 Hz, 1H), 8.37 (d, *J* = 16.2 Hz, 1H), 8.30 (d, *J* = 8.9 Hz, 2H), 8.13 (d, *J* = 8.9 Hz, 2H), 8.07 (dd, *J* = 9.5, 1.9 Hz, 1H), 7.92 (d, *J* = 4.6 Hz, 1H), 7.82 (ddd, *J* = 8.2, 6.8, 1.4 Hz, 1H), 7.76 (d, *J* = 16.2 Hz, 1H), 7.70 (ddd, *J* = 8.2, 6.8, 1.4 Hz, 1H). **^13^C NMR** (126 MHz, DMSO-*d*_6_) δ 150.76, 148.78, 147.42, 143.58, 141.83, 133.33, 130.27, 130.08, 130.02, 128.94, 127.59, 127.30, 126.19, 124.64, 124.46, 124.08, 117.66. **HR-MS**: calculated for C_17_H_12_N_2_O_2_ [M+H]^+^: 277.097154 *m/z*, found 277.0979 *m/z*.

**4-[(*E*)-2-(2,4-dinitrophenyl)vinyl]quinoline (12)** The compound was obtained using Method A and then crystallised from EtOAc, resulting in a yellow powder with a yield of 43% (345 mg); mp 200-201 ^o^C; **^1^H NMR** (500 MHz, DMSO-*d*_6_) δ 8.97 (d, *J* = 4.6 Hz, 1H), 8.81 (d, *J* = 2.3 Hz, 1H), 8.59 (dd, *J* = 8.7, 2.3 Hz, 1H), 8.57 – 8.53 (m, 2H), 8.36 (d, *J* = 16.0 Hz, 1H), 8.09 (dd, *J* = 8.4, 1.3 Hz, 1H), 7.88 – 7.81 (m, 3H), 7.72 (ddd, *J* = 8.3, 6.8, 1.4 Hz, 1H). **^13^C NMR** (126 MHz, DMSO-*d*_6_) δ 150.93, 148.74, 148.22, 147.17, 141.44, 137.54, 131.48, 131.13, 130.24, 130.08, 128.75, 127.93, 127.52, 126.07, 124.66, 120.78, 118.30. **HR-MS:** calculated for C_17_H_11_N_3_O_4_ [M+H]^+^: 322.082232 *m/z*, found 322.08185 *m/z*.

**4-[(*E*)-2-(5-nitrofuran-2-yl)vinyl]quinoline (13)** The compound was obtained using Method A and then crystallised from EtOH, resulting in a yellow powder with a yield of 45% (299 mg); mp 147-148 ^o^C; **^1^H NMR** (500 MHz, DMSO-*d*_6_) δ 8.94 (d, *J* = 4.6 Hz, 1H), 8.45 (dd, *J* = 8.4, 1.3 Hz, 1H), 8.22 (d, *J* = 16.2 Hz, 1H), 8.08 (dd, *J* = 8.4, 1.3 Hz, 1H), 7.93 (d, *J* = 4.7 Hz, 1H), 7.86 – 7.81 (m, 2H), 7.72 (ddd, *J* = 8.4, 6.8, 1.3 Hz, 1H), 7.64 (d, *J* = 16.2 Hz, 1H), 7.35 (d, *J* = 4.6 Hz, 1H). **^13^C NMR** (126 MHz, DMSO-*d*_6_) δ 155.79, 151.82, 150.73, 148.79, 140.54, 130.16, 130.12, 128.05, 127.58, 125.82, 124.15, 121.49, 117.67, 115.91, 114.00. **HR-MS**: calculated for C_15_H_10_N_2_O_3_ [M+H]^+^: 267.076419 *m/z*, found 267.0775 *m/z*.

# **Representative spectra**

## **^1^H NMR and ^13^C NMR spectra**

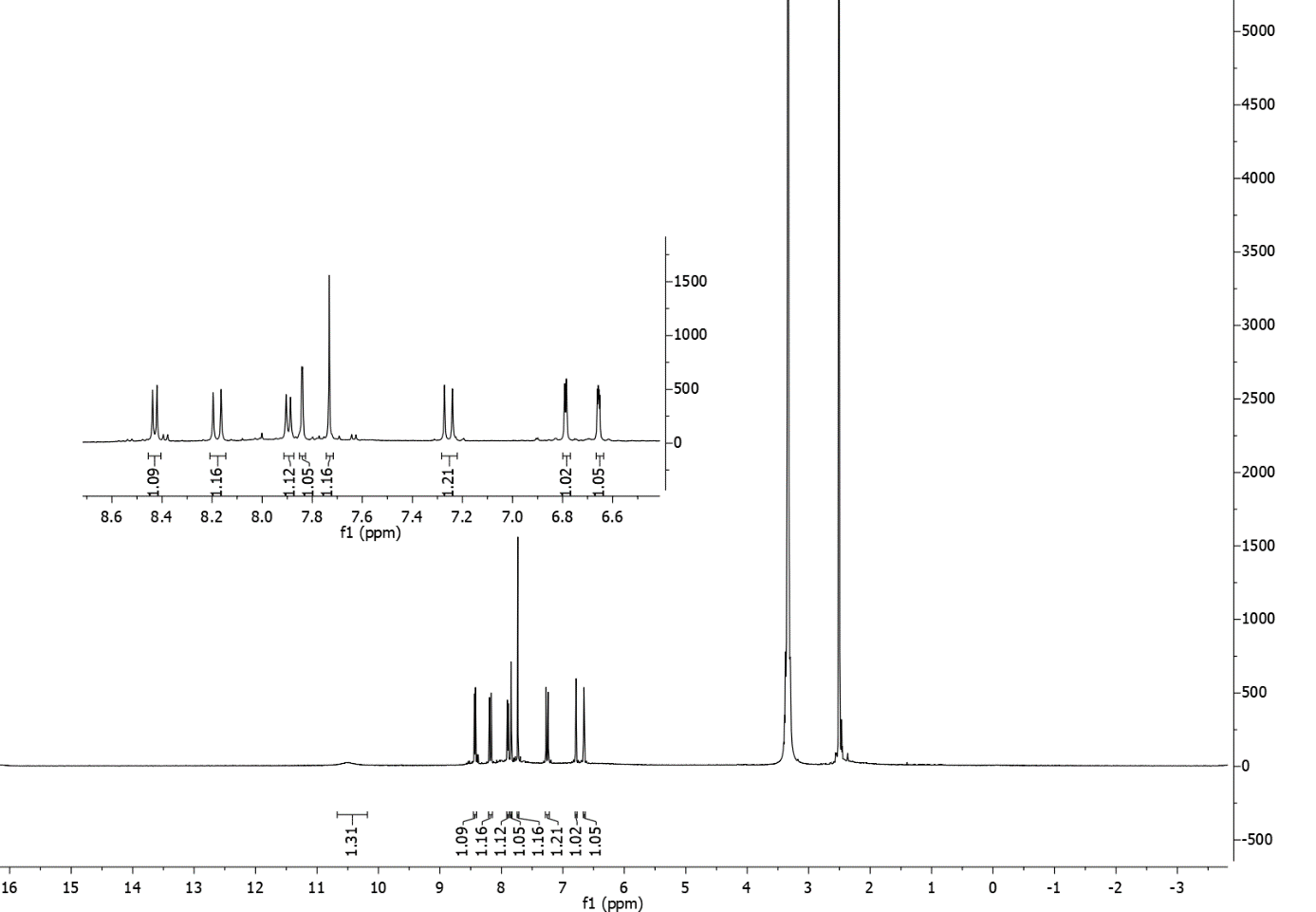


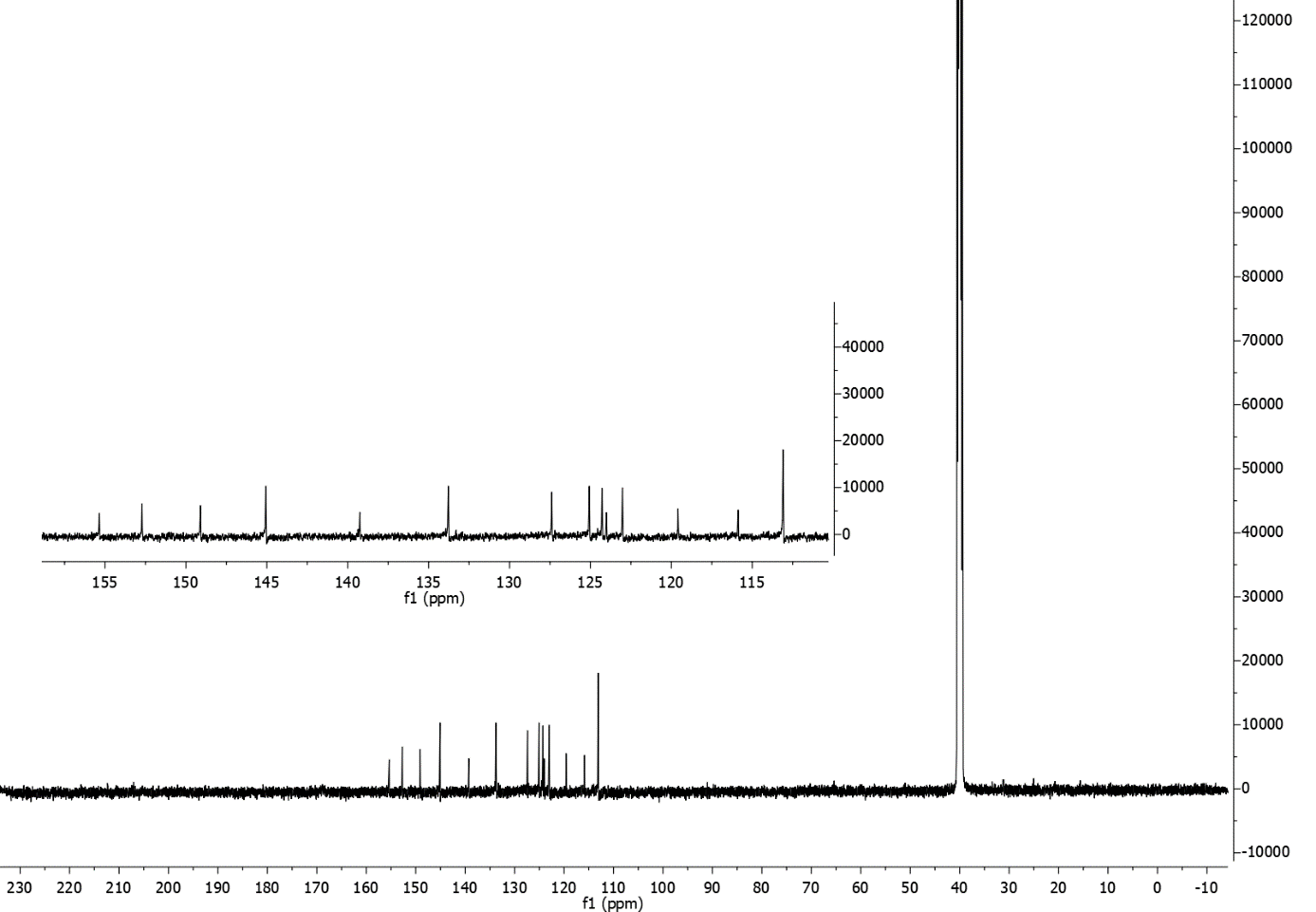

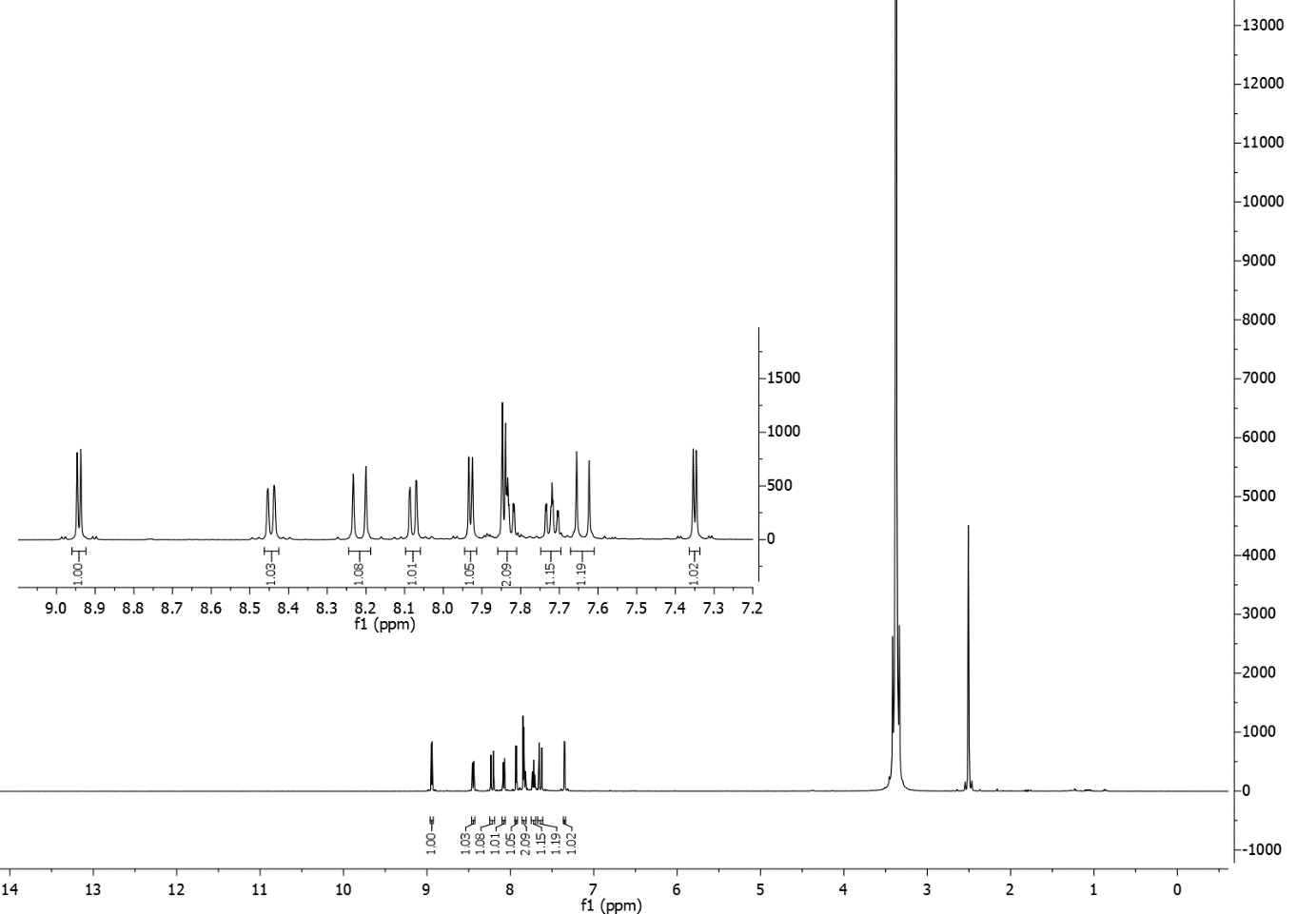

## **MS spectra**


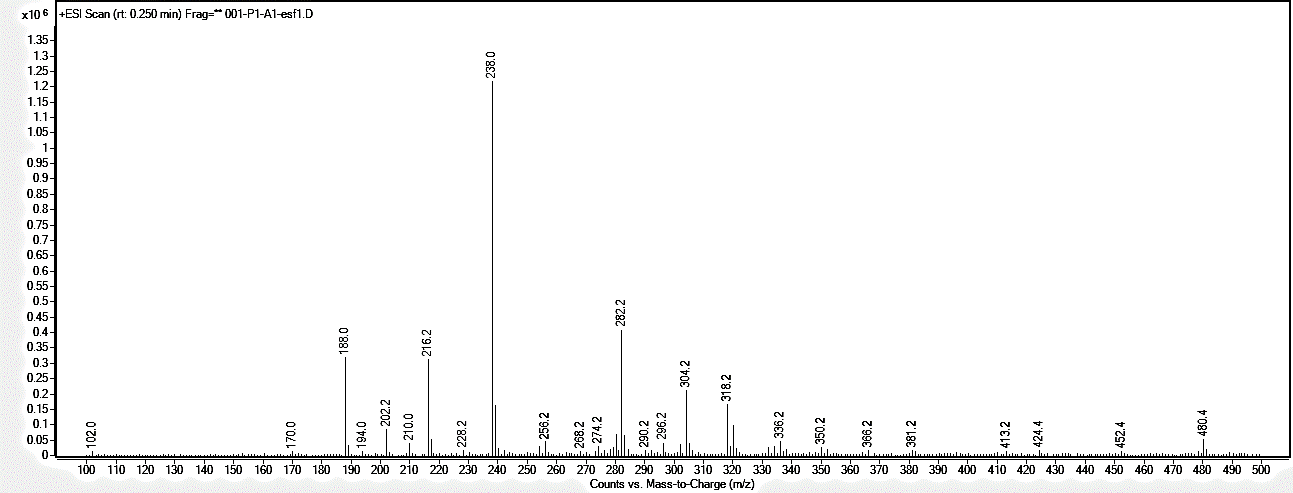


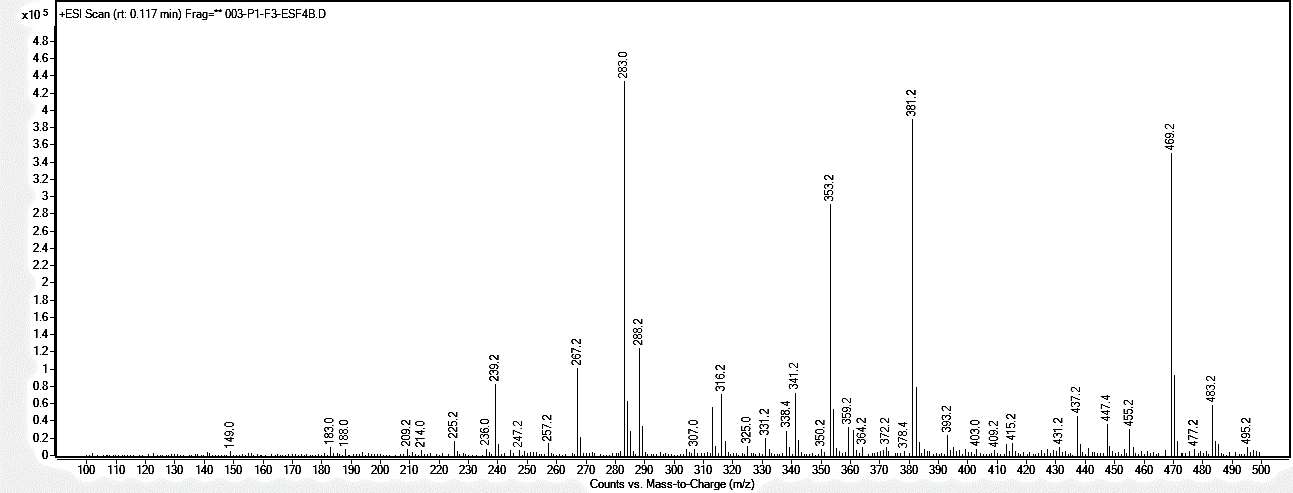


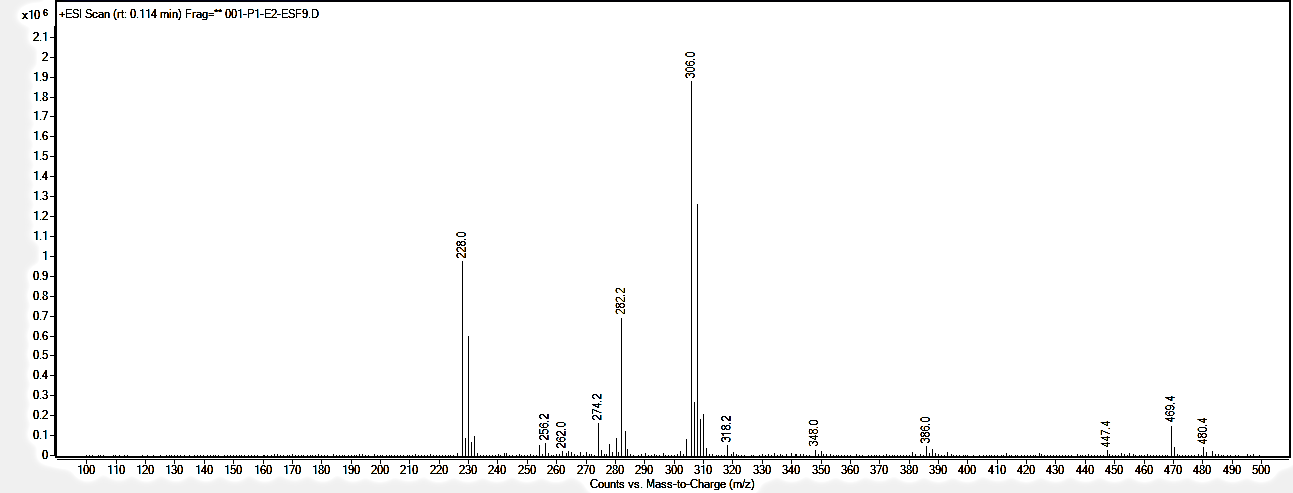


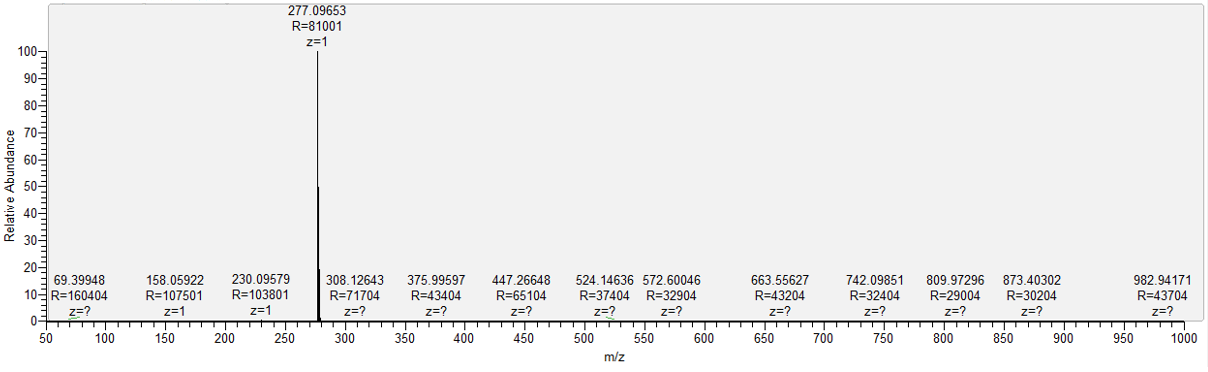


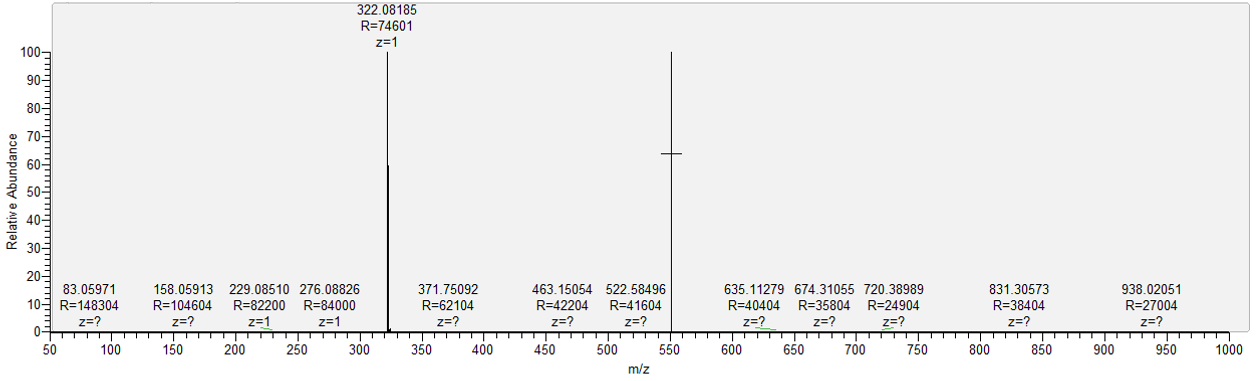


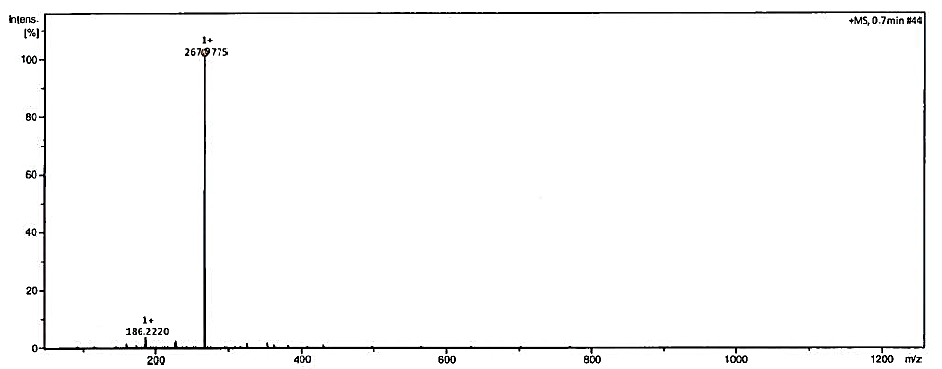


#

# **Biological studies**

## **Cell cycle inhibition**

**
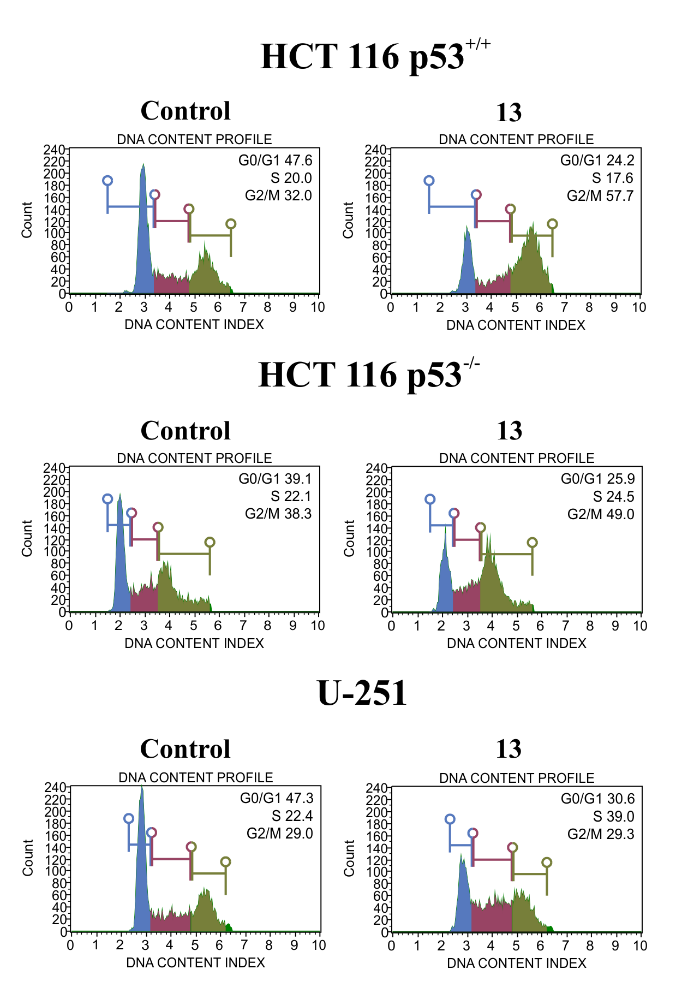
**

**Figure S1**. The representative histograms from flow cytometry show the distribution of the cells (HCT 116 p53^+/+^, HCT 116 p53^-/-^, U-251) in the particular cell cycle phases after 24-h incubation with **13** (concentration 2xIC_50_).


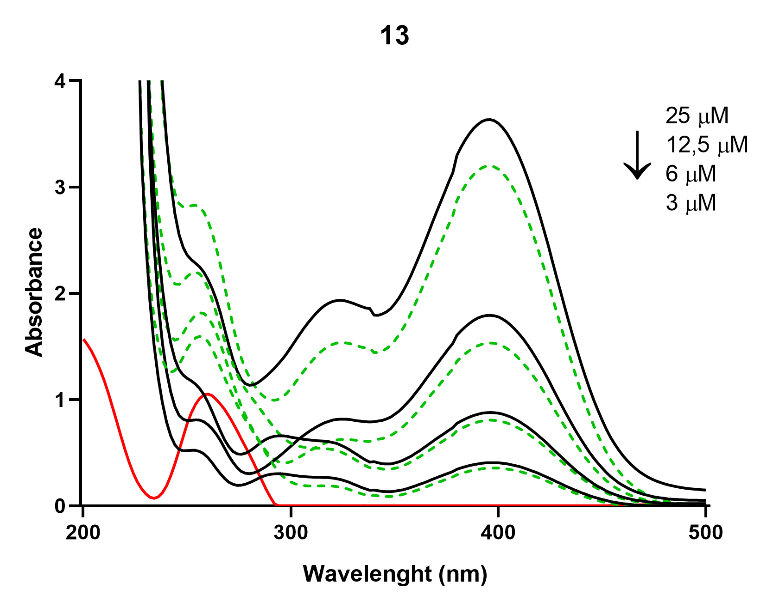


**Figure** **S2**. Absorption spectra of **13** (black, solid line) in decreasing concentration, CT-DNA (red, solid line) and a mixture of **13** with CT-DNA measured after 1.5 h. Absorption λmax 396 nm, Δε M^−1^ cm^−1^ 15541,67.

## **Apoptosis induction**

**
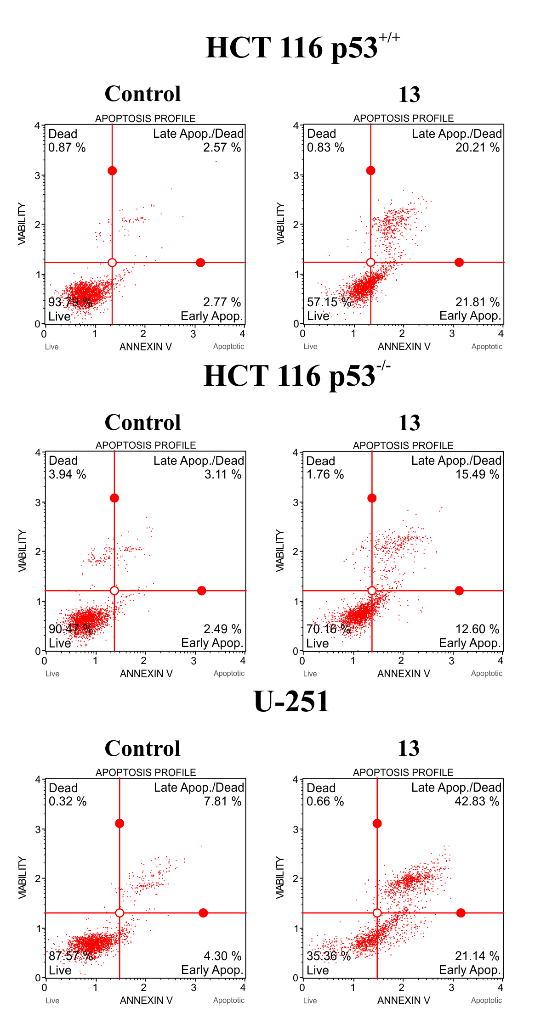
**

**Figure S3**. The representative histograms from flow cytometry show the percentage of life, apoptotic (early and late), and dead cells (HCT 116 p53^+/+^, HCT 116 p53^-/-^, U-251 after 48-h incubation with **13** (concentration 0.5 µM).

## **qRT-PCR**

**Table S1**. Sequences of primers used in Real-Time PCR reaction to determine mRNA expression of of selected genes.

| Gene | Sequence number in the GenBank database | Starter Forward (5´→3´) | Starter Reverse (5´→3´) |
| --- | --- | --- | --- |
| CDK1 | NM_001170407.1 | ATTGAGCGGAGAGCGACG | CCACTTGACCTGTAGTTTTGTGT |
| TRAIL | NM_003810.4 | AACTCCGTCAGCTCGTTAGA | GTGTTGCTTCTTCCTCTGGTC |
| DR5 | NM_003842.5 | CCCTGTTCTCTCTCAGGCATC | TCGTTGTGAGCTTCTGTCCA |
| CASP-8 | NM_001080125.2 | GAGTCATCTCTGTTCTGCTTTAGG | CTGTGAAAACACTTCCCTCCAG |
| MnSOD | NM_001024465.1 | AAACCTCAGCCCTAACGGTG | CCAGGCTTGATGCACATCTTA |
| CAT | NM_001752.3 | ACTGTTGCTGGAGAATCGGG | AAGTCTCGCCGCATCTTCAA |
| GLUT-1 | NM_006516.3 | AGCAACTGTGTGGTCCCTAC | GCTCCTCGGGTGTCTTGTC |
| GLUT-3 | NM_006931.3 | CGTGGAGAAAACTTGCTGCTG | CTGTGTCCCCATCGCTGTAA |
| GLUT-4 | NM_001042.3 | CATTCCTTGGTTCATCGTGGC | TAGCCTCCGCAACATACTGG |
| GADPH | NM_002046 | GAGTCAACGGATTTGGTCGTA | GCCCCACTTGATTTTGGAG |

## **Densitometric analysis of protein level**


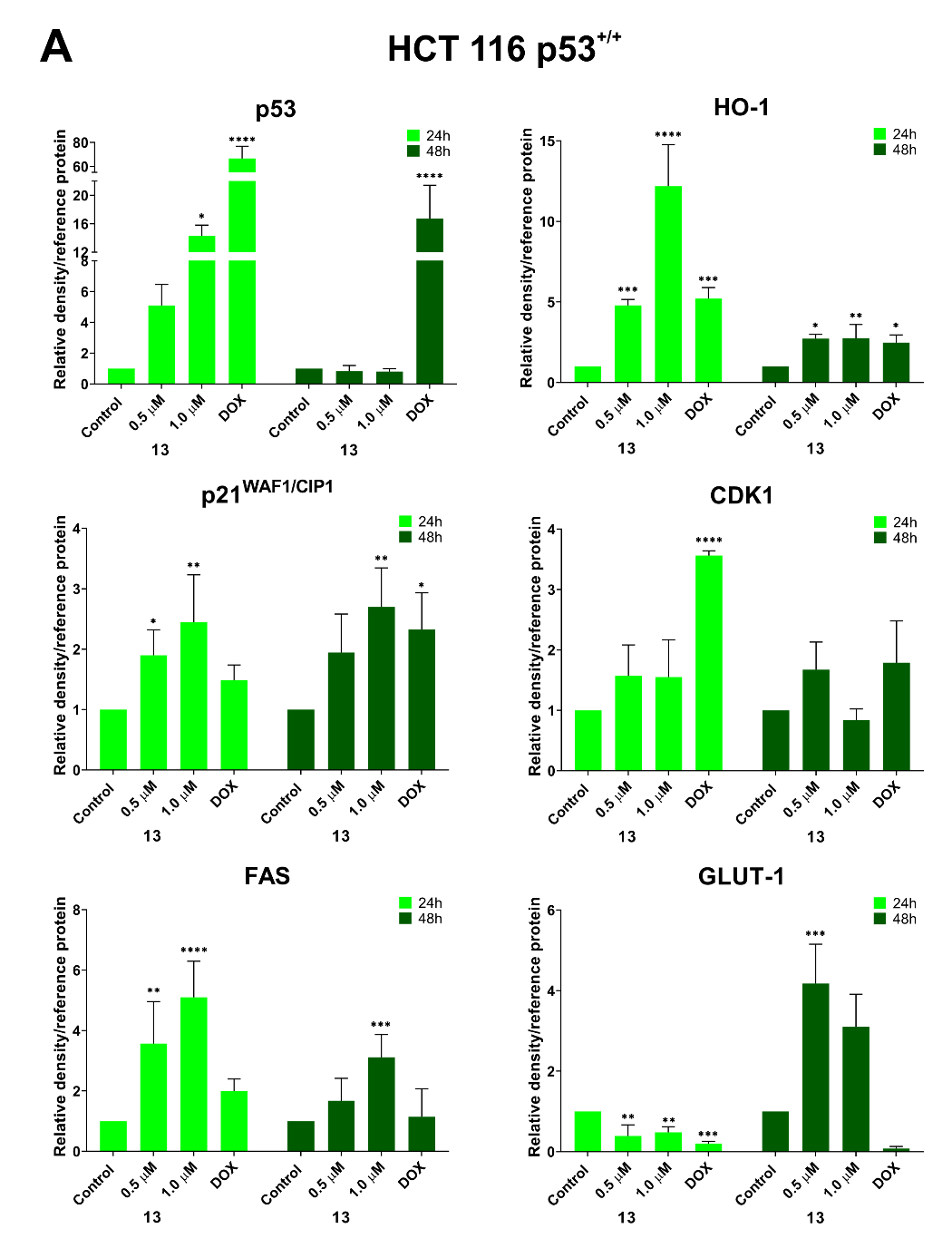


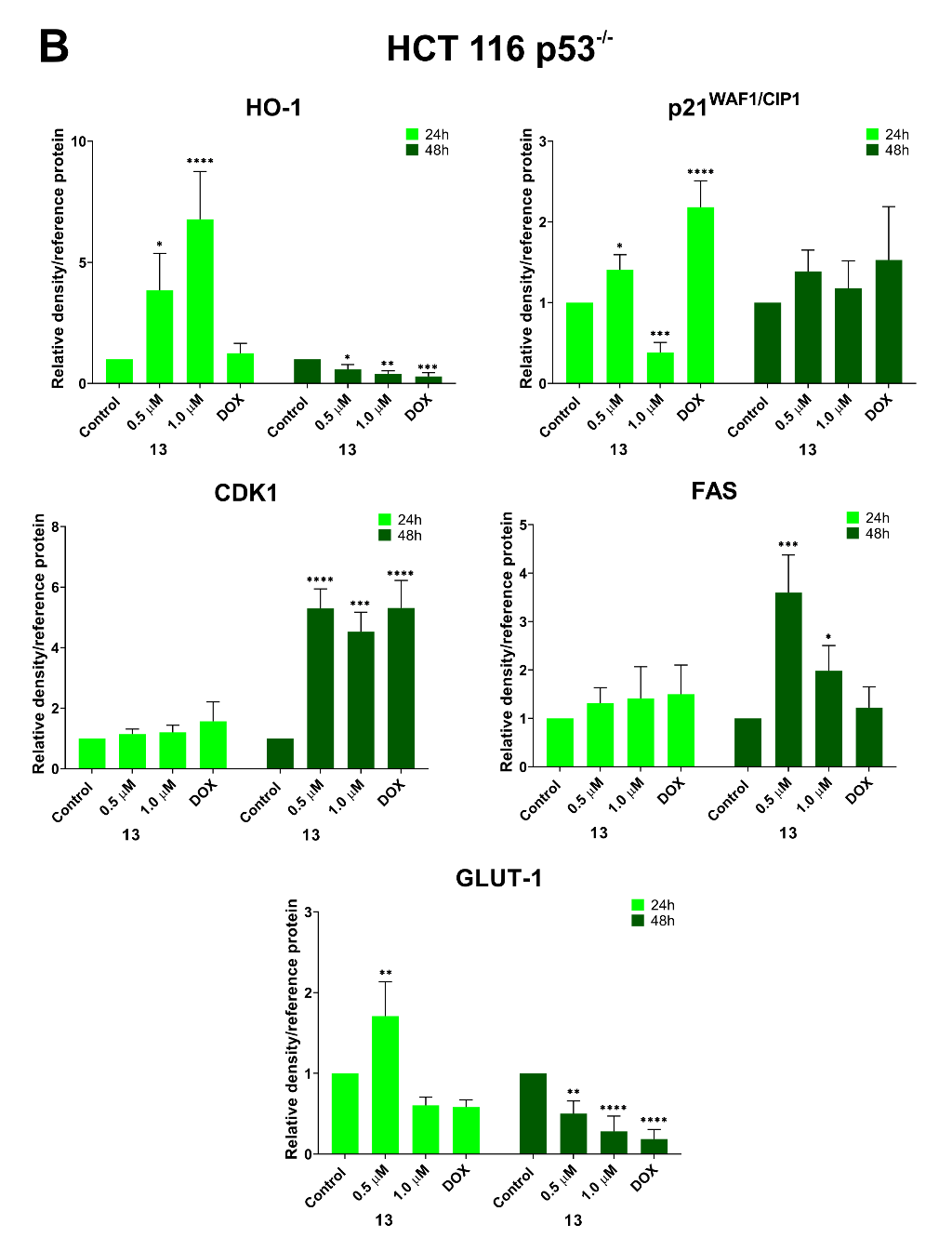


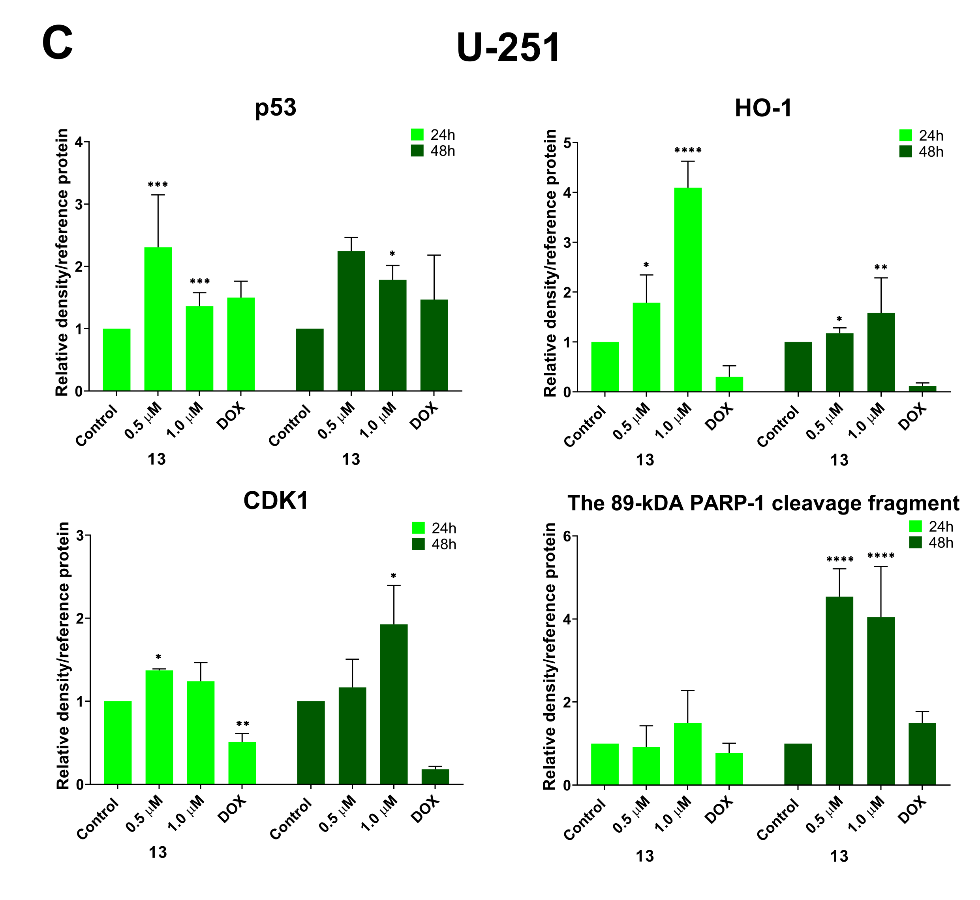


**Figure S4**. The charts present the densitometric analysis of results in HCT 116 p53^+/+^ (**A**), HCT 116 p53^-/-^ (**B**), and U-251(**C**) cell lines (as mean ± SD) of several independent experiments. Data were normalized to reference proteins. The statistical analysis was performed using one-way ANOVA with Bonferroni’s post-hoc test: *p<0.05, **p<0.01, ***p<0.001, ****p<0.0001 compared to the untreated cells (control).

## **Original uncropped membranes**


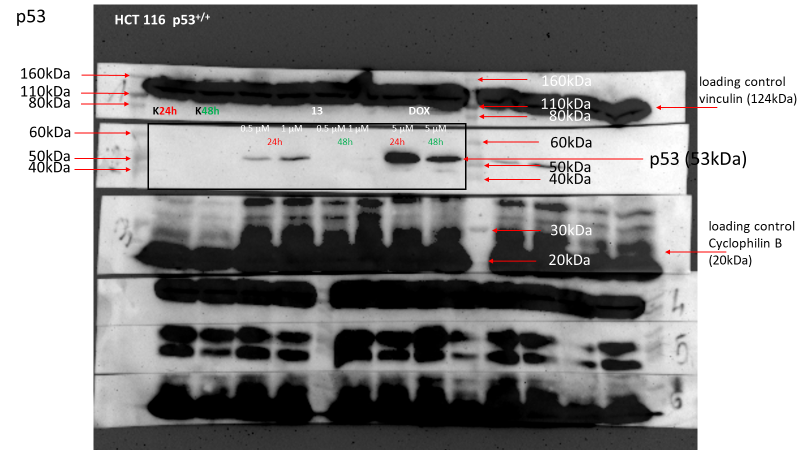


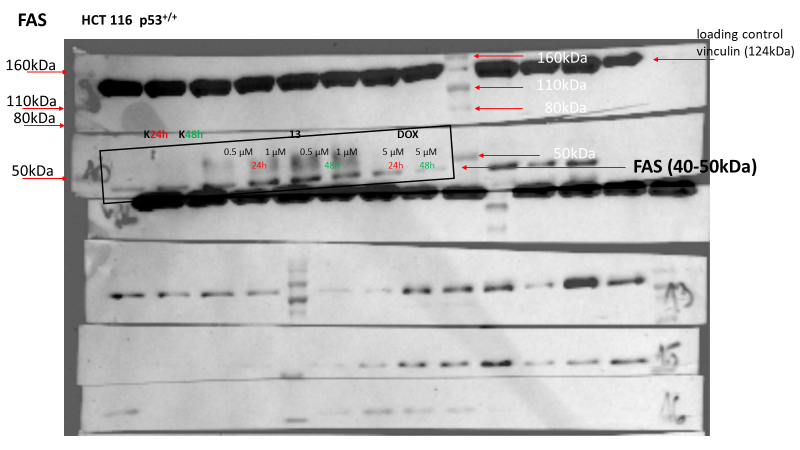

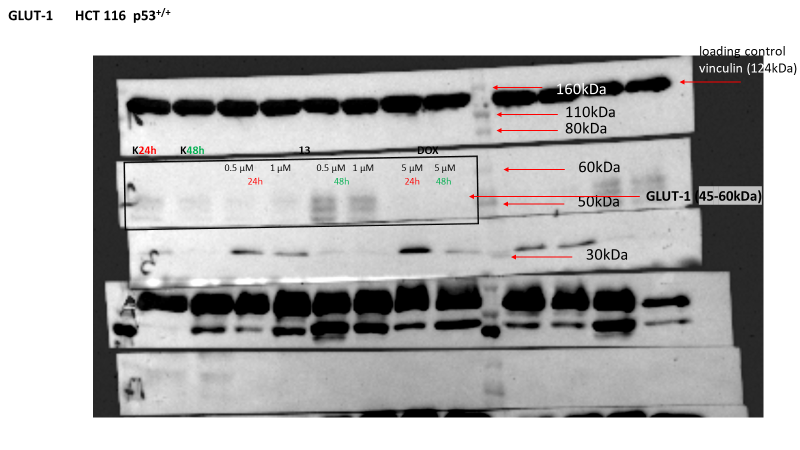

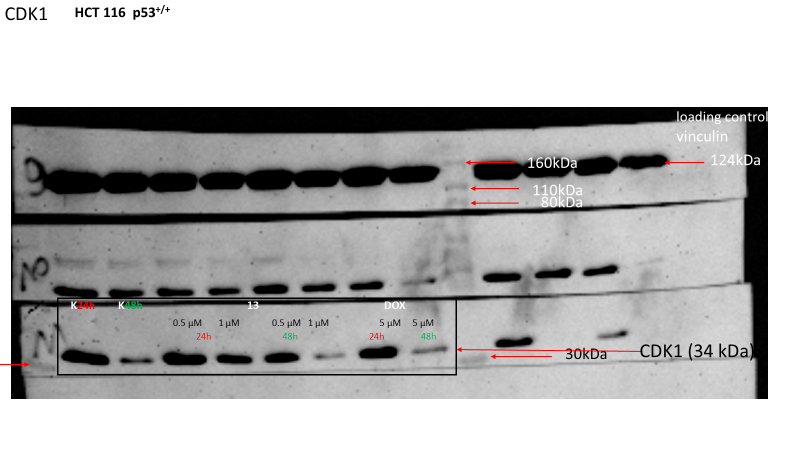

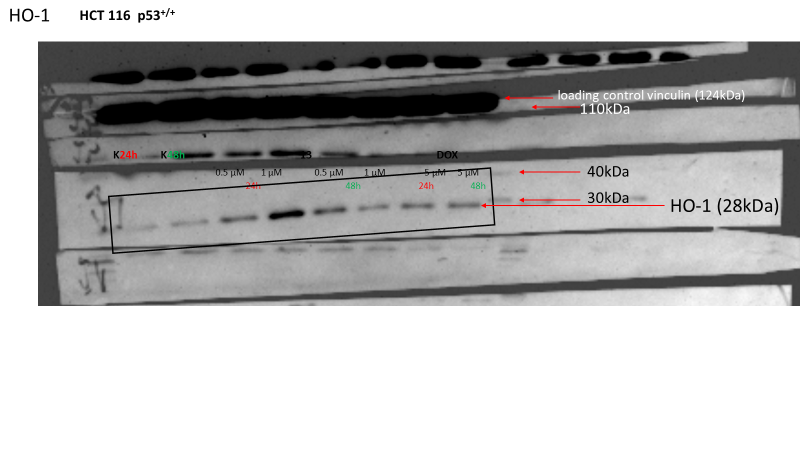

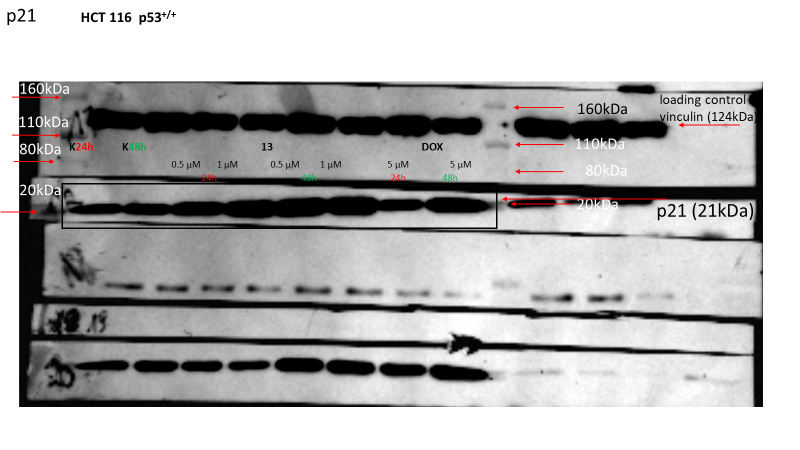

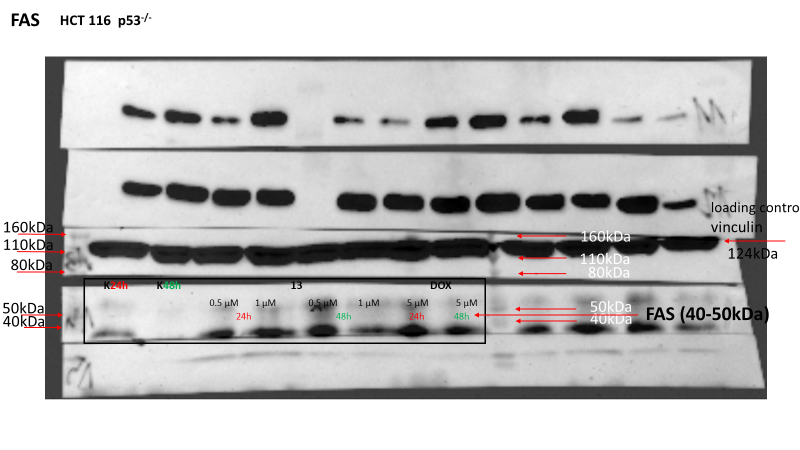

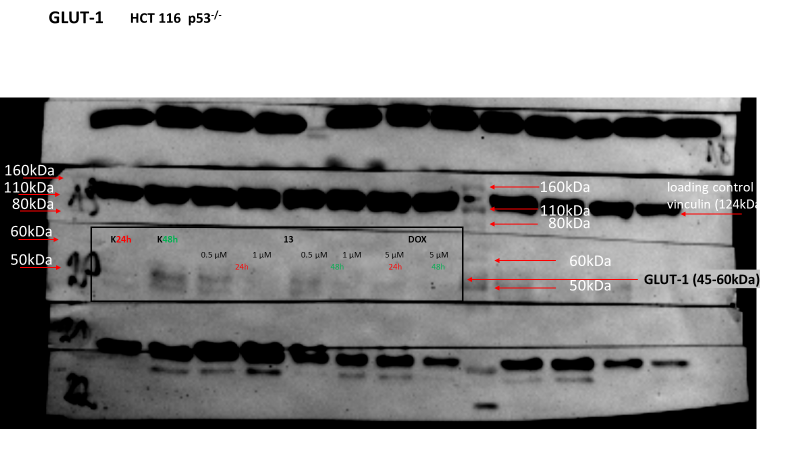

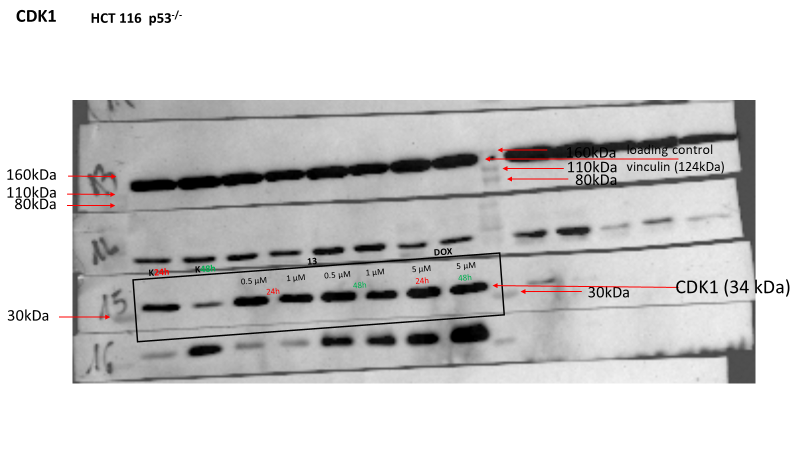

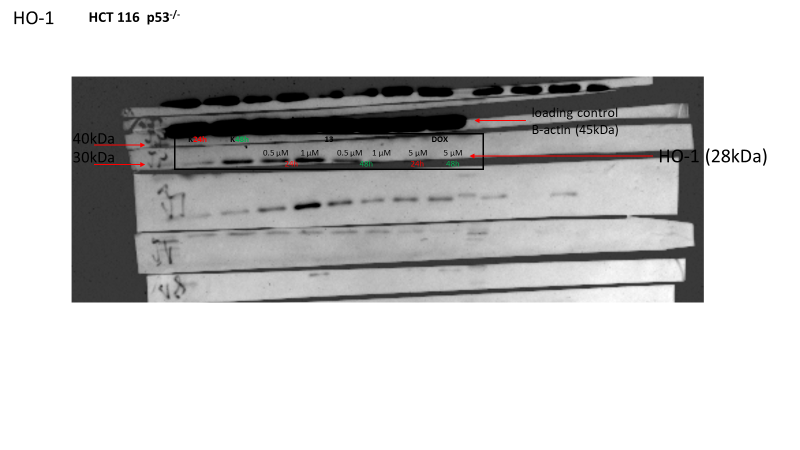

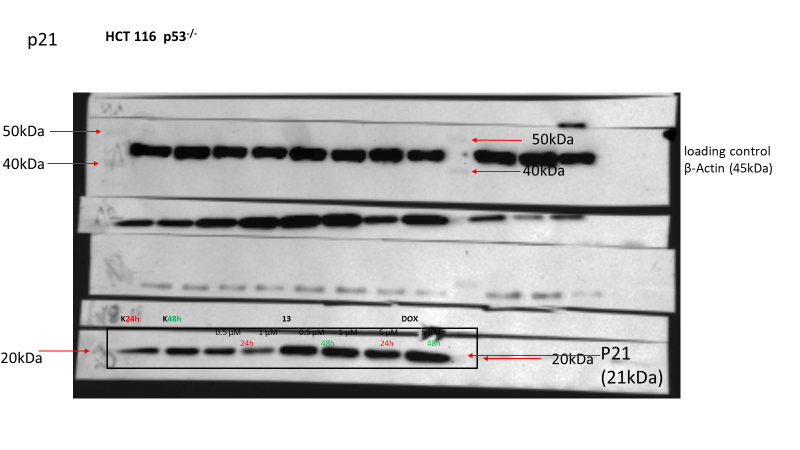


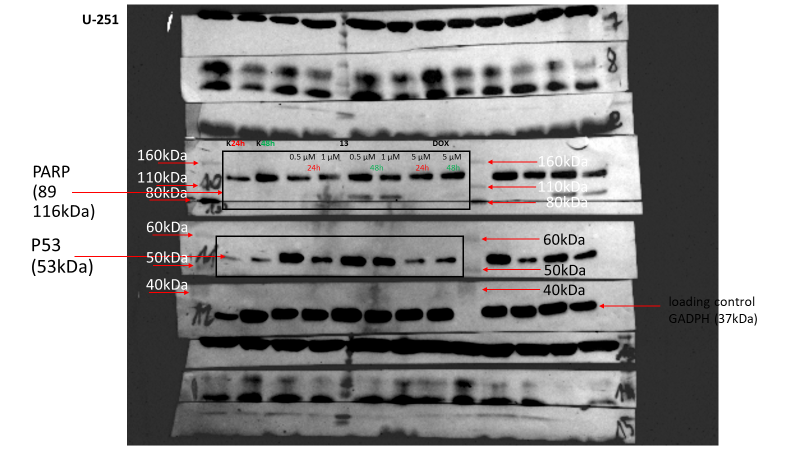

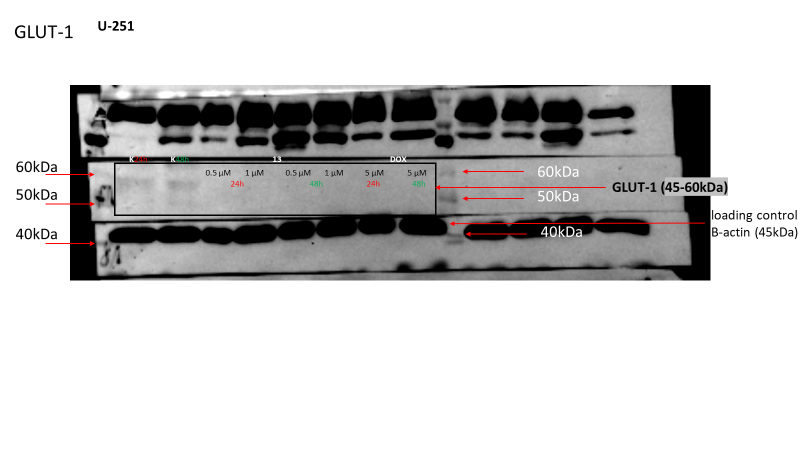

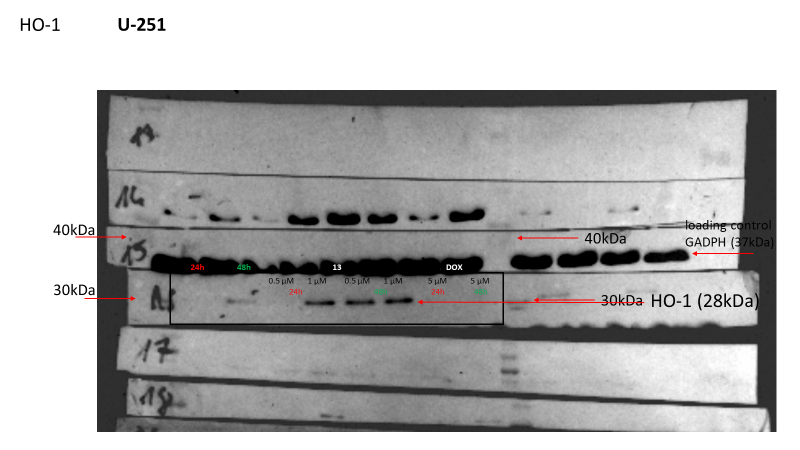

Supplement: Supplementary file 8 — Supplementary Material 8 [file 41598_2024_79698_MOESM8_ESM.docx]
